# Concerted changes in transcriptional regulation of genes involved in DNA methylation, demethylation, and folate-mediated one-carbon metabolism pathways in the NCI-60 cancer cell line panel in response to cancer drug treatment

**DOI:** 10.1186/s13148-016-0240-3

**Published:** 2016-06-24

**Authors:** Julia Krushkal, Yingdong Zhao, Curtis Hose, Anne Monks, James H. Doroshow, Richard Simon

**Affiliations:** Biometric Research Program, Division of Cancer Treatment and Diagnosis, National Cancer Institute, 9609 Medical Center Dr., Rockville, MD 20850 USA; Molecular Pharmacology Group, Leidos Biomedical Research, Inc., Frederick National Laboratory for Cancer Research, Frederick, MD 21702 USA; Division of Cancer Treatment and Diagnosis, National Cancer Institute, Bethesda, MD 20892 USA

**Keywords:** Gene expression, DNA methylation, Folate metabolism, Cancer drug treatment, Epigenetic analysis, NCI-60 cell lines

## Abstract

**Background:**

Aberrant patterns of DNA methylation are abundant in cancer, and epigenetic pathways are increasingly being targeted in cancer drug treatment. Genetic components of the folate-mediated one-carbon metabolism pathway can affect DNA methylation and other vital cell functions, including DNA synthesis, amino acid biosynthesis, and cell growth.

**Results:**

We used a bioinformatics tool, the Transcriptional Pharmacology Workbench, to analyze temporal changes in gene expression among epigenetic regulators of DNA methylation and demethylation, and one-carbon metabolism genes in response to cancer drug treatment. We analyzed gene expression information from the NCI-60 cancer cell line panel after treatment with five antitumor agents, 5-azacytidine, doxorubicin, vorinostat, paclitaxel, and cisplatin. Each antitumor agent elicited concerted changes in gene expression of multiple pathway components across the cell lines. Expression changes of *FOLR2*, *SMUG1*, *GART*, *GADD45A*, *MBD1*, *MTR*, *MTHFD1*, and *CTH* were significantly correlated with chemosensitivity to some of the agents. Among many genes with concerted expression response to individual antitumor agents were genes encoding DNA methyltransferases *DNMT1*, *DNMT3A*, and *DNMT3B*, epigenetic and DNA repair factors *MGMT*, *GADD45A*, and *MBD1*, and one-carbon metabolism pathway members *MTHFD1*, *TYMS*, *DHFR*, *MTR*, *MAT2A*, *SLC19A1*, *ATIC*, and *GART*.

**Conclusions:**

These transcriptional changes are likely to influence vital cellular functions of DNA methylation and demethylation, cellular growth, DNA biosynthesis, and DNA repair, and some of them may contribute to cytotoxic and apoptotic action of the drugs. This concerted molecular response was observed in a time-dependent manner, which may provide future guidelines for temporal selection of genetic drug targets for combination drug therapy treatment regimens.

**Electronic supplementary material:**

The online version of this article (doi:10.1186/s13148-016-0240-3) contains supplementary material, which is available to authorized users.

## Background

Epigenetic dysregulation is common in cancer and it involves, among other mechanisms, aberrant patterns of gene-specific DNA methylation, DNA hydroxymethylation, and genome-wide hypomethylation [1]. Hypermethylation of promoter regions of tumor suppressor genes in malignant cells leads to their epigenetic silencing [1–4]. Abnormal DNA methylation patterns such as DNA hypomethylation also increase genome instability [2, 5].

Levels and locus-specific patterns of DNA methylation are affected by a complex network of interactions among molecular factors (Additional file 1: Table S1). They include proteins directly involved in DNA methylation, e.g., DNA 5′ cytosine-methyltransferases or DNMTs (DNMT1, DNMT3A, and DNMT3B) and DNA hydroxymethylation and demethylation, such as ten-eleven translocation methylcytosine dioxygenases (TET1, TET2, and TET3), activation-induced cytidine deaminase (AICDA or AID), apolipoprotein B mRNA editing activity DNA deaminases (APOBEC1, APOBEC2, APOBEC3A, and APOBEC3C), thymine-DNA glycosylase (TDG), and demethylating DNA repair factors (O^6^-methylguanine-DNA-methyltransferase, or MGMT, and growth arrest and DNA damage 45 protein A, or GADD45A) [6–8]. Additional molecular factors include methyl-CpG-binding domain proteins (MBDs), proliferating cell nuclear antigen (PCNA), herpes virus-associated ubiquitin specific protease USP7, single-strand-selective monofunctional uracil-DNA glycosylase (SMUG1), and DNA methyltransferase 3-like protein (DNMT3L) that act as interaction partners of proteins involved in DNA methylation or demethylation, and NADP^+^-dependent isocitrate dehydrogenases (IDH1 and IDH2) that produce metabolites which interfere with TET-mediated DNA demethylation [6, 9–13].

DNA methylation processes are also affected by reactions in the folate-mediated one-carbon metabolism (OCM) pathway. This pathway encompasses a complex metabolic network of biosynthetic reactions in the cytoplasm, mitochondria, and the nucleus that involve *S*-adenosylmethionine (SAM or AdoMet), homocysteine (Hcy), folate, other B vitamins, and multiple cofactors [14–18]. The OCM pathway directly affects the activity of DNA methyltransferases and other methylation processes in the cell because reactions in that pathway involve the biosynthesis of SAM, which serves as a donor of methyl groups for DNA and other biological molecules. Furthermore, transmethylation reactions that use SAM as a substrate result in the conversion of SAM to *S*-adenosylhomocysteine (SAH), and SAH directly inhibits DNA methyltransferases [16, 17]. In addition to its influence on DNA methylation, the OCM pathway is involved in other vital cell functions that include purine and pyrimidine biosynthesis, amino acid biosynthesis, and cell growth and proliferation, all of which are highly important for rapidly proliferating cancer cells [19]. The OCM pathway involves multiple receptors, membrane transport proteins, and numerous important regulatory enzymes that control its reactions (Additional file 1: Table S1) [15, 17]. Genetic variation and expression of the OCM genes and abnormal levels of folate and Hcy have been associated with an increased risk of cancer, changes in drug transport, response to drug treatment, DNA methylation changes, DNA damage, and genome instability [14–17, 20–26].

Molecular components involved in DNA methylation, demethylation, and the OCM pathway have been targeted in cancer treatment through the use of hypomethylating agents (e.g., 5-azacytidine, or AZA, 2′-deoxy-5-azacitidine, or decitabine, other DNA methyltransferase inhibitors, and agents targeting other components of the methylation machinery) and antifolate drugs (e.g., methotrexate, 5-fluorouracil, aminopterin, pemetrexed, and other agents) [1, 2, 14, 27–31]. Treatment of tumor cells with low concentrations of 5-azacytidine results in global epigenome-wide demethylation, which leads to transcriptional reactivation of tumor suppressor genes that had been silenced by methylation [2]. At high concentrations, treatment with 5-azacytidine results in direct cytotoxicity rather than DNA hypomethylation [1]. Molecular mechanisms of action of 5-azacytidine include its incorporation into RNA and DNA, trapping of DNMT1, additional replication-independent mechanisms of DNMT1 depletion, and inhibition of ribonucleotide reductase, all of which leads to inhibition of transcription and of protein metabolism, and to induction of apoptosis [1, 2]. In the HCT-116 cancer cell line, 5-azacytidine downregulates the expression of DNA methyltransferases *DNMT1* and *DNMT3A* [32].

In addition to hypomethylating agents such as 5-azacytidine and decitabine, other cancer drugs with diverse mechanisms of actions can downregulate DNA methyltransferases and affect methylation status of a variety of genes. One of these agents is doxorubicin (Dox), an anthracycline antibiotic that inhibits topoisomerase II, generates reactive oxygen species (ROS), and causes CRB3L1-mediated membrane proteolysis [33]. In the HCT-116 cell line, doxorubicin downregulates the expression of DNMT1 and diminishes its enzymatic activity, leading to conditional apoptosis [34]. These changes in transcription and activity of DNMT1 in response to doxorubicin treatment did not lead to global DNA hypomethylation in a cell line model [34], but in live murine models, treatment with doxorubicin alone or in combination with Pluronic block copolymers resulted in the increase and decrease of methylation levels of numerous promoters of biologically important genes [35].

A histone deacetylase (HDAC) inhibitor, vorinostat (suberoylanilide hydroxamic acid, or SAHA) also affects DNA methylation. Treatment of tumor cells with vorinostat has been shown to downregulate transcription of DNA methyltransferases *DNMT1* and *DNMT3b* in the A549 lung cancer line and to induce methylation changes in important cancer-related genes such as human telomerase reverse transcriptase (*TERT,* or *hTERT*) and “deleted in liver cancer” (*DLC1*) [36–38].

DNA methylation and demethylation are not only involved in the mechanism of action of some cancer drugs but they have also been associated with sensitivity to drug treatment. One example of such involvement is MGMT, which participates in DNA repair by demethylating O^6^-methylguanine lesions. It also removes larger O^6^-alkyl adducts and is thereby involved in resistance to nitrosourea-based anticancer drugs [39]. Hypermethylation of the *MGMT* promoter leads to transcriptional repression of this gene, increasing cancer cell sensitivity to chemotherapeutic agents and radiation [39–41].

Epigenetic mechanisms are also involved in resistance to cisplatin, a small-molecule platinum compound that interacts with DNA to form DNA adducts and activates the apoptotic pathway [42]. Chemoresistance to cisplatin, which can develop after an initial positive response to treatment, has been associated with specific patterns of DNA methylation and gene expression, along a complex variety of other molecular changes [26, 41, 43–46].

Methylation of specific genes has been reported to affect tumor cell sensitivity and resistance to paclitaxel (Taxol®), a widely used cancer drug that binds to microtubules, disrupting their physiological assembly and disassembly, and leading to cell cycle arrest and apoptosis. At low concentrations, paclitaxel also has antiangiogenic properties and inhibits tumor vasculogenesis [47, 48]. Resistance to paclitaxel and progression-free survival may be associated with DNA methylation status of certain genes such as *SFN* (stratifin) and *CHFR* (checkpoint with forkhead and ring finger domains) [41, 49, 50].

Given the ubiquitous action of DNA methyltransferases and other epigenetic factors that participate in methylation and demethylation of DNA, and the link between DNA methylation and the OCM pathway, it is important to understand temporal patterns of the response of components of these pathways to drug treatment. Such knowledge is needed to gain an insight into molecular mechanisms of drug sensitivity and acquired drug resistance, and to identify suitable molecular drug targets. To address these questions, we investigated time-dependent expression patterns following treatment of cancer cell lines with several anticancer drugs with diverse mechanisms of action. We analyzed data from the NCI-60 cancer cell line panel, a well characterized and widely used resource for cancer drug screening and molecular biology studies of cancer [31, 51]. The time-course gene expression information was used to examine the effects of five widely used drugs: 5-azacytidine, vorinostat, doxorubicin, cisplatin, and paclitaxel, on the expression of multiple genes involved in DNA methylation, demethylation, and one-carbon metabolism. Two of these agents, 5-azacytidine and vorinostat, directly involve epigenetic mechanisms in their action, whereas doxorubicin has been reported to affect the expression of DNA methyltransferases in addition to other modes of action. For cisplatin and paclitaxel, epigenetic mechanisms involving DNA methylation have been associated with resistance to treatment. The goal of our analysis was to identify common patterns of expression changes in DNA methylation, demethylation, and OCM pathways among the different cell lines in response to each of these agents. Such common patterns may provide new knowledge about molecular mechanisms of drug action and could suggest potential novel gene targets for drug combination therapies. We also examined whether changes in the expression of these genes were associated with chemosensitivity to drug treatment.

## Results and discussion

**Fig. 1:**
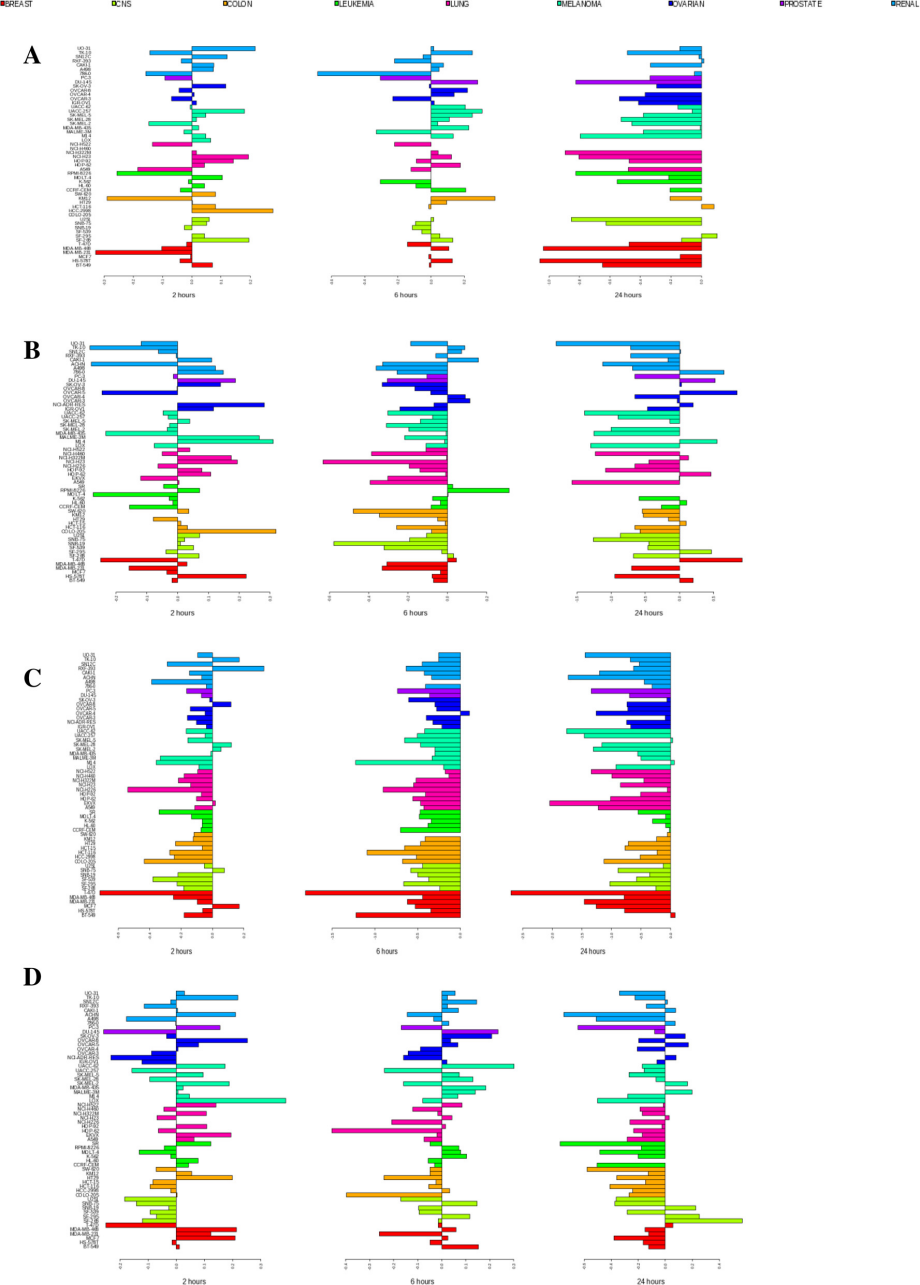
Changes in the expression of the *DNMT1* methyltransferase gene in the NCI-60 cell line panel. Shown are transcriptional changes of the *DNMT1* gene at 2 (*left panel*), 6 (*middle panel*), and 24 h (*right panel*) after treatment with high concentrations of **a** 5-azacytidine (5000 nM), **b** doxorubicin (1000 nM), **c** vorinostat (5000 nM), and **d** paclitaxel (100 nM). *Horizontal right bars* indicate elevated gene expression, whereas *left bars* indicate decreased expression relative to cell lines untreated by the drug. *Colors* represent types of cancer tissues (breast, central nervous system (CNS), colon, leukemia, lung, melanoma, ovarian, prostate, and renal cancers). The scale on the bottom represents log_2_ difference between expression values of treated and untreated cell lines. The scale for each microarray experiment is specific to that experiment

**Fig. 2:**
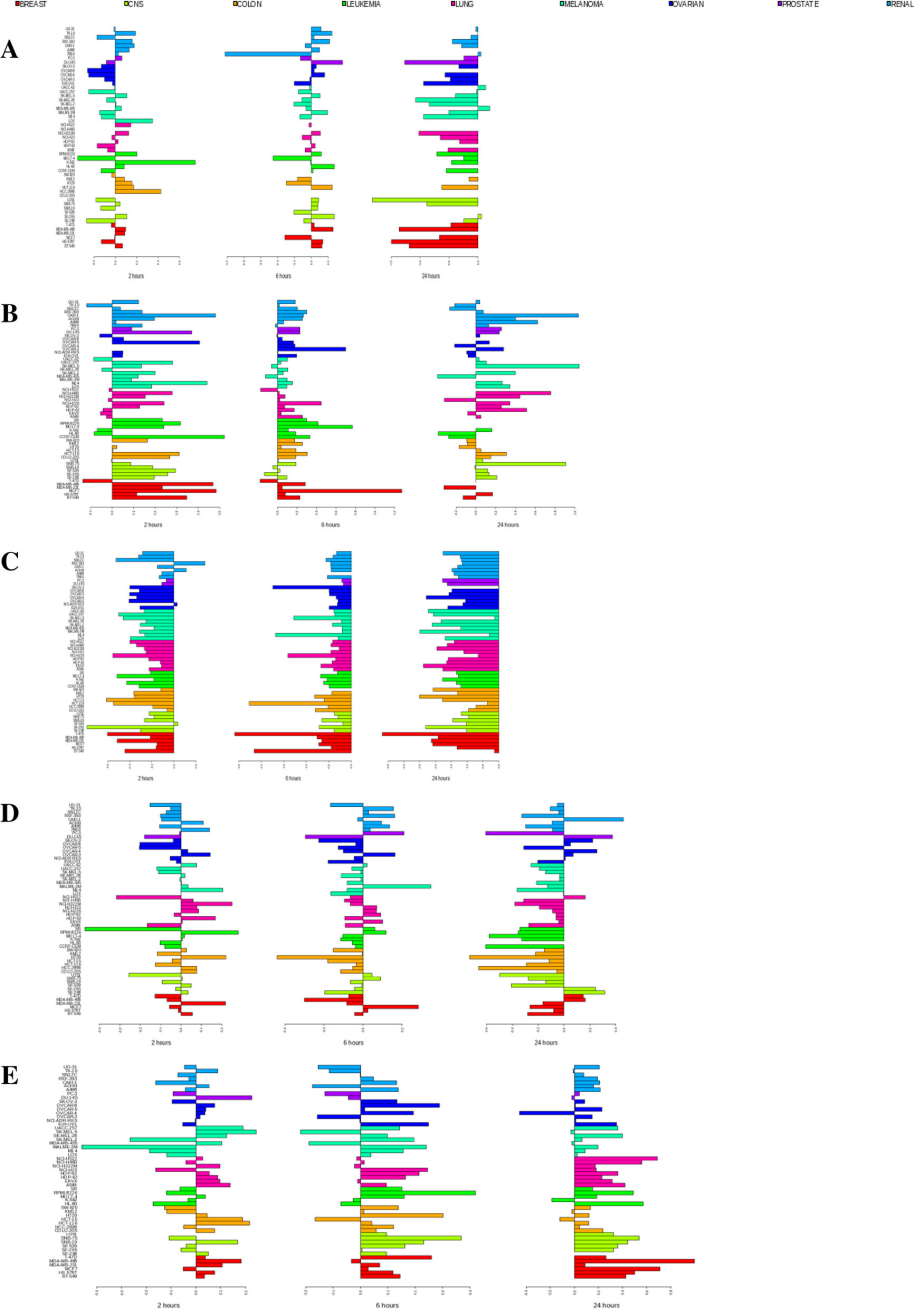
Drug-specific response patterns of changes in the expression of the *TYMS* gene. Shown are changes in the expression of the *TYMS* gene at 2 (*left panel*), 6 (*middle panel*), and 24 h (*right panel*) after treatment with high concentrations of **a** 5-azacytidine (5000 nM), **b** doxorubicin (1000 nM), **c** vorinostat (5000 nM), **d** paclitaxel (100 nM), and **e** cisplatin (15,000 nM). Additional information is provided in the legend to Fig. 1

The summary of gene expression changes in response to treatment with five antitumor agents is presented in Table 1. These results in Table 1 show concerted changes in the expression of many important components of the DNA methylation pathway, molecular factors involved in DNA demethylation, and enzymes, receptors, and transport proteins involved in the folate-mediated OCM pathway. Many genes with biologically important roles had concerted changes in expression, including multiple genes with a very strong similar response across all cancer types in the NCI-60 panel. In the majority of the experiments, higher concentrations of antitumor agents resulted in concerted changes in the expression of a higher number of cell lines and stronger amplitudes of expression changes than did the lower concentrations of the same agents. The most frequent time period when concerted changes were observed across multiple cell lines was at 24 h after treatment with cancer drugs. However, some individual genes had more profound concerted changes at 2 or 6 h after treatment (Table 1; Figs. 1 and 2; Additional file 2: Figures S2, S7, S8, S10-S12, S14, S16, S17). The concerted changes in expression were specific to individual agents, time points, and treatment concentrations. As discussed below, these concerted expression changes of epigenetic components and OCM genes uniquely identify four of the agents, 5′azacytidine, doxorubicin, vorinostat, and paclitaxel, or similar agents from the same classes, in independent datasets of transcriptional response to drug treatment. These changes identify elements of the DNA methylation, demethylation, and OCM pathways as differentially activated and inactivated based on mechanism of action, but the concerted nature of these responses indicate that they are overall independent of the cancer type in which the drug activity is tested. These concerted changes may therefore provide a basis for using potential drug combinations in a spectrum of tumors.

**Table 1:** Summary of time-specific drug treatment effects on gene expression in the NCI-60 cell lines

Gene	5-Azacytidine	Doxorubicin	Vorinostat	Paclitaxel	Cisplatin
*DNMT1*	H↓24^a^; L↓24	H↓6, 24^a^	HL↓2; H↓6^a^,24^b^; L↓6,24^a^	H↓24	NC
*DNMT3A*	NC	NC	H↓6^a^,24^a^; L↓6	NC	NC
*DNMT3B*	NC	H↓6^a^, 24^a^	H↑2^a^,6^b^; L↑2,6^a^	NC	NC
*DNMT3L*	NC	NC	NC	NC	NC
*TET3*	H↑24^a^	NC	L↓6^a^; HL↓24^a^	NC	NC
*AICDA*	NC	NC	NC	NC	NC
*APOBEC1*	NC	NC	NC	NC	NC
*APOBEC2*	NC	NC	NC	NC	NC
*APOBEC3A*	NC	NC	NC	NC	NC
*APOBEC3C*	H↓24^a^	L↑24^a^	H↓6^a^; HL↓24^a^	NC	H↑24^a^
*TDG*	H↑6,24^a^	NC	H↓2,6,24^a^; ↓6,24	H↑24	H↑24
*GADD45A*	H↑2^b^; HL↑6^a^,24^b^	H↑24^c^; L↑24^b^	H↑24^b^; L↑24^a^	HL↑24^b^	H↑6^a^,24^bd^; L↑24^ad^
*IDH1*	NC	H↑2,6; L↓24^b^	H↑2^a^,6^a^,24^a^; L↑2,6^a^,24^a^	L↓6; HL↓24^a^	H↓24^a^
*IDH2*	H↓24^a^	H↑6,24^a^; L↑24	NC	HL↓24	L↑24
*MGMT*	H↓24^a^; L↓24	H↓24^a^	NC	NC	H↓24^b^
*MBD1*	H↑24^a^; L↑24	H↑6,24^a^; L↑24	H↑6^a^,24^a^	HL↑24	H↑24^ad^
*MBD2*	H↓24	HL↓24	H↓24	NC	NC
*MBD3*	NC	NC	HL↓6^a^,24^a^	NC	NC
*MBD4*	H↓2^a^HL↑6	HL↑2; H↑6,24^a^	HL↑2,6^a^	NC	H↑6,24^a^; L↑24
*MeCP2*	NC	H↓6,24	HL↓6^a^; H↓24^a^; L↓24;	NC	NC
*PCNA*	L↑6; HL↓24^a^	H↑2,6^a^; L↑6,24^a^	L↓6; HL↓24^a^	HL↓24	H↑6,24
*USP7*	HL↓6; H↑24	HL↓6,24^a^	H↓2,6^a^,24^a^; L↓6	NC	H↓24
*SMUG1*	H↓2; HL↓6; H↑24^a^; L↑24	H↑24^a^; L↑24^d^	HL↑24^a^	NC	NC
*MTHFR*	NC	NC	NC	NC	NC
*MTHFD1*	H↓24^a^; L↓24	H↓24^b^	H↓6^a^,24^a^; L↓24	HL↓24	NC^d^
*MTR*	NC	HL↓6; H↓24^a^	H↓6^a^,24^a^; L↓6	H↓24^a^; L↓24	H↓6,24^ad^
*MTRR*	H↑6^a^,24^a^	L↓24^a^	H↑6^a^; L↓24	NC	H↑6
*CBS*	H↑24^b^; L↑24^a^	NC	NC	HL↓24^a^	NC
*TCN2*	NC	NC	H↑24^b^	NC	NC
*SHMT1*	H↓24^a^	H↓24^a^	HL↓24	HL↓24	NC
*TYMS*	H↓24^a^; L↓24	H↑2,6^a^; L↑6	HL↓2; H↓6^b^,24^b^; L↓6^a^,24^a^	HL↓24	H↑24
*DHFR*	H↓24^b^; L↓24^a^	H↑2, 24^a^; HL↑6	HL↓24^b^	HL↓24	H↑6; L↑24
*BHMT*	NC	H↑24	NC	NC	NC
*CTH*	H↑6^b^; L↑6^a^; HL↑24^b^	H↑6^a^,24^b^	H↑2^a^,6^b^,24^b^; L↑6^a^,24^a^	NC	H↑6,24^bd^
*AHCY*	H↓24^a^; L↓24	HL↑6; L↑24	H↓24^a^; L↓2,6,24	HL↓24	H↑6
*ALDH1L1*	NC	NC	NC	NC	NC
*ATIC*	H↓6; HL↑24	H↓24^a^; L↓24	H↓6^a^,24^a^; L↓2,6,24	NC	NC
*GART*	HL↑24	H↓6^a^,24^a^; L↓24	H↓6^a^,24^a^; L↓6,24	HL↓24^d^	NC
*MTHFS*	NC	H↓24^a^	H↓2; HL↓6^a^; H↓24^b^; L↓24^b^	NC	NC
*FTCD*	H↓24	NC	H↑24^c^	NC	NC
*MAT1A*	NC	NC	NC	NC	NC
*MAT2A*	H↓6,24^a^	H↓6^a^,24^b^; L↓6,24	NC	NC	H↓24^a^
*MAT2B*	H↓24; L↓2	NC	H↑2^a^; L↑2; HL↑6	NC	NC
*NNMT*	NC	NC	H↓2^c^,6^b^; L↓2,6^a^	NC	NC
*PON1*	NC	NC	NC	NC	NC
*SLC19A1*	HL↓24	H↓6^a^; HL↓24^a^	H↓6^a^; L↓6; HL↓24^a^	L↓24	H↓24
*FOLR1*	NC	NC	H↑24^b^	NC	NC
*FOLR2*	NC	H↑24^ad^	NC	NC	NC
*FOLR3*	NC	NC	NC	NC	NC
*SHMT2*	H↑6; HL↑24^a^	H↑6; H↓24^a^	H↓24^a^; L↓24	HL↓24	H↑6
*AMT*	H↓6	HL↑24	H↑24^a^	NC	NC
*MTHFD2*	H↑6^a^; L↑6; HL↑24^a^	NC	HL↓24^a^	L↓24^a^	H↑6,24^a^
*MTHFD2L*	NC	H↓6^a^,24^b^; L↓24^a^	NC	NC	H↓24^a^; L↑2
*PEMT*	NC	HL↓24^a^	HL↓6^a^,24^a^	NC	H↓24^a^; L↓24
*FOLH1*	H↓24^a^	NC	NC	NC	NC
*ALDH2*	NC	H↑6; HL↑24^a^	HL↑6^a^; H↑24^a^	NC	NC

Information about gene roles is provided in Additional file 1: Table S1. Concerted changes in expression (↑, upregulated or ↓, downregulated) are shown for microarray experiments in which nearly all cell lines had a change in the same direction, with no more than 15 cell lines showing a change in the opposite direction. Expression changes are shown if observed for the high concentration only (*H*), low concentration only (*L*), or both high and low concentrations (*HL*) of each antitumor agent. The time when the change was observed is also indicated. The drug concentrations for each agent are listed in the “Methods”. *NC* (not concerted), indicates that either the criteria of concerted expression were not satisfied or that those criteria were not indicative of concerted expression due to a large amount of missing data for a specific gene in a given microarray experiment. For example, H↑2,6^a^; HL↓24 indicates that a gene was upregulated after treatment with the high concentration of the drug at 2 and 6 h after treatment, with the change of log_2_ expression values at 6 h in at least some cell lines being ≥1 or ≤−1, and that the same gene was downregulated at both high and low concentrations of the drug at 24 h after treatment

^a^Concerted expression change as described above and log_2_ FC (the difference of log_2_ expression values between treated and untreated cells) in that direction in some cell lines was ≥1 or ≤−1

^b^Concerted expression change and log_2_ FC in that direction in some cell lines was ≥2.5 or ≤−2.5

^c^Concerted expression change as described above and log_2_ FC in that direction in some cell lines was ≥4 or ≤−4

^d^For at least one drug concentration and at least one time point, changes in expression were significantly correlated with log(GI50) as shown in Table 2

### Concerted patterns of expression changes in response to multiple agents

A number of genes with important biological roles had consistent concerted changes in expression after treatment by different antitumor agents. *DNMT1*, the maintenance DNA methyltransferase, was downregulated after treatment with 5-azacytidinine, doxorubicin, vorinostat, and paclitaxel (Table 1; Fig. 1), suggesting that cellular response to these drugs may involve possible reduction in DNA methylation mediated by DNMT1. *MGMT* expression was diminished after treatment with 5-azacytidinine, doxorubicin, and cisplatin. MGMT is involved in DNA demethylation and repair and in cellular resistance to cancer drug treatment (Additional file 1: Table S1) [40, 52]. Downregulation of *MGMT* decreases the ability of its product to repair DNA lesions after treatment with antitumor agents, leading to cancer cell death [40]. In contrast, *GADD45A* was consistently upregulated after treatment with each of the five drugs (Table 1; Additional file 2: Figures S2 and S7). *GADD45A* encodes a DNA repair-mediated DNA demethylating factor that can reactivate genes which had been silenced by methylation, and it is involved in apoptosis [8, 32, 53, 54].

Genes encoding methyl-CpG binding proteins (MBD1, MBD2, MBD3, MBD4, and MeCP2) had different responses to antitumor agents (Table 1; Additional file 2: Figures S2, S7 and S11). *MBD1* was consistently upregulated after treatment with each of the five drugs. *MBD2*, *MBD3*, and *MeCP2* were each downregulated by between one and three antitumor agents that include 5-azacytidine, doxorubicin, and vorinostat. Expression changes of *MBD4* were time- and drug-specific, with some treatment conditions resulting in its upregulation and others in downregulation. Products of these genes have different binding modes to methylated and unmethylated DNA, and they play different roles in transcriptional activation, transcriptional repression, DNA repair, and DNA demethylation (Additional file 1: Table S1) [1, 4, 10, 55–57]. Consistent upregulation of *MBD1* after treatment by each of the five drugs and downregulation of *MBD2*, *MBD3*, and *MeCP2* after treatment by certain antitumor agents demonstrate their different biological roles in epigenetic response to cancer treatment.

*SLC19A1*, the gene for reduced folate carrier (RFC) in the OCM pathway, was consistently downregulated in response to each of the five agents (Table 1). It encodes a major folate transporter across cell membrane, which is an important target for antifolate drugs [14, 15, 27, 29, 58, 59]. Its downregulation suggests that each of the anticancer drugs used in this study may have additional cellular effects by affecting the folate-mediated OCM pathway.

Several other OCM genes were downregulated in response to multiple agents (Table 1; Additional file 2: Figures S1-S2, S5, S8, S9, S12, S13, and S15). For example, the expression of *MAT2A* was diminished after treatment with 5-azacytidine, doxorubicin, and cisplatin. Its product, *L*-methionine *S*-adenosyltransferase IIα, catalyzes biosynthesis of SAM, the major source of methyl groups for methylation reactions (Additional file 1: Table S1) [60], and inhibition of MAT2A inhibits the production of SAM and suppresses methylation processes [61]. *MTHFD1*, which encodes methylenetetrahydrafolate dehydrogenase 1, was downregulated by 5-azacytidinine, doxorubicin, vorinostat, and paclitaxel, whereas *MTR*, the gene for methionine synthase, was downregulated by doxorubicin, vorinostat, paclitaxel, and cisplatin.

*CTH* was strongly upregulated after treatment with 5-azacytidinine, doxorubicin, vorinostat, and cisplatin (Additional file 2: Figure S3). Such upregulation is consistent with the role of its protein, cystathionase, in irreversible degradation of cystathionine, which is derived from Hcy, to cysteine. This transsulfuration pathway removes Hcy from the remethylation cycle and makes it unavailable for methylation reactions [15, 62, 63].

For some other genes in the OCM pathway, the direction of concerted expression changes was specific to individual agents (Table 1). For example, the expression of *GART*, which is involved in purine biosynthesis (Additional file 1: Table S1), was increased after treatment with 5-azacytidine but diminished after treatment with doxorubicin, vorinostat, and paclitaxel. *TYMS* and *DHFR*, which encode important antifolate drug targets thymidylate synthase and dihydrofolate reductase, respectively, were downregulated in a concerted manner after treatment by 5-azacytidinine, vorinostat, and paclitaxel, but upregulated after treatment with doxorubicin and cisplatin (Table 1; Fig. 2; Additional file 2: Figure S4). TYMS catalyzes the conversion of deoxyuridine monophosphate (dUMP) into deoxythymidine monophosphate (dTMP), which serves as a precursor for DNA synthesis and is used DNA repair (Additional file 1: Table S1). This reaction produces dihydrofolate (DHF), the reduction of which to THF is catalyzed by DHFR [18, 64]. Downregulation of several OCM genes including *TYMS* was previously noted after treatment of patient samples and cell lines of childhood B-lymphoblastic leukemia and of colon cancer cell lines with vorinostat [65, 66]. Furthermore, upregulation of *TYMS* and *DHFR* was a part of a gene expression signature that has been associated with relapse of acute lymphoblastic leukemia, and reversal by vorinostat of expression pattern of signature genes including *TYMS* and *DHFR* restored B-cell chemosensitivity to treatment [65]. Similarly, downregulation of *TYMS* by vorinostat has been critical in increasing the sensitivity of colorectal cancer cell lines to antifolate drugs and for overcoming chemoresistance [67]. Our results demonstrate that in addition to vorinostat, other anticancer drugs analyzed in this report also affect important reactions in the OCM pathway (Table 1), which may affect cytotoxic effects of these agents. Upregulation of *TYMS* and *DHFR* after treatment with doxorubicin and cisplatin suggests that it may be beneficial to target these genes in combination therapy treatments. Vorinostat and doxorubicin have synergistic cytotoxic action [68], and it could be postulated that downregulation of *TYMS* and *DHFR* by vorinostat may contribute to synergy between these drugs.

Interestingly, while DGIdb and PharmGKB reported interactions with paclitaxel and cisplatin for *MTHFR* encoding 5, 10-methylenetetrahydrofolate reductase that catalyzes one of the central OCM pathway reactions (Additional file 1: Table S1) [14, 18], its expression did not satisfy the criteria for concerted changes after treatment with any of the five agents (Table 1), although it showed a trend for upregulation at 2 h after treatment with the high concentration of paclitaxel (data not shown).

Of the genes listed in Table 1, *NNMT*, the gene for nicotinamide *N*-methyltransferase, demonstrated the largest amplitude of expression changes after treatment with 5-azacytidine (log_2_ FC <−6 and log_2_ FC >7 for some cell lines, where FC is fold change), doxorubicin (log_2_ FC reaching >5), and vorinostat (log_2_ FC <−7 and log_2_ FC >6 for some cell lines). Among the five agents, *NNMT* had concerted changes in expression only after treatment with the high concentration of vorinostat (Table 1), and the magnitude of its expression changes did not correlate with the direction of response to any of the agents or with chemosensitivity. Overexpression of *NNMT* in cancer cells reduces the cell methylation potential and leads to histone hypomethylation [69, 70]. The biological significance of the very strong expression response of *NNMT* to cancer drug treatment remains to be elucidated.

### Downregulation of DNMT1 and concerted changes in the expression of multiple other epigenetic components and OCM genes in response to 5-azacytidine

5-Azacytidine exerts its hypomethylating action by directly targeting the maintenance DNA methyltransferase DNMT1, depleting its amount in the cell by both covalent binding and by DNA replication-independent mechanisms [1, 71]. In the NCI-60 cell lines, the *DNMT1* gene was downregulated at 24 h at both high (Fig. 1a) and low concentrations of 5-azacytidine, whereas neither *DNMT3A* nor *DNMT3B* showed concerted changes in expression (Table 1). The observed downregulation of *DNMT1*, the lack of concerted changes in the expression of *DNMT3B*, and transcriptional activation of the apoptotic pathway were in agreement with earlier reports [1, 32, 53]. At 24 h, we did not observe downregulation of *DNMT3A* in the HCT-116 cell line after treatment with the low concentration (log_2_ FC = 0.228), in contrast to an earlier study [32], although we observed a minimal downregulation (log_2_ FC = −0.197) of *DNMT3A* in that cell line after treatment with the high concentration of 5-azacytidine.

Of the three TET family dioxygenases that convert 5-methylcytosine (5-mC) to 5-hydroxymethylcytosine (5-hmC), 5-formylcytosine (5-fC), and 5-carboxylcytosine (5-caC) [1, 6], expression data were available for *TET3*, which was upregulated at 24 h after treatment with the high concentration (Table 1; Additional file 2: Figure S1). *TDG*, another component of the DNA demethylation pathway, was also upregulated (Additional file 2: Figure S2). This suggests that upregulation of *TET3* and *TDG* may increase the rate of DNA demethylation and contribute to the demethylating action of 5-azacytidine.

It was previously suggested [52] that hypomethylating agents targeting DNA methyltransferases may restore the activity of MGMT and thereby increase antidrug resistance. The data in Table 1 show that *MGMT* was downregulated at 24 h, indicating at least an initial decrease in *MGMT* transcription. *GADD45A* was strongly upregulated (Additional file 2: Figures S1 and S2), in agreement with its role in mediating TP53-induced apoptosis [32, 53] and its possible synergistic contribution to the demethylating action of 5-azacytidine [54]. Strong upregulation of *GADD45A* was previously reported in the Molecular Signatures Database (MSigDB) as part of molecular signature characteristic of breast cancer cell line response to 5-azacytidine [72] via demethylation of *GADD45A* promoter after treatment [73].

The expression of *APOBEC3C* was diminished at 24 h after treatment. APOBEC3C participates in DNA demethylation by reducing the levels of 5hmC, but members of the APOBEC family are also involved in DNA repair (Additional file 1: Table S1) [74]. Therefore, it may be possible that downregulation of *APOBEC3C* may contribute to cell death through diminished DNA repair.

After treatment with 5-azacytidine, *MBD1* was upregulated, *MBD2* was downregulated, while expression changes of *MBD4* were time-dependent (Table 1; Additional file 2: Figures S1 and S2). The differences in their expression changes are likely related to their different biological roles. Expression patterns of several other epigenetic factors including *PCNA*, *USP7*, and *SMUG1*, which may affect DNA methylation, DNA demethylation, and DNA repair (Additional file 1: Table S1) [1, 6, 11, 74–76], changed with time (Table 1; Additional file 2: Figure S2). While *IDH1* expression did not satisfy the criteria for concerted changes, *IDH2* was downregulated at 24 h after treatment with the high concentration of 5-azacytidine, suggesting differences in their regulation. These changes illustrate the dynamic nature and complexity of regulation of epigenetic changes and DNA repair and replication following drug treatment.

Many important OCM genes including *TYMS*, *DHFR*, *AHCY*, *FTCD*, *MAT2A*, *MAT2B*, and *AMT* were downregulated after treatment with 5-azacytidine (Table 1; Fig. 2, Additional file 2: Figures S1, S2, and S4). *TYMS* and *DHFR* are important drug targets [27], and their downregulation is important for cytotoxicity of cancer drug treatment [65, 67]. MAT2A and MAT2B, the components of the *L*-methionine *S*-adenosyltransferase II, and AHCY, the *S*-adenosyl-*L*-homocysteine hydrolase, are involved in the control of the levels of SAM and SAH, which are important for methylation reactions [60, 61, 63, 77, 78]. *FOLH1*, which is involved in intestinal absorption of dietary folate and in its conversion to folate, affecting folate levels in vivo [79], was also downregulated. Hypermethylation of the *FOLH1* promoter has been associated with the risk of relapse and poor clinical outcomes of childhood neuroblastoma [80]. The role of *FOLH1* in in vitro cellular response requires further elucidation.

*SLC19A1*, which is involved in folate transport, was downregulated, whereas none of the folate receptor genes involved in folate endocytosis satisfied the criteria for concerted expression changes. These results are in agreement with earlier studies [73, 81], which demonstrated the effect of demethylating agents 5-azacytidine and decitabine on transcription, promoter methylation, and protein expression of *SLC19A1* but not of the folate receptor genes*.*

Among the upregulated OCM genes were *CBS* and *CTH*, which participate in the conversion of Hcy to cysteine, removing Hcy from the methylation cycle [15], *MTRR*, and *GART* (Table 1; Additional file 1: Table S1; Additional file 2: Figures S1 and S3). *GART* is an element of the purine biosynthetic pathway, and its increased expression may predict poor outcome in glioma and hepatocellular carcinoma [82, 83]. This suggests that *GART* may be examined as a potential target in drug combinations that include 5-azacytidine, in order to enhance the treatment effect by using an additional agent that would suppress purine biosynthesis (Additional file 1: Table S1). The changes in the expression of *ATIC*, which is also involved in purine biosynthesis, were time-dependent (Table 1). *SHMT2* and *MTHFD2*, the products of which are involved in the OCM pathway in mitochondria, were upregulated. In contrast, *SHMT1* and *MTHFD1*, the products of which regulate an analogous set of cytoplasmic reactions, were downregulated (Table 1; Additional file 1: Table S1) [18, 84]. This indicates compartmentalization of folate metabolism in response to this drug.

To examine the downstream cellular effects of expression changes in methylation machinery, we analyzed the expression changes of 91 potential target genes at 24 h after treatment with the high concentration of 5-azacytidine (Additional file 2: Figure S1), when the most profound expression changes were observed. In agreement with earlier reports [32, 53] that suggested links between the downregulation of DNMT1 by 5-azacytidine, TP53-mediated apoptosis, and changes in the expression of DNA damage response genes, we observed strong upregulation of *CDKN1A* (p21) which is involved in cell cycle arrest, and upregulation of *TP53* and *BAK1*, which are involved in apoptosis, although these early increases are likely to be in response to DNA damage [85]. We also observed changes in the expression of multiple other cancer-related genes (Additional file 2: Figure S1). Many upregulated genes that are involved in DNA damage response, DNA repair, maintenance of genome integrity, and cell growth arrest have been reported to be reactivated after treatment due to changes in their methylation status [53, 65, 86]. While gene re-expression induced by DNA demethylation is generally believed to take 3–7 days, some reports indicate that it can happen as early as at 12–24 hours after treatment [87–89]. Among genes induced by 5-azacytidine in some of the cell lines was *PTEN*, the expression of which is induced by demethylation of its promoter after treatment [90]. *RASSF1*, which is involved in cell cycle control and microtubule stabilization, was also moderately upregulated*.* As its isoform, *RASSF1A*, is silenced by DNA methylation in tumor cells [91–93], it is possible that *RASSF1* expression was reactivated through hypomethylation after treatment with 5-azacytidine and may contribute to improved outcome in response to treatment.

### Concerted changes in the expression of DNA methyltransferase genes, other epigenetic factors, and multiple OCM genes in response to doxorubicin

Treatment of the HCT-116 cell line with 1 μM of doxorubicin has been reported to inhibit DNMT1 activity and to cause conditional apoptosis [34]. In our dataset, at 6 and 24 h after treatment with 1 μM of doxorubicin, both *DNMT1* and *DNMT3B* genes were downregulated in the majority of the cell lines (Table 1; Fig. 1b; Additional file 2: Figure S6A). Therefore, doxorubicin both inhibits activity and downregulates these DNA methyltransferases, indicating robust inhibition of methylation. Interestingly, while *DNMT3A* did not satisfy the criterion for concerted expression changes, it showed a strong trend for upregulation in the majority of the cell lines at 24 h (data not shown), suggesting that it may be considered as a potential target for combination therapy with doxorubicin and an additional agent targeting this specific DNA methyltransferase.

*APOBEC3C*, *GADD45A*, and *IDH2*, which are involved in or generate products that interfere with DNA demethylation, were upregulated after treatment (Additional file 2: Figures S5 and S7). The response of *IDH1* was time-specific (Table 1), and the expression of *MGMT* was diminished. Among the genes encoding methyl-CpG-binding domain proteins, *MBD1* and *MBD4* were upregulated, and the expression of *MBD2* and *MeCP2* was diminished, underlying differences in their regulation and action (Table 1; Additional file 1: Table S1; Additional file 2: Figures S5 and S7). *USP7* was downregulated, whereas *PCNA* was upregulated. *SMUG1* was also upregulated, and the change in its expression was negatively correlated with chemosensitivity (Tables 1 and 2). *SMUG1* encodes a DNA demethylating and DNA repair agent that participates in the base excision repair (BER) pathway [1, 6, 74, 94] (Additional file 1: Table S1). Its upregulation indicates active BER pathway response to doxorubicin-induced DNA damage and suggests that targeting *SMUG1* in combination therapy might increase doxorubicin activity by increasing cell susceptibility to DNA damage.

**Table 2:** Candidate genes involved in DNA methylation, demethylation, and one-carbon metabolism, for which expression changes were significantly correlated with chemosensitivity or chemoresistance to drug agents

Gene	Antitumor agent	Drug concentration and time after treatment	*r*	FDR adjusted *p* value
*FOLR2*	Doxorubicin	High, 24 h	−0.509	0.0366
*SMUG1*	Doxorubicin	Low, 24 h	−0.488	0.0284
*GART*	Paclitaxel	Low, 24 h	0.462	0.0422
*GADD45A*	Cisplatin	High, 24 h	−0.618	0.0011
*GADD45A*	Cisplatin	Low, 24 h	−0.477	0.0422
*MBD1*	Cisplatin	High, 24 h	−0.570	0.0046
*MTR*	Cisplatin	High, 24 h	0.564	0.0046
*MTHFD1*	Cisplatin	High, 24 h	0.552	0.0061
*CTH*	Cisplatin	High, 24 h	−0.531	0.0122
*CTH*	Cisplatin	Low, 24 h	−0.504	0.0234

Listed are genes involved in DNA methylation, demethylation, and the OCM pathway which satisfied FDR adjusted *p* <0.05 for Pearson correlation of their expression changes with chemosensitivity

*r* Pearson coefficient of correlation of log_2_ FC with log(GI50) values across NCI-60 cell lines

Among the components of the OCM pathway, we observed strong concerted downregulation of *MTR*, *MTRR*, *MAT2A*, *MTHFD1*, *SHMT1*, and *MTHFS* (Table 1)*.* Also downregulated were *ATIC* and *GART*, regulators of purine biosynthesis, *MTHFD2L* which participates in the OCM pathway in mitochondria [18], and *PEMT*, the product of which catalyzes the de novo synthesis of phosphatidylcholine using SAM as methyl donor (Additional file 1: Table S1) [63].

*CTH*, *BHMT*, *AHCY*, *AMT*, *ALDH2*, and multiple other OCM genes were upregulated (Table 1; Additional file 1: Table S1). *BHMT* has been reported to have a protective role against cancer, and its absence promotes malignancies in the liver [64, 95]. Inhibition of *AHCY* has been associated with DNA hypomethylation and cancer progression, whereas its upregulation contributes to apoptosis and cell differentiation [63, 96]. While the function of *ALDH2* has been associated with folate levels in vivo [97], upregulation of this gene, in accord with our measurements, was found to be a part of an MSigDB molecular signature in mouse cell lines undergoing apoptosis in response to doxorubicin [72, 98]. Other upregulated genes included *TYMS* and *DHFR* (Table 1; Fig. 2; Additional file 2: Figures S4, S5 and S8), suggesting that they could be considered as targets in combination drug therapy with doxorubicin.

Although *ALDHD1L1* did not satisfy the criterion for a concerted change, it showed a trend for increased expression at 24 h after treatment with the high concentration (data not shown), in agreement with earlier reports [72, 98]. This may have clinical importance because its product is a major regulator of availability of folate-bound carbon groups for cellular processes, affecting the availability of methyl groups (Additional file 1: Table S1) [17, 99]. *ALDH1L1* is epigenetically silenced in cancers, and its upregulation induces cytotoxicity and apoptosis [17, 99], suggesting that its increased expression in specific cell lines after treatment may contribute to the antitumor action of doxorubicin.

The expression of *FOLR2* was increased at 24 h after treatment with the high concentration of doxorubicin, and this increase was negatively correlated with chemosensitivity (Table 2). The folate transporter gene, *SLC19A1*, was downregulated, whereas neither *FOLR1* nor *FOLR3* receptor genes showed concerted expression changes. These results are in agreement with earlier studies that suggested the distinctive role of FOLR2 in the cytotoxicity of folate-targeted doxorubicin [100]. Additionally, multiple expression changes listed in Table 1 in response to doxorubicin confirm earlier reports that downregulation of *MBD2*, *MECP2*, and *SLC19A1* and upregulation of *GADD45* and *ALDH2* were a part of an MSigDB molecular signature in mouse cell lines undergoing apoptosis in response to the high concentration (1000 nM) of doxorubicin [72, 98].

When potential target genes of epigenetic regulation were examined, at 24 h after treatment with the high concentration of doxorubicin, we observed an upregulation of a number of tumor suppressor genes and other genes involved in DNA replication and repair, cell growth arrest, and inhibition of cell proliferation (Additional file 2: Figure S5). Changes in the expression of *DDB2* and *CDKN1A* correlated with chemosensitivity to the low concentration of doxorubicin at 24 h (*r =* −0.568, FDR corrected *p =* 0.0046 for *DDB2* and *r* = −0.489, *p* = 0.0284 for *CDKN1A*). An earlier study showed that the expression of *DDB2*, which is involved in response to DNA damage, is induced by doxorubicin, and the level of that response and cell sensitivity to doxorubicin were regulated by BRCA1 [101]. In our study, transcriptional changes of *BRCA1* were only weakly correlated with chemosensitivity to doxorubicin (*r* between −0.335 and 0.254), but the inverse correlation of expression changes of its target, *DDB2* with log(GI50) confirmed the importance of the DNA damage response pathway in chemoresistance to this agent. It remains to be determined whether these expression changes were induced through direct transcriptional regulatory response to DNA damage or via epigenetic regulatory mechanisms.

Hypermethylation of *GSTP1* and *ABCB1* has been suggested to predict chemosensitivity to doxorubicin [41]. *GSTP1* was upregulated at 6 and 24 h after treatment with the high concentration and at 24 h after treatment with the low concentration of the agent, whereas *ABCB1* had variable patterns of expression among cell lines. However, correlation of expression changes of both *GSTP1* and *ABCB1* with chemosensitivity was weak (*r* between −0.3 and 0.35).

Treatment of the HCT-116 cell line with high concentrations of doxorubicin inhibits DNMT1 and leads to apoptosis [34]. Induction of apoptosis by doxorubicin occurs via a TP53-dependent mechanism [102], consistent with upregulation of *TP53* in our study. Because treatment with doxorubicin leads to extensive changes in the expression of genes encoding DNA methyltransferases and genes involved in DNA demethylation, further studies are needed to examine whether doxorubicin-induced silencing and reactivation of tumor suppressor genes and genes involved in cell cycle control and DNA repair (Additional file 2: Figure S5) are modulated by DNA methylation processes or by other regulatory mechanisms that are independent from DNA methylation and demethylation.

The T-47D breast cancer cell line is resistant to doxorubicin [102]. We observed a distinct pattern of expression changes in the T-47D cell line in response to this agent, which was very different from all other NCI-60 cell lines (Additional file 2: Figures S5-S8), suggesting unique features in its biological response to doxorubicin.

### Strong concerted expression changes among components of epigenetic and folate-mediate OCM pathways in response to vorinostat

Vorinostat and other HDAC inhibitors lead to cell differentiation, inhibition of cell growth, apoptosis mediated by inhibition of DNMT1, and global and gene-specific DNA hypomethylation [36].

In agreement with earlier reports [36, 65, 66], treatment with vorinostat led to marked changes in the expression of many genes with biological roles outside the histone modification pathways. This included extensive consistent expression changes among components of DNA methylation and demethylation pathways and among members of the OCM pathway (Table 1; Figs. 1 and 2; Additional file 2: Figures S3C, S4C, S6B, and S9-S12).

We observed concerted downregulation of DNA methyltransferase genes *DNMT1*, which confirmed an earlier report [36], and *DNMT3A* (Table 1; Figs. 1c and 2a). Interestingly, *DNMT3B* was upregulated at 2 and 6 h after treatment (Additional file 2: Figure S6B). The biological implications of the short-term upregulation of *DNMT3B* in response to vorinostat require further investigation.

*GADD45A* was upregulated. Expression of several other genes involved in demethylation, including *TET3*, *APOBEC3C*, and *TDG*, was diminished (Table 1; Additional file 2: Figures S9 and S11). While it did not satisfy the criterion for a concerted change, *MGMT* showed a trend for downregulation at 24 h in the majority of the cell lines (data not shown). The diminished expression of *TDG*, *APOBEC3C*, and *MGMT* may increase susceptibility of cancer cells to treatment due to reduced DNA repair, as vorinostat has been shown to induce ROS and DNA damage [103]. Multiple other epigenetic components also demonstrated strong concerted expression changes (Table 1).

In agreement with previous studies [65, 66], we observed strong downregulation of multiple folate metabolism genes including *MTHFD1*, *MTR*, *SHMT1*, *TYMS*, *DHFR*, *AHCY*, *ATIC*, *GART*, *MTHFS*, *SLC19A1*, *PEMT*, *NNMT*, and components of mitochondrial pathways, *SHMT2* and *MTHFD2* (Table 1; Fig. 2c; Additional file 2: Figures S4C, S9 and S12). These changes suggest downregulation of SLC19A1-mediated folate transport and of reactions that result in nucleotide and DNA synthesis, DNA repair, biosynthesis of phosphatidylcholine, or provide methyl groups for cellular methylation processes (Additional file 1: Table S1)*.* At 24 h, *TYMS* expression in many cell lines dropped to the levels of log_2_ FC <−2, with changes in the SF-295 cell line reaching log_2_ FC = −3.68. Downregulation of *TYMS*, *DHFR*, and *ATIC* is clinically important for the cytotoxic effects of cancer treatment [65, 67].

*TCN2* and *CTH* (Additional file 2: Figure S3C) were strongly upregulated after treatment with the high concentration of vorinostat (maximum log_2_ FC = 3.13 for *TCN2*, 3.69 for *CTH* at 24 h). The latter suggests the increase in removal of Hcy from methylation cycle. The expression of *FTCD* was also increased, with very strong upregulation (log_2_ FC >4) in several renal and ovarian cancer cell lines at 24 h after treatment with the high concentration. Among other upregulated genes were *MAT2B*, *AMT*, *ALDH2*, and *FOLR1* (Additional file 2: Figures S9 and S12). Changes in *MTRR* expression were time-dependent (Table 1).

Among potential methylation targets, at 24 h after treatment with the high concentration of vorinostat, we observed some level of upregulation of the tumor suppressor gene *DLC1* and moderate downregulation of *TERT* in most of the cell lines (Additional file 2: Figure S9), in agreement with their previously reported expression response to vorinostat due to methylation changes [36–38]. The expression of *TP53*, which had been suggested to have a synergistic effect with downregulation of *TYMS* in chemosensitivity to drug therapy [67], was strongly diminished in the majority of the cell lines at 6 and 24 h after treatment with high and low concentrations. The direction of change in abundance of the p53 protein in colorectal cancer cell lines has been reported to depend on vorinostat concentration and *TP53* mutation status [67]. However, p53 protein abundance and activity are regulated by acetylation [67, 68]. Therefore, mechanisms and consequences of strong transcriptional downregulation of *TP53* after treatment with vorinostat require further investigation. Multiple other cancer-related genes also showed strong changes in expression (Additional file 2: Figure S9). In addition to possible DNA methylation changes, their expression may be regulated by vorinostat via other mechanisms including histone acetylation and changes in miRNA expression [37].

### Concerted changes in expression of several components of DNA methylation machinery and downregulation of multiple components of the OCM pathway in response to paclitaxel

As a general trend after treatment with paclitaxel, transcriptional changes among components of DNA methylation and demethylation pathways and the OCM pathway were not as strong or as concerted as those after treatment with other antitumor agents investigated in this study (Table 1; Additional file 2: Figure S13). However, several genes demonstrated concerted transcriptional changes, including downregulation of *DNMT1*, *IDH1*, *IDH2*, and *PCNA* (Table 1; Fig. 1d; Additional file 2: Figures S13 and S14) and upregulation of DNA demethylating agents *TDG* and *GADD45A*, and of the methyl-CpG-domain binding protein 1, *MBD1*.

Expression of a number of important components of the folate-mediated OCM pathway was diminished, with the majority of downregulation observed at 24 h (Table 1). Among the downregulated genes were *MTHFD1*, *MTR*, *CBS*, *SHMT1*, *TYMS*, *DHFR*, *AHCY*, *GART*, *SLC19A1*, *SHMT2*, and *MTHFD2* (Fig. 2d; Additional file 2: Figures S4D, S13 and S14), suggesting that treatment with paclitaxel may inhibit reactions that involve folate intake, DNA synthesis, and synthesis of phosphatidylcholine (Additional file 1: Table S1)*.* Expression changes in *GART* were significantly correlated with chemosensitivity (Table 2), and as *GART* expression is associated with poor prognosis in several cancers [82, 83], this might indicate a particular requirement for purine biosynthesis in response to drug treatment. None of the OCM genes satisfied the criteria for concerted upregulation; however, several genes, most notably *MTHFS* at 2 h after treatment*,* demonstrated a trend for upregulation among the majority of the cell lines (data not shown).

Among the potential methylation targets, *RASSF1* was predominantly upregulated (Additional file 2: Figure S13). Its RASSF1A isoform has been reported to participate in microtubule stabilization with the effect similar to that of paclitaxel, and to be silenced by DNA hypermethylation, and both RASSF1A and RASSF1C isoforms act as tumor suppressors and contribute to cell death [93].

Methylation of the stratifin gene, *SFN*, has been associated with progression-free survival of ovarian cancer patients after treatment with paclitaxel-carboplatin versus docetaxel-carboplatin [49]. No concerted expression changes of *SFN* were observed in our study, and while there was some trend for its upregulation at 24 h after treatment with the high concentration of paclitaxel, transcriptional changes of *SFN* did not correlate with chemosensitivity (*r* between −0.188 and 0.088).

Four cell lines, DU-145, SF-295, CAKI-1, and SF-268, showed a trend for a reverse pattern of transcriptional response to paclitaxel when compared to the majority of other cell lines including PC3 (Additional file 2: Figure S13). This is consistent with a suggestion [104] that the differences between response of the DU-145 and PC3 cell lines to paclitaxel involve phosphorylation of the p16 protein in DU-145, which leads to apoptosis, whereas the PC3 cells undergo a rapid mitotic slippage and have a defective post-mitotic checkpoint. These observations and a weak transcriptional response among components of epigenetic machinery suggest that transcriptional changes of many cancer-related genes in response to paclitaxel are likely regulated by mechanisms other than DNA methylation or demethylation.

### Expression response of several epigenetic factors and OCM genes to cisplatin

In agreement with previous reports and molecular signatures in MSigDB [72, 105, 106], after treatment with cisplatin the expression of *GADD45A*, *APOBEC3C*, *TDG*, *IDH2*, *MBD4*, and *PCNA* was increased, and at 24 h, the changes in the *GADD45A* expression were significantly correlated with chemosensitivity (Tables 1 and 2; Additional file 2: Figure S16). The strongest upregulation of *GADD45A* was observed at 24 h after treatment with the high concentration, and in some cell lines, it reached log_2_ FC >3. *MBD1* was also upregulated at 24 h after treatment with the high concentration, which was significantly negatively correlated with chemosensitivity (Table 2). *MGMT* was strongly downregulated (in some cell lines, log_2_ FC <−2.5 at 24 h after treatment with the high concentration) (Table 1; Additional file 2: Figure S16), consistent with published data [107], even though MGMT is not involved in cisplatin adduct repair. *IDH1* and *USP7* were also downregulated.

The OCM genes *MTR*, *MAT2A*, *SLC19A1*, and *PEMT* were downregulated (Table 1), suggesting possible inhibition of folate intake and of reactions leading to methionine remethylation and biosynthesis of SAM and phosphatidylcholine. Expression of multiple other important OCM genes including *TYMS* and *DHFR* was increased (Table 1; Fig. 2e; Additional file 2: Figures S3D, S4E, S15 and S17). Increased expression of *TYMS* at 24 h after treatment with cisplatin is in agreement with an earlier report [106], and upregulation of *TYMS* and *DHFR* suggests that they could be examined as potential targets in combination therapy with cisplatin. The two mitochondrial isozyme genes, *MTHFD2* and *MTHD2L*, have different expression patterns that are specific to developmental stage and tissue differentiation [84], which may explain differences in their regulation in response to cisplatin and to other agents (Table 1). Changes in the expression of *MTHFD1*, *MTR*, and *CTH* were significantly correlated with chemosensitivity (Table 2), suggesting the importance of OCM reactions in cytotoxicity of cisplatin.

Treatment with cisplatin led to changes in the expression of multiple potential methylation target genes (Additional file 2: Figure S15), and at 24 h, transcriptional changes in many of those genes were significantly correlated with chemosensitivity. The majority of significant correlation was observed after treatment with the high concentration, including *PPP1R15A* (*r =* 0.700, FDR adjusted *p =* 1.24 × 10^−5^), *DDIT3* (*r =* −0.584, *p* = 0.0042), *CEBPG* (*r =* −0.567, *p* = 0.0046), *RASSF1* (*r =* −0.555, *p* = 0.0060), *RARB* (*r =* 0.535, *p* = 0.0113), *POLA1* (*r =* 0.517, *p* = 0.0181), *BTG2* (*r =* −0.507, *p =* 0.0234), and *ATXN3* (*r =* −0.499, *p* = 0.0284). Expression changes of *CEBPG* were also correlated with chemosensitivity to the low concentration of the agent (*r =* −0.520, *p* = 0.0148 at 24 h).

It remains to be investigated whether epigenetic modifications play a role in modulating this transcriptional response. For example, upregulation of *PPP1R15A* (*GADD34*), which is involved in cellular stress response, was highly significantly correlated with log(GI50). Increased expression of *PPP1R15A* has been linked to enhanced cytotoxicity of cisplatin [108, 109], but not to DNA methylation changes. In contrast, hypermethylation of *FANCF*, *SFN*, *MLH1*, and *TP73* has been associated with chemosensitivity to cisplatin [41]. In our study, *FANCF*, a DNA damage response gene, was upregulated at 2 h after treatment with the low concentration and downregulated at 24 h after treatment with the high concentration of cisplatin, but correlation of its expression changes with chemosensitivity was very weak (*r* between −0.2 and 0.2). Epigenetic silencing of *MLH1*, which is involved in DNA mismatch repair, has also been suggested to be among determinants of cisplatin resistance [41, 44]. *MLH1* was upregulated at 24 h after treatment with the low concentration of cisplatin, but correlation between its expression changes and chemosensitivity was weak (*r* between −0.307 and 0.222). *SFN* was upregulated at 6 and 24 h after treatment with the high concentration, but correlation of its expression changes with log(GI50) was only modest (*r* between −0.376 at 2 h and −0.387 at 24 h after treatment). No concerted expression changes or correlation with chemosensitivity were observed for *TP73*.

### Validation of patterns of gene expression changes in response to 5-azacytidine, doxorubicin, vorinostat, and paclitaxel in other datasets

The Connectivity Map (cmap) [110] utilizes gene expression measurements at 6 h after treatment with 5-azacytidine, doxorubicin, vorinostat, and paclitaxel at concentrations that exceed the highest concentration of these agents in the Transcriptional Pharmacology (TP) Workbench. At 6 h after treatment with paclitaxel, no genes in our study satisfied conditions of concerted up- and downregulation for the high concentration, and only one gene (*IDH1*) had a concerted change at the low concentration, and therefore, no comparable searches could be performed for that agent. When we analyzed the lists of up- and downregulated genes (Table 1) after treatment with 5-azacytidine, doxorubicin, and vorinostat, cmap permutation analysis confirmed the concerted expression changes for vorinostat that were observed in our study. When the list of genes with concerted changes at 6 h after treatment with the high concentration of vorinostat from Table 1 was used as input, cmap analysis identified vorinostat as the second highest ranking agent among 1309 agents in the cmap database (permutation-based *p* = 0, enrichment measure = 0.892). The third ranking agent was found to be another HDAC inhibitor, trichostatin A (*p* <10^−6^). Even though the connections and the ranking of cmap gene lists are based on the magnitude of expression response, whereas the concerted changes in our study were determined based on the high proportion of NCI-60 cell lines with expression changes in the same direction, the gene lists for vorinostat from both datasets were in strong agreement, due to consistent concerted changes among the cell lines and the strong magnitude of expression response to this agent (Table 1). Neither 5-azacytidine nor doxorubicin was among the top cmap hits when their respective up- and downregulated gene lists from Table 1 at 6 h after treatment with their high concentrations were investigated. The differences in ranking the response to 5-azacytidine and doxorubicin between the TP Workbench and cmap may be related to different concentrations of these agents in the two datasets, which could trigger different types of cellular response (e.g., demethylating action or apoptosis for different concentrations of 5-azacytidine [1]). These differences may also be related to the different methods for identifying up- and downregulated genes, as our gene lists were based on concerted changes among the NCI-60 cell lines, as opposed to the cmap ranking that used the magnitude of changes in a small number of individual cell lines. Despite these differences, the second best ranking agent in the Connectivity Map analysis of the gene signature composed of up- and downregulated genes at 6 h after treatment with 1000 nM of doxorubicin (Table 1) was another anthracycline antibiotic, daunorubicin [111] (*p* = 0, enrichment = 0.948). This similarity was based on cmap measures of transcriptional response at 6 h after treatment with 1000 and 7000 nM of daunorubicin. This suggests that cancer cell treatment with a lower concentration of doxorubicin (1000 nM) than that available in the cmap dataset for doxorubicin (6800 nM) elicits a response similar to that of the comparable concentration of daunorubicin.

Many experiments in the Library of Integrated Network-Based Cellular Signatures (LINCS) also correspond to higher concentrations of the cancer drugs examined in this study than those in the TP Workbench. Despite these differences, our searches of LINCS data confirmed the validity of the lists of genes with concerted changes identified in Table 1 for 5-azacytidine, doxorubicin, vorinostat, and paclitaxel. When we used median log_2_ FC values and the gene lists from Table 1, the LINCS L1000 Characteristic Direction Signature Search Engine (L1000CDS^2^) search for 6 h after treatment with the high concentration of 5-azacytidine returned LINCS experiments corresponding to 6 h after treatment with 10,000 nM of 5-azacytidine as top hits. The L1000CDS^2^ search using our gene results from 24 h after treatment with the high concentration of 5-azacytidine returned LINCS experiments corresponding to 24 h after treatment with 10,000 nM of 5-azacytidine as top hits, including the second best hit of all experiments. The search by L1000CDS^2^ using the list of genes in Table 1 from 24 h after treatment with the low concentration of 5-azacytidine returned experiments corresponding to 24 h after treatment with 3300 and 3370 nM of 5-azacytidine among the top hits. Interestingly, LINCS experiments involving nucleoside analogs kinetin riboside and thiazolopyrimidine were also returned among the top hits by the L1000CDS^2^ searches, whereas LINCS experiments involving inhibitors of purine biosynthesis, mercaptopurine, and thioguanosine were among the top LCD searches that used genes from Table 1 with concerted expression response to 5-azacytidine. These results support the suggestion that treatment with 5-azacytidine leads to concerted changes in the expression of genes that affect nucleotide synthesis, DNA biosynthesis, and DNA repair.

When we examined response to doxorubicin, L1000CDS^2^ identified LINCS experiments at 6 h after treatment with 10,000 nM of doxorubicin among the top hits for our searches that utilized the lists of genes from Table 1 with concerted changes at 6 h after treatment with the high concentration of the agent and at 24 h after treatment with the low concentration. L1000CDS^2^ searches that used both the lists of concerted genes and their median expression values returned LINCS experiments for doxorubicin analogs, 10,000 nM of epirubicin and 800 nM of 4-demethoxydaunorubicin, among the top hits for the input gene signatures from Table 1 corresponding to 2 and 6 h after treatment with the high concentration and 6 h after treatment with the low concentration of doxorubicin.

Among the agents examined in this study, vorinostat resulted in the highest number of positive matches to available independent datasets, suggesting that this agent induces strong concerted changes in the expression of genes involved in DNA methylation and one-carbon metabolism that are specific to HDAC inhibitors. We found multiple top matches to LINCS experiments at 6 and 24 h after treatment with 10,000 and 11,100 nM of vorinostat and/or after treatment with other HDAC inhibitors, trichostatin A and ISOX, when we used L1000CDS^2^ searches with lists of genes from 2 and 6 h after treatment with the high concentration and 2, 6, and 24 h after treatment with the low concentration of vorinostat. When L1000CDS^2^ searches utilized both gene lists in Table 1 and their median log_2_ FC values after treatment with vorinostat, a very large number of top matches from the LINCS database was found that included HDAC inhibitors vorinostat, trichostatin A, HDAC6 inhibitor ISOX, droxinostat (BRD-K11558771), belinostat (S1085), abexinostat (THM-I-94; PCI-24781), and dacinostat (BRD-K56957086). These strong matches were observed for both high and low concentrations and all the three time points after treatment with vorinostat in our dataset. They included the best hit for vorinostat experiments for each of the L1000CDS^2^ search that used our results at 6 h after treatment with the high and low concentrations and at 24 h after treatment with the low concentration of vorinostat. LINCS Canvas Browser (LCB) searches using genes in Table 1 involved in response to vorinostat also returned multiple high-ranking LINCS hits for vorinostat and trichostatin A. Among them, vorinostat experiments were the best match for each LCB search that used concerted gene lists from Table 1 at 6 h after treatment with the high and low concentrations of the agent and the second best hit for the gene list from 2 h after treatment with the high concentration.

Because of the small number of candidate genes with concerted expression response to paclitaxel (Table 1), searches for paclitaxel could only be conducted for the 24-h time point. An LCB search using genes with concerted response to the low concentration of paclitaxel returned experiments involving 24 h after treatment with 10,000 nM of docetaxel, an agent with a similar mechanism of action to paclitaxel, among the top hits. In addition, even though our gene lists were restricted to epigenetic components and OCM genes, both L1000CDS^2^ and LCB searches using genes with concerted response to high and low concentrations of paclitaxel returned several antimitotic and microtubule targeting agents including narciclasine, curcubitacin I, and vincristine, suggesting a possibility of some similar transcriptional response among epigenetic factors and OCM genes to these agents.

The searches of response to cisplatin could only be performed for the gene lists corresponding to 6 and 24 h after treatment with the high concentration of the agent because of the very limited number of genes with concerted expression changes for other conditions (Table 1). No LINCS experiments specific to cisplatin on other platinum agents were returned by any of these searches. This may suggest that, in contrast to other agents, the concerted changes among genes involved in epigenetic and OCM response to cisplatin that were identified in our study may not be specific to platinum compounds, and they may also occur in response to other classes of cancer agents. In contrast, our searches of independent datasets and perturbation experiments indicate that patterns of concerted changes in the expression of epigenetic and OCM genes in response to 5-azacytidine, doxorubicin, vorinostat, and paclitaxel may be specific to their classes of agents.

### Statistical significance of concerted expression changes

Additional file 1: Table S2 provides estimates of whether the number of genes with concerted regulation changes among the 56 candidate genes examined in this study is significantly greater than what would be expected for a random set of 56 genes. Concerted changes in the expression satisfied Bonferroni-adjusted threshold of statistical significance at 24 h of treatment with both high (*p* = 0.0001) and low (*p* < 0.0001) concentrations of paclitaxel. For several other agents and conditions, *p* values were below 0.05, but they did not achieve Bonferroni-adjusted threshold for multiple testing. These conditions include 6 h after treatment with the low concentration of 5-azacytidine, 6 and 24 h after treatment with the low concentration of doxorubicin, and 24 h after treatment with the high and low concentrations of vorinostat. Statistical significance of concerted changes after treatment with paclitaxel is likely explained by the small numbers of overall genome-wide concerted expression changes after treatment with this agent, as opposed to much more widespread concerted expression changes after treatment with other agents examined in this study. For example, 5-azacytidine and vorinostat lead to very strong and extensive genome-wide transcriptional changes that include concerted changes in the expression of many genes from different cellular pathways. The focus of our study was centered on components of one-carbon metabolism and of epigenetic DNA methylation and demethylation machinery, some of which, including DNMT1, represent direct biological targets of some of the agents that were examined. A number of these individual components of the epigenetic and the OCM pathways exhibited concerted changes in response to treatment. Some other genes in the genome, e.g., those involved in DNA repair, cell cycle regulation, and cell death, also had concerted expression changes in response to treatment, resulting in the total numbers of epigenetic and OCM components with concerted changes not being significant for agents other than paclitaxel. The absence of statistical significance of the total numbers of candidate genes with concerted changes is therefore due to large numbers of additional genes with concerted changes of expression, and it does not contradict the biological changes in the expression of individual components of DNA methylation, demethylation, and the OCM pathways in response to treatment.

### Correlation of cancer cell sensitivity to drug treatment with expression changes in genes involved in DNA methylation, demethylation, and the OCM pathway

When NCI-60 cell lines were treated with 5-azacytidine or vorinostat, the correlation between chemosensitivity, measured as log(GI50), and expression changes of any of the 56 candidate genes involved in DNA methylation, demethylation, and the OCM pathway was weak (none of the correlation tests reached significance, and in many cases Pearson’s |*r*| < 0.4). In addition, none of the 91 potential methylation target genes had statistically significant correlations with log(GI50) with after treatment with these agents. The absence of correlation between gene expression changes and chemosensitivity to 5-azacytidine is consistent with an earlier report that 5-azacytidine induced global genome-wide hypomethylation that prolonged survival of patients with myeloid malignancies but did not correlate with clinical prognosis or response to therapy [1]. In our analysis, epigenetic components of DNA methylation machinery and the OCM pathway members demonstrated considerable changes in their transcriptional response to treatment with 5-azacytidine (Table 1; Figs. 1 and 2, Additional file 2: Figures S1-S4); however, these changes were only weakly correlated with chemosensitivity (*r* < 0.4 for all 56 candidate genes listed in Table 1).

Table 2 provides the list of genes involved in DNA methylation, demethylation, and the OCM pathway that showed significant correlation of their expression changes with chemosensitivity to doxorubicin, paclitaxel, or cisplatin. All significant correlations were observed at 24 hours after treatment. After treatment with doxorubicin, expression changes of *FOLR2* and *SMUG1* were significantly negatively correlated with chemosensitivity. *FOLR2*, which encodes folate receptor 2, was upregulated at 24 h after treatment with the high concentration of doxorubicin, which was inversely correlated with chemosensitivity (Pearson’s *r* = −0.509, FDR adjusted *p* = 0.0366; Tables 1 and 2). This association is of interest because FOLR2 is currently used as a target for folate-mediated delivery of doxorubicin to cancer cells, and tumor cells with increased expression of *FOLR2* demonstrate enhanced cytotoxicity when treated with doxorubicin delivered in folate-dependent manner [100, 112]. For the dataset used in this study, the NCI-60 cell lines were grown on RPMI 1640 medium (Life Technologies, Inc.) that contained 1 μg/ml of folic acid and was supplemented with 5 % fetal bovine serum, and therefore, the presence of folate in the growth media may have contributed to FOLR2-dependent chemosensitivity to doxorubicin delivery and treatment.

*SMUG1* was upregulated at 24 h after treatment with doxorubicin, and this change was negatively correlated with chemosensitivity to the low concentration (*r* = −0.488, FDR adjusted *p* = 0.0284; Tables 1 and 2). SMUG1 is a member of the uracil-DNA glycosylase superfamily, which is involved in DNA repair and DNA demethylation [1, 6, 74]. The observed negative correlation between upregulation of *SMUG1* and chemosensitivity to doxorubicin is in agreement with the potential role of this gene in the removal, via the BER pathway, of DNA damage induced by doxorubicin (Additional file 1: Table S1) [94].

A significant correlation with chemosensitivity to paclitaxel was observed only for *GART*, which is involved in purine biosynthesis. It was downregulated at 24 h after treatment, and changes in its expression were significantly correlated with log(GI50) of the low concentration of paclitaxel (*r =* 0.462, FDR adjusted *p* = 0.0422; Tables 1 and 2).

Among the five cancer agents, the highest number of significant correlations of gene expression changes with chemosensitivity was observed at 24 h after treatment with cisplatin, with stronger correlations observed for the high concentration of that agent. As described above, chemosensitivity to cisplatin was also correlated with expression changes of multiple potential DNA methylation target genes. Among the 56 candidate genes from Table 1, significant correlations were observed for *GADD45A*, *MBD1*, *MTR*, *MTHFD1*, and *CTH* (Table 2). Components of DNA demethylation and DNA repair machinery *GADD45A* and *MBD1* were upregulated at 24 h after treatment, and their expression changes were inversely correlated with chemosensitivity (for *GADD45A*, *r* = −0.618, FDR adjusted *p* = 0.0011 for the high concentration and *r =* −0.477, *p* = 0.0422 for the low concentration of cisplatin, and for *MBD1*, *r* = −0.570, *p =* 0.0046 for the high concentration; Tables 1 and 2). The observed negative correlation for *MBD1* and *GADD45A* is in agreement with their previously reported roles in chemoresistance to cisplatin. Both genes participate in DNA repair and DNA damage checkpoint response, and inhibition of each gene has been reported to sensitize pancreatic and colon cancer cells to cisplatin and to radiation treatment [55, 113].

Expression changes of three OCM genes were also correlated with chemosensitivity to cisplatin (Table 2) at 24 h after treatment. Among them was *MTHFD1*, expression changes for which did not satisfy the criteria for a concerted pattern, but which demonstrated a significant positive correlation with chemosensitivity to the high concentration of cisplatin (*r =* 0.552, *p* = 0.0061). *MTR* was downregulated, which was significantly correlated with chemosensitivity to the high concentration (*r* = 0.564, *p* = 0.0046)*. CTH* was upregulated after treatment with the high concentration of the cisplatin (Table 1; Additional file 2: Figure S3D), and the changes in its expression were significantly inversely correlated with chemosensitivity (*r* = −0.531, *p =* 0.0122 after treatment with the high concentration and −0.504, *p =* 0.234 after treatment with the low concentration; Table 2).

## Conclusions

Our results suggest that despite a high level of genetic and cancer type heterogeneity among different tumor cell lines in the NCI-60 panel, multiple genes encoding epigenetic factors and members of the OCM pathway exhibited expression changes in the same direction across most of the cell lines in response to several cancer drugs analyzed in this study. Concerted changes in the expression of epigenetic and OCM pathways across different cell lines were observed even for antitumor agents such as paclitaxel and cisplatin that are not specifically targeting epigenetic machinery or folate-dependent cellular processes. Better understanding of epigenetic response and of changes in the OCM pathway in response to treatment may have implications for future design of drug combination therapies. Additional studies are needed to better understand how these changes correlate with DNA methylation status and functional activity of individual downstream target genes, which may affect chemosensitivity of cancer cells to treatment.

Concerted changes in gene expression were observed most frequently at 24 h after treatment with the high concentrations of antitumor agents, but for some individual genes, the peaks of their concerted changes were observed at 2 or 6 h. These observations suggest that a delay in administration of individual antitumor agents and the order of their administration may be beneficial when designing drug combination therapies. This may allow for additional treatment benefits based on the time needed for target genes to become upregulated or downregulated after administering the first agent, as compared to simultaneously administering the agents included in a combination. This observation, which is based on analysis of gene expression changes, is in agreement with earlier studies that suggested the benefits of time-staggered approach and the need to optimize the drug doses and times of their administration in drug combination therapies when eliciting apoptotic response or involvement of epigenetic machinery [114, 115]. Because the available dataset for this study was restricted to 2, 6, and 24 h following the drug treatment, further analyses may be needed to examine the optimal time points (e.g., those between 6 and 24 h or extending beyond the 24-h period) that would maximize the benefits of treatment using specific doses of individual antitumor agents. While the observed expression changes occurred rapidly, it may be possible that longer intervals exceeding 24 h may provide the full benefit of transition from molecular epigenetic changes to transcription, translation, and post-transcriptional and post-translation modification of target genes involved in cellular response.

The commonality of expression response patterns of individual molecular components across different histologic cancer types is of interest and may have clinical utility. It suggests that each cancer drug examined in this study induces some uniform biological response across a variety of cell lines, which involves specific epigenetic factors and reactions in the OCM pathway. The underlying molecular mechanisms of uniform response could be exploited in drug combination therapies that may target a variety of different cancer types. Two agents with broad epigenetic effects, 5-azacytidine and vorinostat, induced strong concerted changes in the expression of members of methylation machinery and members of the OCM pathway. Even though concerted transcriptional changes induced by these two drugs were only weakly correlated with chemosensitivity, this strong response is beginning to be successfully exploited in combination therapy treatments that aim to increase cytotoxicity or overcome chemoresistance to individual agents by downregulating or upregulating methylation target genes or genetic components of the OCM pathway [30, 65, 67, 115].

Treatment with doxorubicin led to concerted changes in the expression of DNA methylation machinery and of members of the OCM pathway, and chemosensitivity to doxorubicin was inversely correlated with upregulation of the folate receptor *FOLR2* gene*.* This finding further validates the ongoing efforts to increase the efficacy of doxorubicin treatment using the folate delivery system that targets this receptor [100, 112].

When the cell lines were treated with cisplatin, expression changes of several genes involved in DNA demethylation and repair and in the OCM pathway were correlated with chemosensitivity, suggesting their importance in response to treatment and in drug resistance. We also observed the correlation of transcriptional changes of a number of additional genes with chemosensitivity to cisplatin*.* The complex mechanisms of regulation of cellular response to cisplatin and the possible roles of DNA methylation, repair, and one-carbon metabolism reactions in this response require further investigation and may provide additional cues for increasing the effectiveness of treatment and for overcoming tumor resistance to this agent.

A number of combination therapy studies and clinical trials targeting epigenetic factors are currently underway [1, 28, 65, 66, 115, 116]. The benefits of targeting OCM pathway members in a drug combination therapy involving an antifolate drug, in some cases combined with an epigenetic cancer drug, are also becoming apparent [67]. A deeper understanding of molecular changes in epigenetic response and regulation of folate metabolism is important for appropriate timing and more focused targeting of molecular components of cancer cells. Changes in the expression of some of the important genes analyzed in this study suggest potential future directions of targeted epigenetic therapies with a focus on specific genes. For example, while the DNA methyltransferase *DNMT1* gene was downregulated after treatment with 5-azacytidine, doxorubicin, vorinostat, and paclitaxel, the direction of expression changes of *DNMT3A* and *DNMT3B* was specific to individual antitumor agents. *DNMT3A* was downregulated by vorinostat, but it showed a trend for upregulation in many cell lines after treatment with doxorubicin. *DNMT3B* was downregulated by doxorubicin, but it was upregulated after treatment with vorinostat. Further studies may examine whether combination treatment therapies involving doxorubicin and vorinostat may benefit from additional targeting of *DNMT3A* or *DNMT3B*, by using either broad epigenetic agents or antibodies specifically targeting these DNA methyltransferases. Similarly, upregulation of *TYMS* and *DHFR* after treatment with doxorubicin and cisplatin suggests a possibility that targeting products of these genes in a drug combination involving either of these drugs may provide additional cytotoxic benefits. Additional information in Table 1 that lists changes among multiple epigenetic factors and members of the OCM pathway in response to treatment may provide further suggestions for expanding the repertoire of molecular targets in combination treatment regimens.

## Methods

### Gene expression analysis

We investigated time-dependent effects of several cancer drugs on the expression of genes involved in DNA methylation, demethylation, and folate-dependent one-carbon metabolism pathways. For this purpose, we used longitudinal gene expression information derived from time course Affymetrix HG-U133A microarray expression experiments that involved the NCI-60 cancer cell line panel. These data were obtained from the TP Workbench of the National Cancer Institute. The TP Workbench tool provides visual and analytic representation of gene expression changes in the NCI-60 cancer cell lines treated with 15 anticancer drugs at 2, 6, and 24 h after treatment. Five of these drugs (5-azacytidine, doxorubicin, vorinostat, paclitaxel, and cisplatin) for which the data were available were selected for analysis in this study based on their reported effects on DNA methylation and/or the role of epigenetic factors in resistance to those agents. At each time point (2, 6, and 24 h) after treatment and for each of the 15 antitumor agents, expression levels were measured in all of the 60 cell lines treated with high and low concentrations of the drug and compared to time-matched control expression levels where cells were treated with vehicle only. The TP Workbench contains the dataset with expression measurements for 22,227 probe sets that were processed using background subtraction of CEL files and array normalization procedure using the robust multi-array average (RMA) algorithm [117]. The output expression values for each probe set corresponding to different probes within a single gene were combined from a single microarray by averaging log_2_ values of probe measurements, resulting in gene level-averaged output for 12,704 genes that were included in the Affymetrix U133A microarrays. Averaging of probe measurements for each gene was done separately and independently for each microarray dataset (for each given time point, and separately for untreated cell lines, cell lines treated with low drug concentration, and cell lines treated with a high drug concentration). The TP Workbench tool allows the users to view the post-treatment expression change for each gene in each individual cell line, presented as log_2_ of fold change of expression values of that gene (we refer to this value as log_2_ FC). The value of log_2_ FC is presented as a difference between the log_2_ of the averaged gene expression value in a cell line treated with a particular agent and the log_2_ of the averaged expression value of that gene in the same cell line that was treated only with vehicle and collected at time-matched intervals (2, 6, or 24 h after treatment). The detailed description of the data and tools in the Transcriptional Pharmacology Workbench and of experimental methodology that had been used to derive these data is provided by Monks et al. (Monks A, Zhao Y, Hose C, Fang J, Polley E, Harris E, Wu X,  Connelly J,  Rapisarda A, Teicher B,  Simon R, Doroshow JH.:Transcriptional Pharmacology Workbench: a powerful web tool to explore NCI60 time course data on genome-wide response to treatment with drugs, In preparation).

To monitor the concerted pattern of changes in each experiment, we introduced a threshold on the number of cell lines with discordant pattern of direction of change in gene expression. We denoted changes in expression as either upregulated or downregulated for those microarray experiments (specific to an antitumor agent, its concentration, and post-treatment time) in which the expression of nearly all NCI-60 cell lines changed in the same direction, and no more than 15 cell lines (*N* ≤ 15, i.e., no more than 25 % of the full dataset of 60 cell lines) had a change in the opposite direction. This filtering allowed us to identify the genes with the strongest trends for concerted expression changes among the cell lines. The probability that no more than 15 genes would be expressed in the opposite direction at random was computed based on the probability mass function of the binomial distribution using the *pbinom* function of the R environment, as the sum of *p*(*N* ≤ 15) + (1 − *p*(*N* ≤ 45)). We used genome-wide expression data to estimate the probability of any individual gene to be downregulated as opposed to being upregulated, separately for each drug, time point, and concentration. It was determined to be close to 0.5 for all 30 experiments (the range between 0.476 and 0.525). Based on these estimates and the numbers of cell lines with measured expression changes for each experiment, the probability of no more than 15 cell lines to be expressed in the opposite direction in a given experiment at random was found to be low, ranging between 6.96 × 10^−5^ and 1.54 × 10^−3^ for individual agents, time points, and drug concentrations.

### Drug concentrations

Expression levels of candidate genes involved in DNA methylation, demethylation, and one-carbon metabolism pathways were examined in cell lines treated with five antitumor agents, 5-azacytidine, doxorubicin, vorinostat, paclitaxel, and cisplatin. For each of the drugs, the high and low concentrations were chosen to approximate a clinically achievable concentration and an in vitro active concentration. They were, respectively, 5-azacytidine, 5000 and 1000 nM; doxorubicin, 1000 and 100 nM; vorinostat, 5000 and 1000 nM; paclitaxel, 100 and 10 nM; and cisplatin, 15000 and 3000 nM. For each drug concentration at each time point (2, 6, and 24 h after treatment), the TP Workbench output was provided as log_2_ FC of gene expression levels in each cell line treated with that concentration of the drug relative to those in the untreated cell line, measured at the same time point.

### Selection of candidate genes involved in DNA methylation, demethylation, and folate-mediated one-carbon metabolism pathway

Fifty-six candidate genes (Table 1; Additional file 1: Table S1) were selected for expression analysis based on their biological roles in methylation or demethylation of DNA or their involvement in the folate-mediated one-carbon metabolism pathway based on information from biomedical literature [5, 6, 8, 9, 11–15, 17, 18, 24, 60, 74, 77–79, 84, 118–122] and using information from the Kyoto Encyclopedia of Genes and Genomes (KEGG) [123], GeneCards [124], and the Online Mendelian Inheritance in Men (OMIM) [125] databases. Additional information about interactions of candidate genes with cancer drugs was obtained from the Drug Gene Interaction database (DGIdb) [126] and from the Pharmacogenomics Knowledgebase (PharmGKB) [127].

### Validation of concerted gene expression changes in other datasets

To confirm concerted gene expression changes in other datasets, we examined the gene signatures involved in transcriptional response to the drug treatment by searching the Molecular Signatures Database (MSigDB) v. 5.1 [72] and the biomedical literature for relevant treatment conditions. We also examined whether patterns of gene up- and downregulation observed in our study could be confirmed in independent expression datasets that included the cmap build 2 [110] and the LINCS.

The Connectivity Map provides data for gene expression changes in cell lines at 6 h after treatment, measured using the Affymetrix U133A expression microarrays. Available concentrations in cmap exceed the high concentrations in the TP Workbench, with the exception of one experiment (instance) of the MCF7 cell line-treated paclitaxel at 100 nM. All other available concentrations in cmap were 5-azacytidine, 16,400 nM; doxorubicin, 6800 nM; vorinostat, 10,000 nM; and paclitaxel, 4600 nM, with no data available for cisplatin. We used the lists of Affymetrix U133A microarray probesets for genes satisfying the criteria for concerted up- and downregulation at 6 h after treatment with the high concentrations in our study (Table 1), in order to analyze whether these groups of genes were among the top cmap rankings based on the degree of their expression changes, using online searches and permutation analysis provided by the Connectivity Map online interface [110].

The LINCS dataset utilizes expression measures for thousands of treatment conditions for approximately 1000 genes and imputed expression values for approximately 22,000 remaining genes [128]. We searched LINCS data using two search engines, L1000CDS^2^ and the LCB, which employ different approaches for finding similarities among gene sets representing each experiment [128–130]. LCB provides a search engine of at least 150,000 experimental conditions [128], whereas L1000CDS^2^ contains data on 389,031 precomputed gene signatures derived from LINCS data [130]. The lists of up- and downregulated genes with concerted expression changes (Table 1) for each agent, concentration, and time point were used as input for LCB and L1000CDS^2^. Additionally, we also used the L1000CDS^2^ search engine using gene signatures derived from Table 1 in our study, by providing the lists of genes with concerted regulation and their gene-specific median log_2_ FC values computed for each agent, treatment condition, and time point. These searches were conducted for those conditions in Table 1 that satisfied the search engine requirements for the number of input genes (≥3 of both upregulated and downregulated genes for a gene list or ≥5 genes for an input signature used by L1000CDS^2^; ≥2 genes with the same direction of concerted regulation for an input used by LCB). The results of the searches were considered to be among the top hits when they were returned in the default lists of hits (≤50 best hits for both search engines).

### Evaluation of statistical significance of the numbers of genes with concerted expression changes

In order to evaluate the statistical significance of the numbers of genes with concerted expression changes, we employed resampling analysis for each drug concentration and each time point. We used repeated sampling without replacement of 56 random genes from the 12,704 genes for which quality-processed expression data from the Affymetrix U133A expression microarray were available in the TP Workbench dataset. For each round of replications, we determined the number of genes satisfying the condition of co-regulation (the number of cell lines with expression in the opposite direction ≤15). For each agent, concentration, and time point, the empirical *p* value was estimated by computing the proportion of occurrences, out of 10,000 replications, when the number of co-regulated genes in a random sample was equal to or exceeded that for the 56 candidate genes in DNA methylation, demethylation, and OCM pathways for the same time point and concentration of the agent. The empirical *p* value threshold for statistical significance was adjusted for the number of tests (30 tests for 5 agents, 2 concentrations, and 3 time points) according to the Bonferroni procedure, resulting in adjusted threshold of 0.00167.

### Hierarchical clustering and heatmap analysis of expression response of potential gene targets of DNA methylation and demethylation pathways

Epigenetic action of some cancer drugs can reactivate expression of tumor suppressor genes and induce changes in the expression of genes involved in regulation of cell cycle, DNA repair, metabolic processes, and biosynthesis of important cell compounds [1, 2, 32, 37, 53, 89]. DNA demethylation is one of the mechanisms that cause transcriptional changes in response to cancer drug treatment [1, 53]. Additionally, epigenetic factors involved in DNA methylation and demethylation machinery contribute to the cytotoxic action of anticancer agents via the activation of the apoptotic pathways [1, 32, 34, 53]. To examine the simultaneous effects of cancer drug treatment on the expression of epigenetic factors and of their downstream targets, along with genes involved in OCM pathway, we analyzed the microarray data in the Transcriptional Pharmacology Workbench, by simultaneously monitoring changes in the expression of the 56 genes listed in Table 1 and 91 additional genes. These additional genes play important roles in cancer development and in cellular response to cancer drug treatment, and modulation of their expression levels in tumor cells prior to treatment and/or in response to treatment by one or more agents analyzed in this study have been suggested to involve components of epigenetic machinery, based on either direct methylation measures or indirect inference using gene expression information [1–3, 32, 34, 37, 44, 45, 49, 53, 86, 89, 91]. This list of potential DNA methylation and demethylation target genes included *BAK1*, *TP53* (*p53*), *TP53TG1* (*TP53AP1*), *RIPK2*, *BCL2*, *CCNB1*, *CASP5*, *CASP6*, *BRCA1*, *BRCA2*, *PTEN* (*p10*), *CDKN1A* (*p21*), *CDKN2A* (*p16*), *CDKN2B* (*p15*)*, CDKN1C* (*p57*)*, DLC1*, *TERT*, *SMG1*, *GTSE1*, *CHAF1A*, *RAD21*, *SMC3*, *SSRP1*, *HMGB2*, *CCNA2*, *RECQL*, *NEIL3*, *SMC5*, *TIPIN*, *ASF1A*, *NBN*, *CHEK1*, *DYRK2*, *ZAK* (*AZK*), *POLA1* (*POL1*), *POLB, SFPQ*, *PMS1*, *RAD50*, *KAT5* (*HTATIP*), *DCLRE1C*, *FAN1* (*MTMR15*), *CEBPG, FANCE*, *FANCF*, *UVRAG*, *MLH3*, *ATXN3*, *SMC6*, *UPF1, GTF2H1, NHEJ1*, *PPP1R15A* (*GADD34*), *FOXO3* (*FOXO3A*), *APTX*, *CCNO, TRIAP1*, *XPC*, *SFN* (*14-3-3σ*), *PTTG1*, *LIG1*, *BCL3*, *PNKP*, *MYO6*, *DDB2*, *SESN1*, *DDIT3*, *BTG2*, *MAP2K6*, *CD44*, *EDNRB* (*ETB*), *ESR1* (*ER-α*), *FHIT*, *GPC3*, *GSTP1*, *GATA1* (*GATA*), *MLH1*, *CD82* (*KAI1*), *FABP3* (*MDGI*), *ABCB1* (*MDR1*), *MUC2*, *MYOD1*, *NME1* (*NM23-H1*), *TP73* (*p73*), *PAX6, RARB* (*RARβ2*, or *NR1B2*), *RASSF1*, *TGFBR1*, *THBS1*, *TIMP3*, and *PLAU* (*uPA*)*.* Changes in the expression of these genes were analyzed using two-dimensional hierarchical clustering and heat maps. This clustering tool is provided by the TP Workbench, which utilizes the *hclust* function in the R package *heatmap.3*, with a “complete linkage” option for the agglomeration algorithm.

### Analysis of correlation of gene expression changes with chemosensitivity

For each drug concentration and each time point, we used information from the TP Workbench and additional analyses using R environment v. 3.1.2 to examine Pearson’s correlation between log_2_ FC of genes involved in DNA methylation, DNA demethylation, and the OCM pathway and their potential methylation targets and log(GI50) values of the NCI-60 cell lines. Here, GI50 is a measure of chemosensitivity of each NCI-60 cell line to an anticancer agent, measured as a concentration (μM) producing 50 % growth inhibition [31]. These GI50 values were generated from a standard NCI-60 screening experiment [51] initiated on the same day and with the same pot of cells that were used to generate these expression profiling data. Significance of correlation was evaluated according to the Benjamini-Hochberg [131] correction procedure for false discovery rate (FDR) using all *p* values from correlation analyses of five antitumor agents, three time points after treatment, and two concentrations of each drug. Genes and conditions with corrected *p* < 0.05 were noted as significantly associated with chemosensitivity or chemoresistance to treatment. All *p* values provided in this report have been adjusted according to the FDR procedure.

## Abbreviations

5-hmC, 5-hydroxymethylcytosine; AICDA (AID), activation-induced cytidine deaminase; ALDH1L1, 10-formyl tetrahydrofolate dehydrogenase (aldehyde dehydrogenase 1 family, member L1), cytosolic; ALDH2, aldehyde dehydrogenase 2, mitochondrial; AMT, aminomethyltransferase; APOBEC1, apolipoprotein B mRNA editing activity DNA deaminase 1; APOBEC2, apolipoprotein B mRNA editing activity DNA deaminase 2; APOBEC3A, apolipoprotein B mRNA editing activity DNA deaminase 3A; APOBEC3C, apolipoprotein B mRNA editing activity DNA deaminase 3C; ATIC, 5-aminoimidazole-4-carboxamide ribonucleotide formyltransferase; BER, base excision repair; BHMT, betaine-homocysteine methyltransferase; CBS, cystathionine β-synthase; CHFR, checkpoint with forkhead and ring finger domains; cmap, Connectivity Map; CNS, central nervous system; CTH, cystathionase; DGIdb, Drug Gene Interaction database; DHFR, dihydrofolate reductase; DLC1, deleted in liver cancer; DNMT, DNA 5′ cytosine-methyltransferase; DNMT1, DNA methyltransferase 1; DNMT3A, DNA methyltransferase 3A; DNMT3B, DNA methyltransferase 3B; DNMT3L, DNA methyltransferase 3-like protein; FC, fold change; FDH, 10-formyltetrahydrofolate dehydrogenase; FDR, false discovery rate; FOLH1 (GCPII, PSMA), folate hydrolase (glutamate carboxypeptidase II); FOLR1 (FRα), folate receptor 1; FOLR2 (FRβ), folate receptor 2; FOLR3 (FRγ), folate receptor 3; FTCD, glutamate formiminotransferase; GADD45A, growth arrest and DNA damage 45 protein A; GART, phosphoribosylglycinamide formyltransferase; Hcy, homocysteine; HDAC, histone deacetylase; IDH1, isocitrate dehydrogenase 1; IDH2, isocitrate dehydrogenase 2; KEGG, Kyoto Encyclopedia of Genes and Genomes; L1000CDS^2^, LINCS L1000 Characteristic Direction Signature Search Engine; LCB, LINCS Canvas Browser; LINCS, Library of Integrated Network-Based Cellular Signatures; MAT1A, L-methionine S-adenosyltransferase I, alpha; MAT2A, L-methionine S-adenosyltransferase II, alpha; MAT2B, L-methionine S-adenosyltransferase II, beta; MBD1, methyl-CpG-binding domain protein 1; MBD2, methyl-CpG-binding domain protein 2; MBD3, methyl-CpG-binding domain protein 3; MBD4 (MED1), methyl-CpG-binding domain protein 4; MeCP2, methyl-CpG-binding protein 2; MGMT, O(6)-methylguanine-DNA methyltransferase; MTHFD1, methylenetetrahydrafolate dehydrogenase 1; MTHFD2, methylenetetrahydrofolate dehydrogenase 2; MTHFD2L, methylenetetrahydrofolate dehydrogenase 2-like; MTHFR, 5,10-methylenetetrahydrafolate reductase; MTHFS, methylenetetrahydrofolate synthase; MTR, methionine synthase; MTRR, 5-methyltetrahydrafolate-homocysteine methyltransferase reductase; NNMT, nicotinamide *N*-methyltransferase; OCM, folate-mediated one-carbon metabolism; OMIM, Online Mendelian Inheritance in Men; PCNA, proliferating cell nuclear antigen; PEMT, phosphatidylethanolamine-*N*-methyltransferase; PharmGKB, Pharmacogenomics Knowledgebase; PON1, Paraoxonase 1; RMA, robust multi-array average; ROS, reactive oxygen species; SAH, *S*-adenosylhomocysteine; SAHA, suberoylanilide hydroxamic acid; SAM (AdoMet), *S*-adenosylmethionine; SFN, stratifin; SHMT1, serine hydroxymethyl transferase 1; SHMT2, serine hydroxymethyl transferase 2; SLC19A1 (RFC1), reduced folate carrier; SMUG1, single-strand-selective monofunctional uracil-DNA glycosylase; TCN2, transcobalamin II; TDG, thymine-DNA glycosylase; TERT (hTERT), human telomerase reverse transcriptase; TET3, Tet methylcytosine dioxygenase 3 (ten-eleven translocation-3); THF, tetrahydrafolate; TP, Transcriptional Pharmacology; TYMS (TS), thymidylate synthase; USP7 (HAUSP), Herpes virus-associated ubiquitin specific protease.

## Additional files

Additional file 1:
**Tables S1** and **S2**, and References for **Table S1**. (PDF 262 kb) Additional file 2: Figures S1-S17. (PDF 5851 kb)
