# Supplementary material for: Concerted changes in transcriptional regulation of genes involved in DNA methylation, demethylation, and folate-mediated one-carbon metabolism pathways in the NCI-60 cancer cell line panel in response to cancer drug treatment
Source: Clin Epigenetics. 2016 Jun 24;8:73. doi: 10.1186/s13148-016-0240-3 (PMC4919895; doi:10.1186/s13148-016-0240-3)
Supplement: Additional file 1: — Tables S1 and S2, and References for Table S1. (PDF 262 kb) [file 13148_2016_240_MOESM1_ESM.pdf]

Table S1. An overview of genes involved in DNA methylation, DNA demethylation, and one-carbon metabolism pathway that were analyzed in this study

Gene	Full Name	Role	Detailed Role	Reference[a]
DNMT1	DNA methyltransferase 1	Maintenance DNA methylation	Maintenance DNA methyltransferase	[1]
DNMT3A	DNA methyltransferase 3A	*De novo* DNA methylation	*De novo* DNA methylation	[1]
DNMT3B	DNA methyltransferase 3B	*De novo* DNA methylation	*De novo* DNA methylation	[1]
DNMT3L	DNA methyltransferase 3-like protein	Stimulates enzymatic activity of DNMT3A	Stimulation of enzymatic activity of DNMT3A	[1]
TET3	Tet methylcytosine dioxygenase 3 (ten-eleven translocation-3)	DNA demethylation	Conversion of 5-mC to 5-hmC, 5-fC and 5-caC	[1, 2]
AICDA (AID)	Activation-induced cytidine deaminase	DNA demethylation	DNA demethylation via deamination of 5-mC or 5-hmC	[2-4]
APOBEC1	Apolipoprotein B mRNA editing activity DNA deaminase 1	DNA demethylation	DNA demethylation via deamination of 5-mC or 5-hmC	[2-4]
APOBEC2	Apolipoprotein B mRNA editing activity DNA deaminase 2	DNA demethylation	DNA demethylation	[2-4]
APOBEC3A	Apolipoprotein B mRNA editing activity DNA deaminase 3A	DNA demethylation	DNA demethylation, possibly via deamination of 5-mC or 5-hmC	[2-4]
APOBEC3C	Apolipoprotein B mRNA editing activity DNA deaminase 3C	DNA demethylation	DNA demethylation, possibly via deamination of 5-mC or 5-hmC	[2-4]
TDG	Thymine-DNA glycosylase	DNA demethylation	Excision of target bases including 5-fC and 5-caC to initiate BER pathway	[2-4]
GADD45A	Growth arrest and DNA damage 45 protein A	DNA demethylation, DNA repair	DNA repair-mediated DNA demethylating factor that can reactivate genes which had been silenced by methylation; also involved in apoptosis	[5-8]
IDH1	Isocitrate dehydrogenase 1	Produces metabolites that interfere with TET-mediated DNA demethylation	IDH1 mutations lead to accumulation of 2-HG, TET inhibition and DNA hypermethylation	[2, 9-13]
IDH2	Isocitrate dehydrogenase 2	Produces metabolites that interfere with TET-mediated DNA demethylation	IDH2 mutations lead to accumulation of 2-HG, TET inhibition and DNA hypermethylation	[2, 9-13]
MGMT	O(6)-methylguanine-DNA methyltransferase	DNA repair via demethylation of O^6-meG	Demethylates O^6-methylguanine lesions. MGMT also removes larger O^6-alkyl	[14]

			adducts, and is thereby involved in resistance to nitrosourea-based anticancer drugs	
MBD1	Methyl-CpG-binding domain protein 1	Binding to methylated DNA, transcriptional modulation	Transcriptional repression, DNA repair	[1, 15-17]
MBD2	Methyl-CpG-binding domain protein 2	Transcriptional modulation, possible DNA demethylation, binding to methylated DNA	Possible roles in transcriptional activation or repression, possible DNA demethylation	[1, 15, 18]
MBD3	Methyl-CpG-binding domain protein 3	Binding to 5-hmC	Transcriptional repression	[1, 15]
MBD4 (MED1)	Methyl-CpG-binding domain protein 4	DNA repair	DNA repair, possible roles in demethylation and maintenance DNA methylation	[4, 16, 17]
MeCP2	Methyl-CpG-binding protein 2	Binding to methylated DNA, transcriptional modulation, forms a complex with TET1	Transcriptional repression, participation in TET1 complexes that lead to DNA demethylation	[1, 15]
PCNA	Proliferating cell nuclear antigen	DNA repair and replication; interactions with DNMT1 and TET1	Participates in DNA repair and replication. It may affect both DNA methylation via interaction with DNMT1 and DNA demethylation by forming a complex with TET1	[11, 19]
USP7 (HAUSP)	Herpes virus–associated ubiquitin specific protease	Promotes DNA methylation via control of DNMT1	Regulates DNMT1 abundance, stability and activity	[19, 20]
SMUG1	Single-strand-selective monofunctional uracil-DNA glycosylase	DNA demethylation, DNA repair	A member of the uracil-DNA glycosylase superfamily which is involved in DNA repair and DNA demethylation via the base excision repair (BER) pathway by participating in degradation of 5-hydroxymethyluracil (5-hmU) to unmethylated cytosine	[1, 2, 4]
MTHFR	5, 10-methylenetetrahydrafolate reductase	OCM: regulation of folate metabolism	Catalyzes one of the central OCM reactions: the NADPH-dependent reduction of 5,10-methylenetetrahydrafolate (5,10-methyleneTHF) to 5-methylTHF	[21, 22]
MTHFD1	Methylenetetrahydrafolate dehydrogenase 1	OCM: Cytoplasmic roles as FTHF synthetase, CH^{+}-THF cyclohydrolase, and CH_2-THF dehydrogenase	Combines the functions of 10-formyl-THF synthetase, 5,10-methenyl-THF cyclohydrolase, and 5,10-methylene-THF	[21, 23, 24]

			dehydrogenase that catalyze the reversible interconversion in the cytoplasm of THF into 5,10-methyleneTHF, via 10-formylTHF and 5,10-methenylTHF. These reactions produce 5,10-methyleneTHF, a cofactor required for thymidylate biosynthesis.	
MTR	Methionine synthase	OCM: remethylation of Hcy to methionine	Remethylates Hcy to methionine	[23]
MTRR	5-methyltetrahydrafolate-homocysteine methyltransferase reductase	OCM: generates functional methionine synthase	Produces active methionine synthase, MTR	[25, 26]
CBS	Cystathionine β-synthase	OCM: catalyzes the condensation of Hcy and Ser to cystathionine	Participates in the reactions that lead to the conversion of Hcy to cysteine, removing Hcy from the methylation cycle	[25]
TCN2	Transcobalamin II	OCM: vitamin B12 transport	Transport protein for cobalamin	[27]
SHMT1	Serine hydroxymethyl transferase 1	OCM: catalyzes the reversible conversion of Ser and THF to glycine and CH_2-THF	Catalyzes the synthesis of 5-formylTHF from 5,10-methenylTHF in the cytoplasm and limits the availability of 5-methylTHF for Hcy remethylation and SAM biosynthesis; some reports also noted its possible activity in the nucleus	[21]
TYMS (TS)	Thymidylate synthase	OCM: catalyzes the conversion of dUMP to dTMP	Catalyzes the 5,10-methyleneTHF-dependent conversion of deoxyuridinemonophosphate (dUMP) into deoxythymidine monophosphate (dTMP), which serves as a precursor for DNA synthesis and is used DNA repair	[21, 23]
DHFR	Dihydrofolate reductase	OCM: conversion of dihydrofolate to THF	Catalyzed the reduction of dihydrofolate (DHF) to THF	[21, 23]
BHMT	Betaine-homocysteine methyltransferase	OCM: remethylation of Hcy to methionine	Participates in remethylation of Hcy to methionine via a reaction that is an alternative to the reactions regulated by MTR and MTRR	[23, 28]
CTH	Cystathionase (cystathionine γ-lyase)	OCM: conversion of cystathione to cysteine	Irreversible degradation of cystathionine, which is derived from Hcy, to cysteine, which contributes to removal of Hcy from	[25, 29, 30]

			the methylation cycle	
AHCY (SAHH)	S-adenosyl-L-homocysteine hydrolase	OCM: hydrolysis of SAH to adenosine and Hcy	Catalyzes reversible hydrolysis of *S*-adenosyl-*L*-homocysteine to Hcy and adenosine	[30, 31]
ALDH1L1	10-formyl tetrahydrofolate dehydrogenase (aldehyde dehydrogenase 1 family, member L1), cytosolic	OCM: irreversible oxidation of FTHF to THF and CO_2	Encodes 10-formyltetrahydrofolate dehydrogenase (FDH), a major regulator of folate metabolism in the cytoplasm via $NADP^+$ -dependent irreversible oxidation of 10-formylTHF to THF. This process controls the availability of folate-bound carbon groups for biosynthetic processes, cell growth, and remethylation of Hcy, and it affects the availability of methyl groups for cellular methylation reactions	[32, 33]
ATIC	5-aminoimidazole-4-carboxamide ribonucleotide formyltransferase	OCM: purine biosynthesis	Purine biosynthesis	[34]
GART	Phosphoribosylglycinamide formyltransferase	OCM: purine biosynthesis	Purine biosynthesis	[34]
MTHFS	Methylenetetrahydrofolate synthase	OCM: purine biosynthesis	Encodes 5,10-methenylTHF synthetase which catalyzes the irreversible conversion of 5-formylTHF to 5,10-methenylTHF	[21]
FTCD	Glutamate formiminotransferase	OCM: histidine catabolism	Provides one-carbon units resulting from histidine catabolism to the folate pool	[21]
MAT1A	L-methionine S-adenosyltransferase I, alpha	OCM: catalyzes SAM biosynthesis from methionine and ATP	Catalyzes biosynthesis of SAM, the major source of methyl groups for methylation reactions	[35]
MAT2A	L-methionine S-adenosyltransferase II, alpha	OCM: catalyzes SAM biosynthesis from methionine and ATP	Catalyzes biosynthesis of SAM	[35]
MAT2B	L-methionine S-adenosyltransferase II, beta	OCM: catalyzes SAM biosynthesis from methionine and ATP	Catalyzes biosynthesis of SAM	[35]
NNMT	Nicotinamide N-methyltransferase	OCM: N-methylation of nicotinamide and other pyridines using SAM as methyl donor	Generates 1-methylnicotinamide in a reaction that consumes methyl units from SAM and reduces the ratio of SAM to Hcy	[36, 37]
PON1	Paraoxonase 1	OCM: generates Hcy from homocysteine thiolactone	Generates Hcy from homocysteine thiolactone	[38]
SLC19A1	Reduced folate carrier	OCM: transport of folate and drugs	Major transporter of folate and of antifolate	[22, 25,

(RFC1)		across cell membrane	cancer drugs across cell membrane	39-42]
FOLR1 (FRα)	Folate receptor 1	OCM: folate endocytosis	Folate endocytosis	[40]
FOLR2 (FRβ)	Folate receptor 2	OCM: folate endocytosis	Folate endocytosis	[40]
FOLR3 (FRγ)	Folate receptor 3	OCM: folate endocytosis	Folate endocytosis	[40]
SHMT2	Serine hydroxymethyl transferase 2	OCM: mitochondrial folate metabolism for glycine synthesis	Encodes the mitochondrial isozyme of serine hydroxymethyltransferase	[43]
AMT	Aminomethyltransferase	OCM: glycine cleavage system in mitochondria	Involved in the glycine cleavage system in mitochondria that generates 5,10-methylene-THF	[21]
MTHFD2	Methylenetetrahydrofolate dehydrogenase 2	OCM: mitochondrial NAD^+-dependent CH_2-THF dehydrogenase and CH^+-THF cyclohydrolase	Bifunctional mitochondrial NAD^+-dependent 5,10-methylene-THF dehydrogenase /5,10-methenyl-THF cyclohydrolase	[43]
MTHFD2L	Methylenetetrahydrofolate dehydrogenase 2-like	OCM: Mitochondrial dual redox cofactor-specific CH_2-THF dehydrogenase and CH^+-THF cyclohydrolase	5,10-methyleneTHF dehydrogenase and 5,10-methenyl-THF cyclohydrolase in the mitochondrial OCM pathway	[18]
PEMT	Phosphatidylethanolamine-N-methyltransferase	OCM: biosynthesis of phosphatidyl choline via interaction with SAM	Catalyzes the *de novo* synthesis of phosphatidylcholine using SAM as methyl donor	[30]
FOLH1 (GCPII, PSMA)	Folate hydrolase (glutamate carboxypeptidase II)	Conversion of dietary folate to folate and its intestinal absorption	Intestinal absorption of dietary folate and its conversion to folate	[24]
ALDH2	Aldehyde dehydrogenase 2 (mitochondrial)	Metabolizes acetaldehyde, which may affect folate levels and inhibit DNA methylation	Affects folate levels *in vivo* by producing high levels of acetaldehyde in alcohol metabolism and reducing the cleavage of folate	[44]

2-HG, 2-hydroxyglutarate**; 5-hmU**, 5-hydroxymethyluracil; **5-caC**, 5-carboxylcytosine; **5-fC,** 5-formylcytosine; **5-hmC,** 5-hydroxymethylcytosine; **5-mC,** 5-methylcytosine; **BER,** base excision repair; **CH^+-THF**, 5,10-methenyl-tetrahydrofolate; **CH_2-THF**, 5,10-methylene-tetrahydrofolate; **FDH**, 10-formyltetrahydrofolate dehydrogenase; **FTHF**, 10-formyl-tetrahydrafolate; **Hcy**, homocysteine; **OCM**, folate-mediated one-carbon metabolism pathway; **SAH**, *S*-adenosylhomocysteine; **SAM,** *S*-adenosylmethionine; **Ser**, serine; **THF**, tetrahydrafolate

[a] The list of references for Table S1 is provided after Table S2.

Table S2. Numbers of genes with concerted expression and their empirical *p*-values for each treatment condition

**Drug**	**Concentration**	**Time (hours)**	**Number of genes with concerted expression**	***P*-value**
5-Azacytidine	High	2	3	0.6237
5-Azacytidine	High	6	15	0.1694
5-Azacytidine	High	24	36	0.3517
5-Azacytidine	Low	2	2	0.4805
5-Azacytidine	Low	6	8	0.0245**
5-Azacytidine	Low	24	24	0.1042
Doxorubicin	High	2	5	0.9307
Doxorubicin	High	6	20	0.1309
Doxorubicin	High	24	28	0.4328
Doxorubicin	Low	2	1	0.6757
Doxorubicin	Low	6	7	0.0205**
Doxorubicin	Low	24	19	0.0034**
Vorinostat	High	2	11	0.2011
Vorinostat	High	6	24	0.5076
Vorinostat	High	24	33	0.0076**
Vorinostat	Low	2	9	0.3379
Vorinostat	Low	6	23	0.3160
Vorinostat	Low	24	24	0.0463**
Paclitaxel	High	2	0	1.0000
Paclitaxel	High	6	0	1.0000
Paclitaxel	High	24	16	0.0001*
Paclitaxel	Low	2	0	1.0000
Paclitaxel	Low	6	1	0.4355
Paclitaxel	Low	24	16	0.0000*
Cisplatin	High	2	0	1.0000
Cisplatin	High	6	10	0.1481
Cisplatin	High	24	17	0.4196
Cisplatin	Low	2	1	0.2785
Cisplatin	Low	6	0	1.0000
Cisplatin	Low	24	5	0.1270

Empirical *p*-values were computed using 10,000 replications of random sampling of 56 genes from the 12,704 genes for which expression data were available in the TP Workbench. Genes were determined to have concerted expression when nearly all cell lines had a change in the same direction, with ≤ 15 cell lines showing a change in the opposite direction.

* $P < 0.00167$ (Bonferroni-adjusted *p*-value threshold for 5 agents, 2 concentrations, and 3 time points)

** $P < 0.05$

References for Table S1

1. Pleyer L, Greil R. Digging deep into "dirty" drugs - modulation of the methylation machinery. Drug Metab Rev. 2015;47:252-79.
2. Shen L, Song CX, He C, Zhang Y. Mechanism and function of oxidative reversal of DNA and RNA methylation. Annu Rev Biochem. 2014;83:585-614.
3. Sadakierska-Chudy A, Kostrzewa RM, Filip M. A comprehensive view of the epigenetic landscape part I: DNA methylation, passive and active DNA demethylation pathways and histone variants. Neurotox Res. 2015;27:84-97.
4. Franchini DM, Petersen-Mahrt SK. AID and APOBEC deaminases: balancing DNA damage in epigenetics and immunity. Epigenomics. 2014;6:427-43.
5. Niehrs C, Schafer A. Active DNA demethylation by Gadd45 and DNA repair. Trends Cell Biol. 2012;22:220-7.
6. Schneider-Stock R, Diab-Assef M, Rohrbeck A, Foltzer-Jourdainne C, Boltze C, Hartig R et al. 5-Aza-cytidine is a potent inhibitor of DNA methyltransferase 3a and induces apoptosis in HCT-116 colon cancer cells via Gadd45- and p53-dependent mechanisms. J Pharmacol Exp Ther. 2005;312:525-36.
7. Nguyen AN, Hollenbach PW, Richard N, Luna-Moran A, Brady H, Heise C et al. Azacitidine and decitabine have different mechanisms of action in non-small cell lung cancer cell lines. Lung Cancer: Targets and Therapy. 2010;1:119-40.
8. Barreto G, Schafer A, Marhold J, Stach D, Swaminathan SK, Handa V et al. Gadd45a promotes epigenetic gene activation by repair-mediated DNA demethylation. Nature. 2007;445:671-5.
9. Shen H, Laird PW. Interplay between the cancer genome and epigenome. Cell. 2013;153:38-55.
10. Baubec T, Ivanek R, Lienert F, Schubeler D. Methylation-dependent and -independent genomic targeting principles of the MBD protein family. Cell. 2013;153:480-92.
11. Cartron PF, Nadaradjane A, Lepape F, Lalier L, Gardie B, Vallette FM. Identification of TET1 Partners That Control Its DNA-Demethylating Function. Genes Cancer. 2013;4:235-41.
12. Prensner JR, Chinnaiyan AM. Metabolism unhinged: IDH mutations in cancer. Nat Med. 2011;17:291-3.
13. Suetake I, Shinozaki F, Miyagawa J, Takeshima H, Tajima S. DNMT3L stimulates the DNA methylation activity of Dnmt3a and Dnmt3b through a direct interaction. J Biol Chem. 2004;279:27816-23.
14. Fahrer J, Kaina B. O6-methylguanine-DNA methyltransferase in the defense against N-nitroso compounds and colorectal cancer. Carcinogenesis. 2013;34:2435-42.
15. Das PM, Singal R. DNA methylation and cancer. J Clin Oncol. 2004;22:4632-42.
16. Xu J, Zhu W, Xu W, Cui X, Chen L, Ji S et al. Silencing of MBD1 reverses pancreatic cancer therapy resistance through inhibition of DNA damage repair. Int J Oncol. 2013;42:2046-52.
17. Prokhortchouk E, Hendrich B. Methyl-CpG binding proteins and cancer: are MeCpGs more important than MBDs? Oncogene. 2002;21:5394-9.
18. Cheishvili D, Chik F, Li CC, Bhattacharya B, Suderman M, Arakelian A et al. Synergistic effects of combined DNA methyltransferase inhibition and MBD2 depletion on breast cancer cells; MBD2 depletion blocks 5-aza-2'-deoxycytidine-triggered invasiveness. Carcinogenesis. 2014;35:2436-46.
19. Bronner C. Control of DNMT1 abundance in epigenetic inheritance by acetylation, ubiquitylation, and the histone code. Sci Signal. 2011;4:pe3.
20. Varol N, Konac E, Bilen CY. Does Wnt/beta-catenin pathway contribute to the stability of DNMT1 expression in urological cancer cell lines? Exp Biol Med (Maywood). 2014.
21. Fox JT, Stover PJ. Folate-mediated one-carbon metabolism. Vitam Horm. 2008;79:1-44.
22. Ulrich CM, Robien K, McLeod HL. Cancer pharmacogenetics: polymorphisms, pathways and beyond. Nat Rev Cancer. 2003;3:912-20.
23. Blom HJ, Smulders Y. Overview of homocysteine and folate metabolism. With special references to cardiovascular disease and neural tube defects. J Inherit Metab Dis. 2011;34:75-81.

24. Hazra A, Wu K, Kraft P, Fuchs CS, Giovannucci EL, Hunter DJ. Twenty-four non-synonymous polymorphisms in the one-carbon metabolic pathway and risk of colorectal adenoma in the Nurses' Health Study. Carcinogenesis. 2007;28:1510-9.
25. Nazki FH, Sameer AS, Ganaie BA. Folate: metabolism, genes, polymorphisms and the associated diseases. Gene. 2014;533:11-20.
26. Mostowska A, Myka M, Lianeri M, Roszak A, Jagodzinski PP. Folate and choline metabolism gene variants and development of uterine cervical carcinoma. Clin Biochem. 2011;44:596-600.
27. Martinelli M, Scapoli L, Mattei G, Ugolini G, Montroni I, Zattoni D et al. A candidate gene study of one-carbon metabolism pathway genes and colorectal cancer risk. Br J Nutr. 2013;109:984-9.
28. Teng YW, Mehedint MG, Garrow TA, Zeisel SH. Deletion of betaine-homocysteine S-methyltransferase in mice perturbs choline and 1-carbon metabolism, resulting in fatty liver and hepatocellular carcinomas. J Biol Chem. 2011;286:36258-67.
29. Iacobazzi V, Castegna A, Infantino V, Andria G. Mitochondrial DNA methylation as a next-generation biomarker and diagnostic tool. Mol Genet Metab. 2013;110:25-34.
30. Tehlivets O, Malanovic N, Visram M, Pavkov-Keller T, Keller W. S-adenosyl-L-homocysteine hydrolase and methylation disorders: yeast as a model system. Biochim Biophys Acta. 2013;1832:204-15.
31. Li Q, Mao L, Wang R, Zhu L, Xue L. Overexpression of S-adenosylhomocysteine hydrolase (SAHH) in esophageal squamous cell carcinoma (ESCC) cell lines: effects on apoptosis, migration and adhesion of cells. Mol Biol Rep. 2014;41:2409-17.
32. Oleinik NV, Krupenko NI, Krupenko SA. Epigenetic Silencing of ALDH1L1, a Metabolic Regulator of Cellular Proliferation, in Cancers. Genes Cancer. 2011;2:130-9.
33. Stover PJ. Polymorphisms in 1-carbon metabolism, epigenetics and folate-related pathologies. J Nutrigenet Nutrigenomics. 2011;4:293-305.
34. Lane AN, Fan TW. Regulation of mammalian nucleotide metabolism and biosynthesis. Nucleic Acids Res. 2015;43:2466-85.
35. Murray B, Antonyuk SV, Marina A, Van Liempd SM, Lu SC, Mato JM et al. Structure and function study of the complex that synthesizes S-adenosylmethionine. IUCrJ. 2014;1:240-9.
36. Ulanovskaya OA, Zuhl AM, Cravatt BF. NNMT promotes epigenetic remodeling in cancer by creating a metabolic methylation sink. Nat Chem Biol. 2013;9:300-6.
37. Shlomi T, Rabinowitz JD. Metabolism: Cancer mistunes methylation. Nat Chem Biol. 2013;9:293-4.
38. Jakubowski H, Zhang L, Bardeguez A, Aviv A. Homocysteine thiolactone and protein homocysteinylation in human endothelial cells: implications for atherosclerosis. Circ Res. 2000;87:45-51.
39. Matherly LH, Wilson MR, Hou Z. The major facilitative folate transporters solute carrier 19A1 and solute carrier 46A1: biology and role in antifolate chemotherapy of cancer. Drug Metab Dispos. 2014;42:632-49.
40. Zhao R, Goldman ID. Folate and thiamine transporters mediated by facilitative carriers (SLC19A1-3 and SLC46A1) and folate receptors. Mol Aspects Med. 2013;34:373-85.
41. Hou Z, Matherly LH. Biology of the major facilitative folate transporters SLC19A1 and SLC46A1. Curr Top Membr. 2014;73:175-204.
42. Hagner N, Joerger M. Cancer chemotherapy: targeting folic acid synthesis. Cancer Manag Res. 2010;2:293-301.
43. Shin M, Bryant JD, Momb J, Appling DR. Mitochondrial MTHFD2L is a dual redox cofactor-specific methylenetetrahydrofolate dehydrogenase/methenyltetrahydrofolate cyclohydrolase expressed in both adult and embryonic tissues. J Biol Chem. 2014;289:15507-17.
44. Otani T, Iwasaki M, Hanaoka T, Kobayashi M, Ishihara J, Natsukawa S et al. Folate, vitamin B6, vitamin B12, and vitamin B2 intake, genetic polymorphisms of related enzymes, and risk of colorectal cancer in a hospital-based case-control study in Japan. Nutr Cancer. 2005;53:42-50.
