# Supplementary material for: Concerted changes in transcriptional regulation of genes involved in DNA methylation, demethylation, and folate-mediated one-carbon metabolism pathways in the NCI-60 cancer cell line panel in response to cancer drug treatment
Source: Clin Epigenetics. 2016 Jun 24;8:73. doi: 10.1186/s13148-016-0240-3 (PMC4919895; doi:10.1186/s13148-016-0240-3)
Supplement: Additional file 2: Figures S1-S17. — (PDF 5851 kb) [file 13148_2016_240_MOESM2_ESM.pdf]

Additional File 2: Figures S1-S17

Figure S1. Heat map and two-dimensional clustering of gene expression changes (log_2 difference in expression values between treated and untreated cell lines) of members of DNA methylation and demethylation pathways, the OCM pathway, and potential methylation target genes in the NCI-60 cell lines at 24 hours after treatment with the high concentration (5,000 nM) of 5-azacytidine. NCI-60 cell lines are represented by columns, whereas individual genes are represented by rows.

Figure S2. Changes in expression of selected genes (*PCNA*, *GADD45A*, *MBD4*, *TDG*, *MTHFD1*, and *MTHFD2*) at 2 (left panel), 6 (middle panel), and 24 hours (right panel) after treatment with the high concentration (5,000 nM) of 5-azacytidine. Horizontal right bars indicate elevated gene expression, whereas left bars decreased expression relative to cell lines untreated by drug. Colors represent different types of cancer tissues (breast, central nervous system (CNS), colon, leukemia, lung, melanoma, ovarian, prostate, and renal cancers). The scale on the bottom represents log_2 difference between expression values of treated and untreated cell lines. The scale for each microarray experiment is specific to that experiment.

Figure S3. Transcriptional changes of *CTH* at 2 (left panel), 6 (middle panel), and 24 hours (right panel) after treatment with high concentrations of **(A)** 5-azacytidine (5,000 nM); **(B)** doxorubicin (1,000 nM); **(C)** vorinostat (5,000 nM); and **(D)** cisplatin (15,000 nM). Additional information about data representation is provided in the legend to Fig. S2.

Figure S4. Drug-specific response patterns of expression changes of *DHFR* at 2 (left panel), 6 (middle panel), and 24 hours (right panel) after treatment with high concentrations of **(A)** 5-azacytidine (5,000 nM); **(B)** doxorubicin (1,000 nM); **(C)** vorinostat (5,000 nM); **(D)** paclitaxel (100 nM); and **(E)** cisplatin (15,000 nM). Additional information is provided in the legend to Fig. S2.

Figure S5. Heat map and two-dimensional clustering of gene expression changes of members of DNA methylation and demethylation pathways, the OCM pathway, and potential methylation target genes in the NCI-60 cell lines at 24 hours after treatment with the high concentration (1,000 nM) of doxorubicin. Individual genes are represented by rows. Cell lines are represented by columns, with the right most column representing the T-47D cell line with a distinct pattern of expression changes.

Figure S6. Changes in expression of *DNMT3B* at 2 (left panel), 6 (middle panel), and 24 hours (right panel) after treatment with high concentrations of **(A)** doxorubicin (1,000 nM); and **(B)** vorinostat (5,000 nM). Additional information is provided in the legend to Fig. S2.

Figure S7. Changes in expression of *PCNA*, *GADD45A*, *MBD4*, and *MECP2* involved in DNA methylation, demethylation, and DNA repair at 2 (left panel), 6 (middle panel), and 24 hours (right panel) after treatment with the high concentration (1,000 nM) of doxorubicin. Additional information is provided in the legend to Supplementary Fig. 2. The opposite direction of expression changes in the T-47D cell line at 6 and/or 24 hours after treatment as compared to the direction of changes in the majority of other NCI-60 cell lines can be seen in the middle and right panels for each gene.

Figure S8. Changes in expression of selected OCM pathway genes (*ATIC, MAT2A, MTHFS,* and *MTR*) at 2 (left panel), 6 (middle panel), and 24 hours (right panel) after treatment with the high (1,000 nM) or low (500 nM) concentrations of doxorubicin. Additional information is provided in the legend to Supplementary Fig. 2. Upregulation of expression of the T-47D cell line at 24 hours after treatment as opposed to downregulation of the majority of other NCI-60 cell lines can be seen in the right panels for each gene.

Figure S9. Heat map and two-dimensional clustering of gene expression changes of members of DNA methylation and demethylation pathways, the OCM pathway, and potential methylation target genes at 24

hours after treatment with the high concentration (5,000 nM) of vorinostat. Additional information about data representation is provided in the legend to Fig. S1.

Figure S10. Changes in *DNMT3A* expression at 2 (left panel), 6 (middle panel), and 24 hours (right panel) after treatment with vorinostat: **(A)** high concentration (5,000 nM); **(B)** low concentration (1,000 nM). Additional information about data representation is provided in the legend to Fig. S2.

Figure S11. Changes in expression of *APOBEC3C, TDG, MBD1, MBD3, MBD4,* and *MECP2* involved in DNA methylation, demethylation, and DNA repair at 2 (left panel), 6 (middle panel), and 24 hours (right panel) after treatment with the high concentration (5,000 nM) of vorinostat. Additional information about data representation is provided in the legend to Fig. S2.

Figure S12. Changes in gene expression of selected members of the OCM pathway (*SLC19A1, FOLR1, MAT2B, MTR, ATIC, MTHFS,* and *MTHFD1*) at 2 (left panel), 6 (middle panel), and 24 hours (right panel) after treatment with the high concentration (5,000 nM) of vorinostat. Additional information is provided in the legend to Fig. S2.

Figure S13. Heat map and two-dimensional clustering of gene expression changes of members of DNA methylation and demethylation pathways, the OCM pathway, and potential methylation target genes in the NCI-60 cell lines at 24 hours after treatment with the high concentration (100 nM) of paclitaxel. Additional information is provided in the legend to Fig. S1.

Figure S14. Changes in expression of *PCNA*, *TDG*, *IDH1*, and *GART* at 2 (left panel), 6 (middle panel), and 24 hours (right panel) after treatment with the high concentration (1,000 nM) of paclitaxel. Additional information is provided in the legend to Fig. S2.

Figure S15. Heat map and two-dimensional clustering of gene expression changes of members of DNA methylation and demethylation pathways, the OCM pathway, and potential methylation target genes in the NCI-60 cell lines at 24 hours after treatment with the high concentration (15,000 nM) of cisplatin. Additional information is provided in the legend to Fig. S1.

Figure S16. Changes in expression of selected genes (*PCNA*, *GADD45A*, *MGMT*, *MBD1, MBD4*, and *USP7*) involved in DNA methylation, demethylation, and DNA repair pathways at 2 (left panel), 6 (middle panel), and 24 hours (right panel) after treatment with the high concentration (15,000 nM) of cisplatin. Additional information is provided in the legend to Fig. S2.

Figure S17. Changes in expression of selected members of the OCM pathway (*MAT2A, PEMT, MTHFD2*, and *MTHFD2L*) at 2 (left panel), 6 (middle panel), and 24 hours (right panel) after treatment with the high concentration (15,000 nM) of cisplatin. Additional information is provided in the legend to Fig. S2.

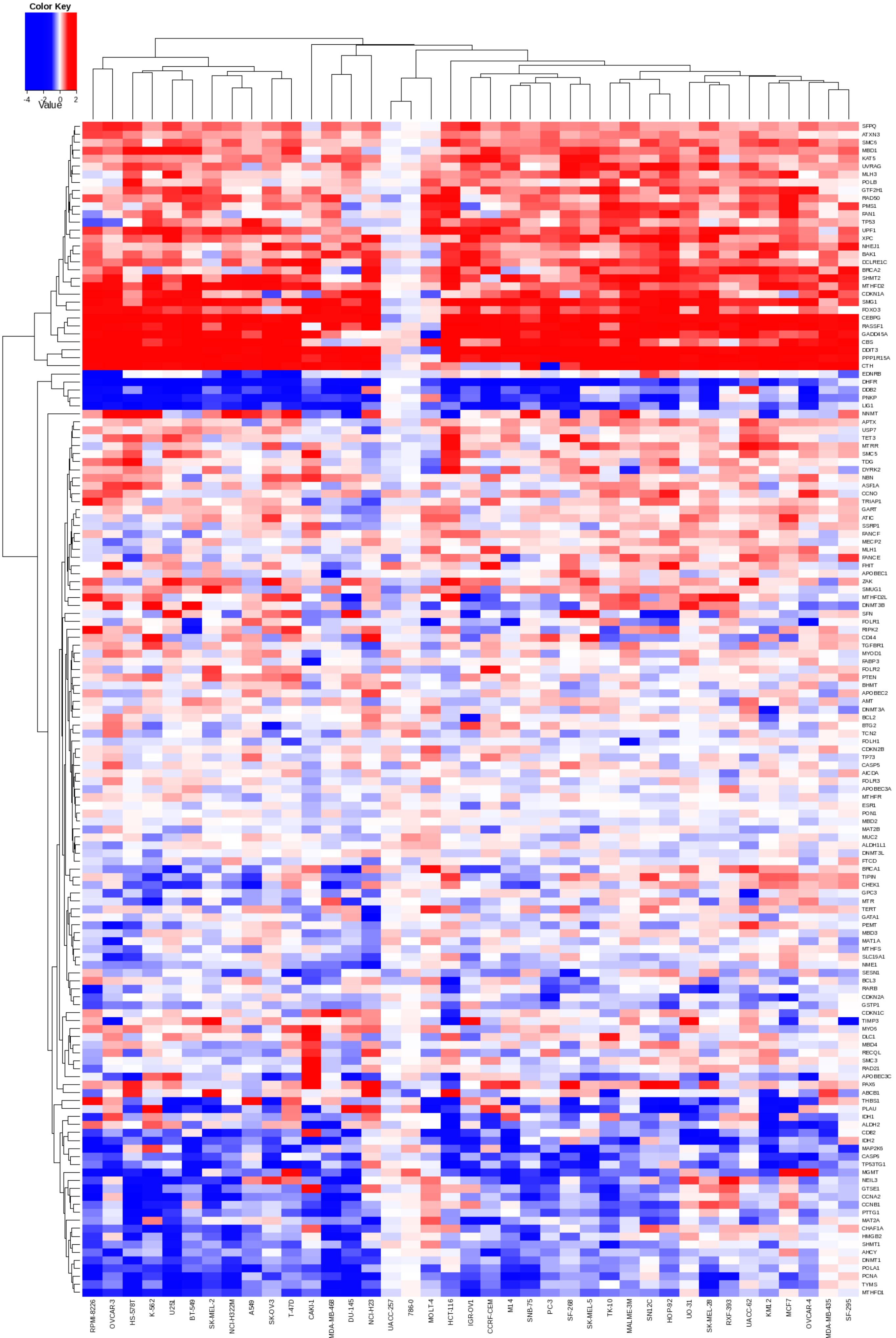
Color Key
-4 -2 0 2
Value
SFPQ
ATXN3
SMC6
MBD1
KAT5
UVRAG
MLH3
POLB
GTF2H1
RAD50
PMS1
FAN1
TP53
UPF1
XPC
NHEJ1
BAK1
DCLRE1C
BRCA2
SHMT2
MTHFD2
CDKN1A
SMG1
FOXO3
CEBPG
RASSF1
GADD45A
CBS
DDIT3
PPP1R15A
CTH
EDNRB
DHFR
DDB2
PNKP
LIG1
NNMT
APTX
USP7
TET3
MTRR
SMC5
TDG
DYRK2
NBN
ASF1A
CCNO
TRIAP1
GART
ATIC
SSRP1
FANCF
MECP2
MLH1
FANCE
FHIT
APOBEC1
ZAK
SMUG1
MTHFD2L
DNMT3B
SFN
FOLR1
RIPK2
CD14
TGFBR1
MYOD1
FABP3
FOLR2
PTEN
BHMT
APOBEC2
AMT
DNMT3A
BCL2
BTG2
TCN2
FOLH1
CDKN2B
TP73
CASP5
AICDA
FOLR3
APOBEC3A
MTHFR
ESR1
PON1
MBD2
MAT2B
MUC2
ALDH1L1
DNMT3L
FTCD
BRCA1
TIPIN
CHEK1
GPC3
MTR
TERT
GATA1
PEMT
MBD3
MAT1A
MTHFS
SLC19A1
NME1
SESN1
BCL3
RARB
CDKN2A
GSTP1
CDKN1C
TIMP3
MYO5
DLC1
MBD4
RECQL
SMC3
RAD21
APOBEC3C
PAX5
ABCB1
THBS1
PLAU
IDH1
ALDH2
CD82
IDH2
MAP2K5
CASP6
TP53TG1
MGMT
NEIL3
GTSE1
CCNA2
CCNB1
PTTG1
MAT2A
CHAF1A
HMGB2
SHMT1
AHCY
DNMT1
POLA1
PCNA
TYMS
MTHFD1
RPMI-8226
OVCAR-3
HS-578T
K-562
U251
BT-549
SK-MEL-2
NCI-H322M
A549
SKOV3
T-47D
CAKI-1
MDA-MB-468
DU-145
NCI-H23
UACC-257
786-0
MOLT-4
HCT-116
IGR-OV1
CCRF-CEM
M14
SNB-75
PC-3
SF-268
SK-MEL-5
TK-10
MALME-3M
SN12C
HOP-92
UO-31
SK-MEL-28
RXF-393
UACC-62
KM12
MCF7
OVCAR-4
MDA-MB-435
SF-295

Fig. S1

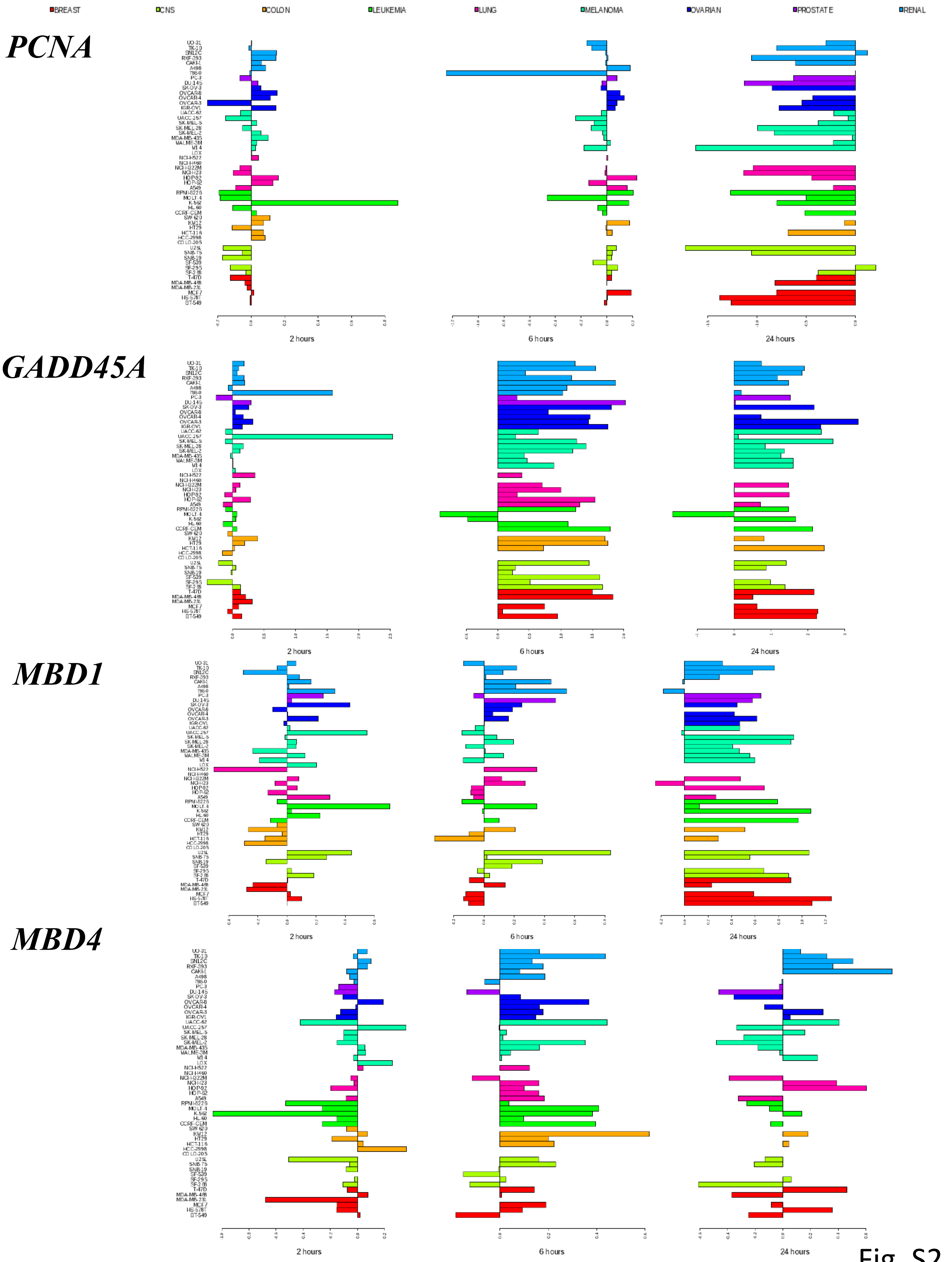

BREAST
CNS
COLON
LEUKEMIA
LUNG
MELANOMA
OVARIAN
PROSTATE
RENAL
PCNA
GADD45A
MBD1
MBD4
2 hours
6 hours
24 hours

Fig. S2.

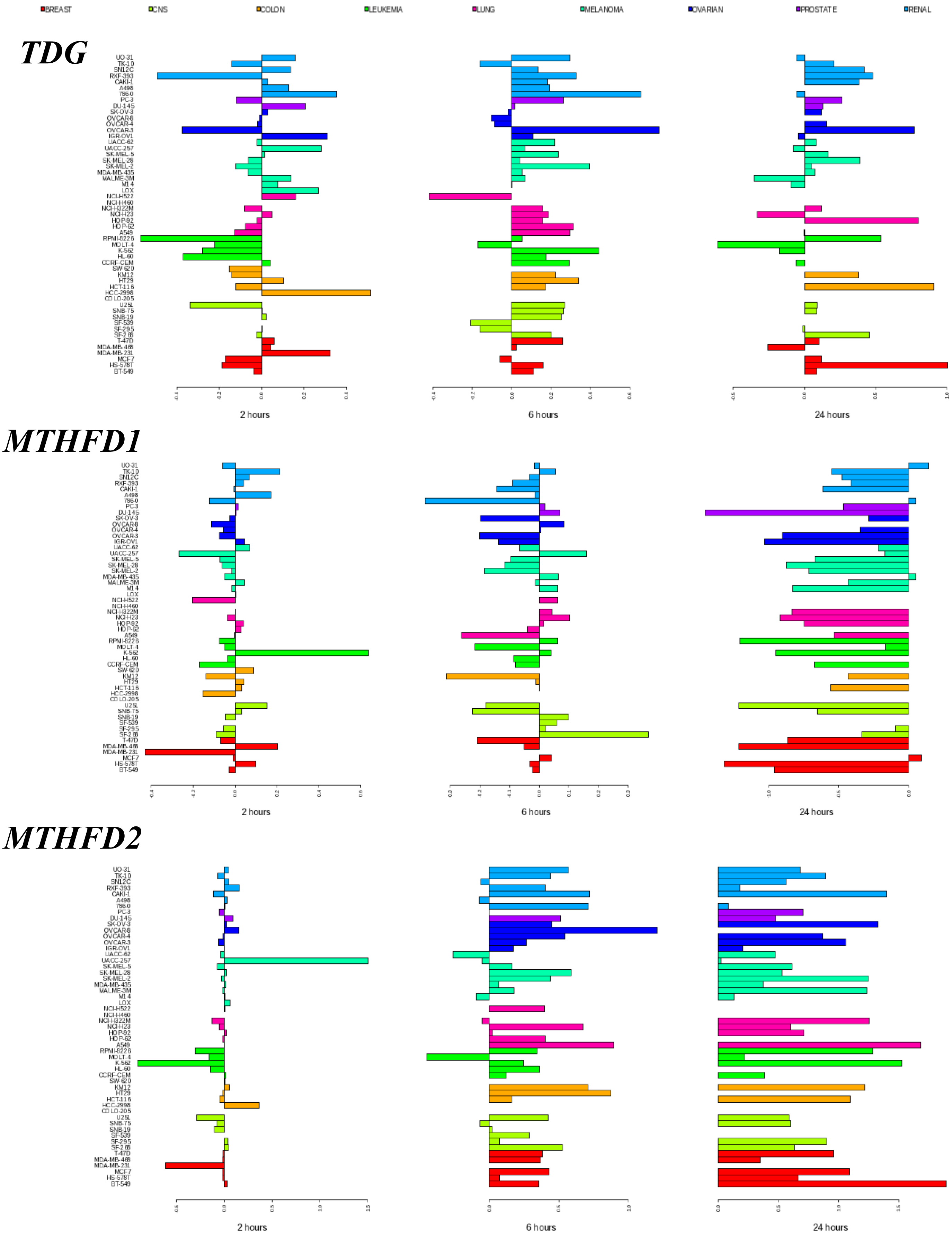
BREAST
CNS
COLON
LEUKEMIA
LUNG
MELANOMA
OVARIAN
PROSTATE
RENAL
TDG
MTHFD1
MTHFD2
UO-31
TK-10
SN12C
RXF-393
CAKI-1
A498
786-0
PC-3
DU-145
SK-OV-3
OVCAR-8
OVCAR-4
OVCAR-3
IGR-OV1
UACC-62
UACC-257
SK-MEL-5
SK-MEL-28
SK-MEL-2
MDA-MB-435
MALME-3M
M14
LOX
NCI-H522
NCI-H460
NCI-H322M
NCI-H23
HOP-92
HOP-62
A549
RPMI-8226
MOLT-4
K-562
HL-60
CCRF-CEM
SW-620
KM12
HT29
HCT-116
HCC-2998
COLO 205
U251
SNB-75
SNB-19
SF-539
SF-295
SF-268
T-47D
MDA-MB-468
MDA-MB-231
MCF7
HS 578T
BT-549
2 hours
6 hours
24 hours

Fig. S2. (cont.)

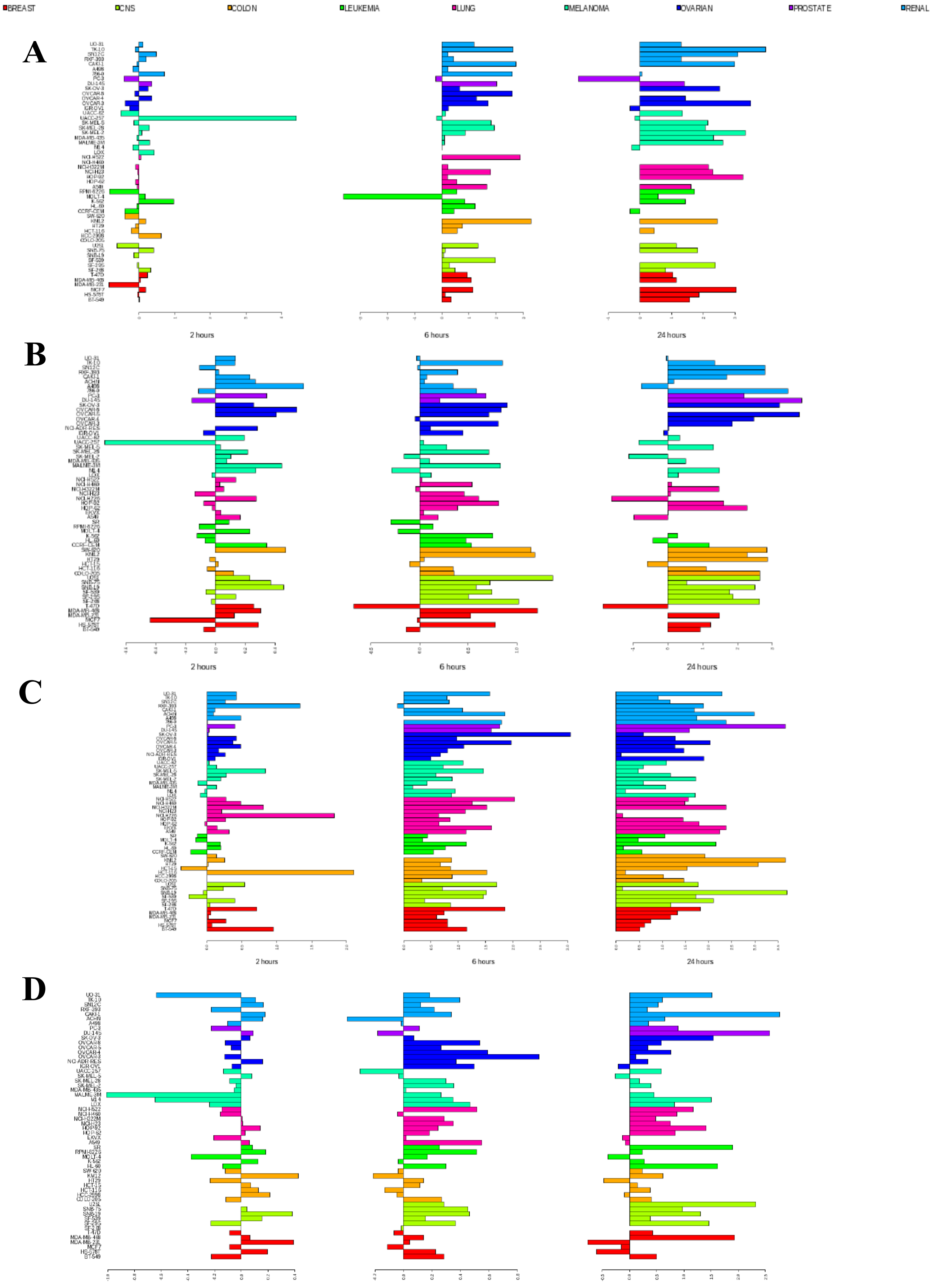
BREAST
CNS
COLON
LEUKEMIA
LUNG
MELANOMA
OVARIAN
PROSTATE
RENAL
A
2 hours
6 hours
24 hours
B
2 hours
6 hours
24 hours
C
2 hours
6 hours
24 hours
D
2 hours
6 hours
24 hours

Fig. S3.

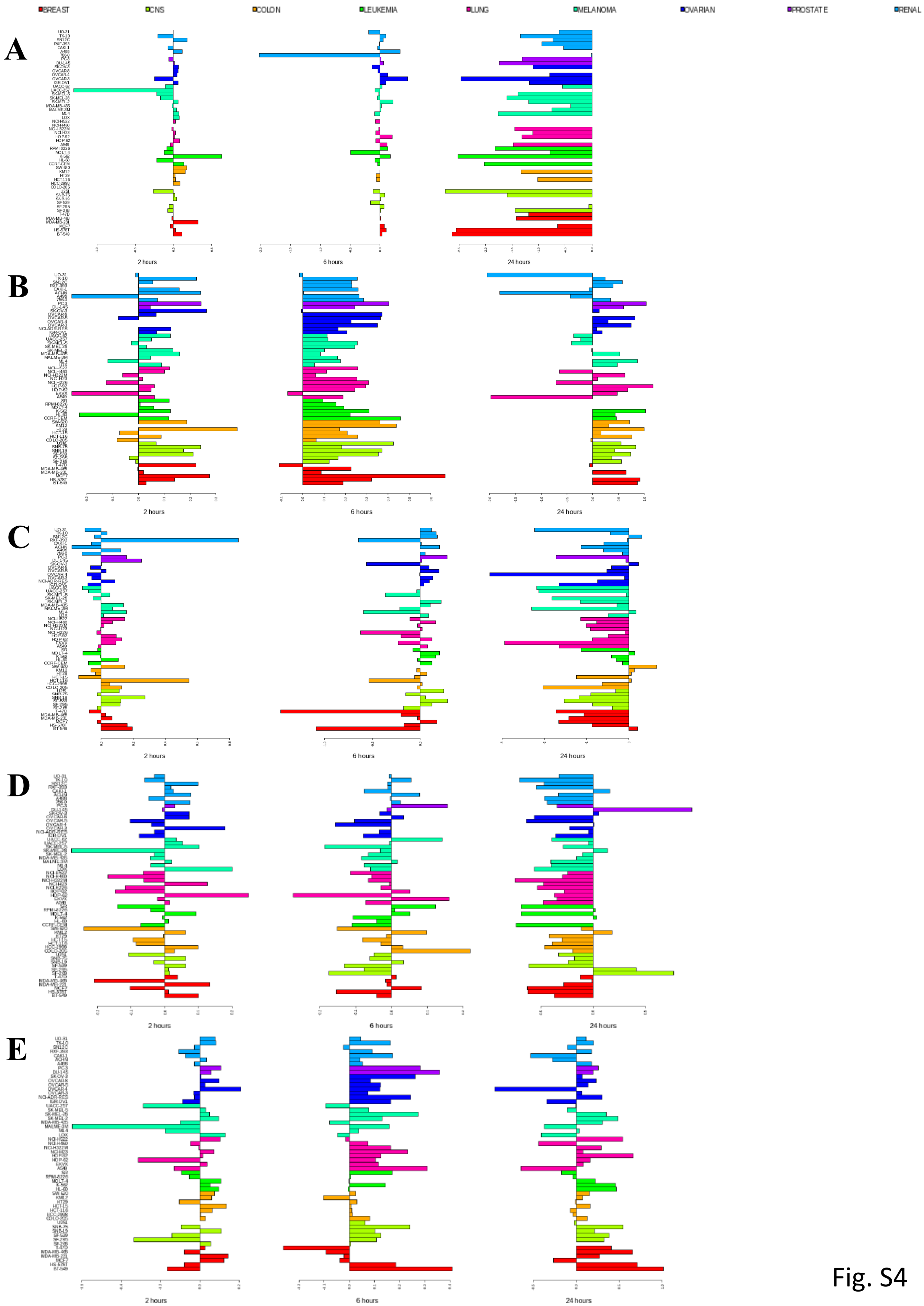
BREAST
CNS
COLON
LEUKEMIA
LUNG
MELANOMA
OVARIAN
PROSTATE
RENAL
A
B
C
D
E
2 hours
6 hours
24 hours

Fig. S4

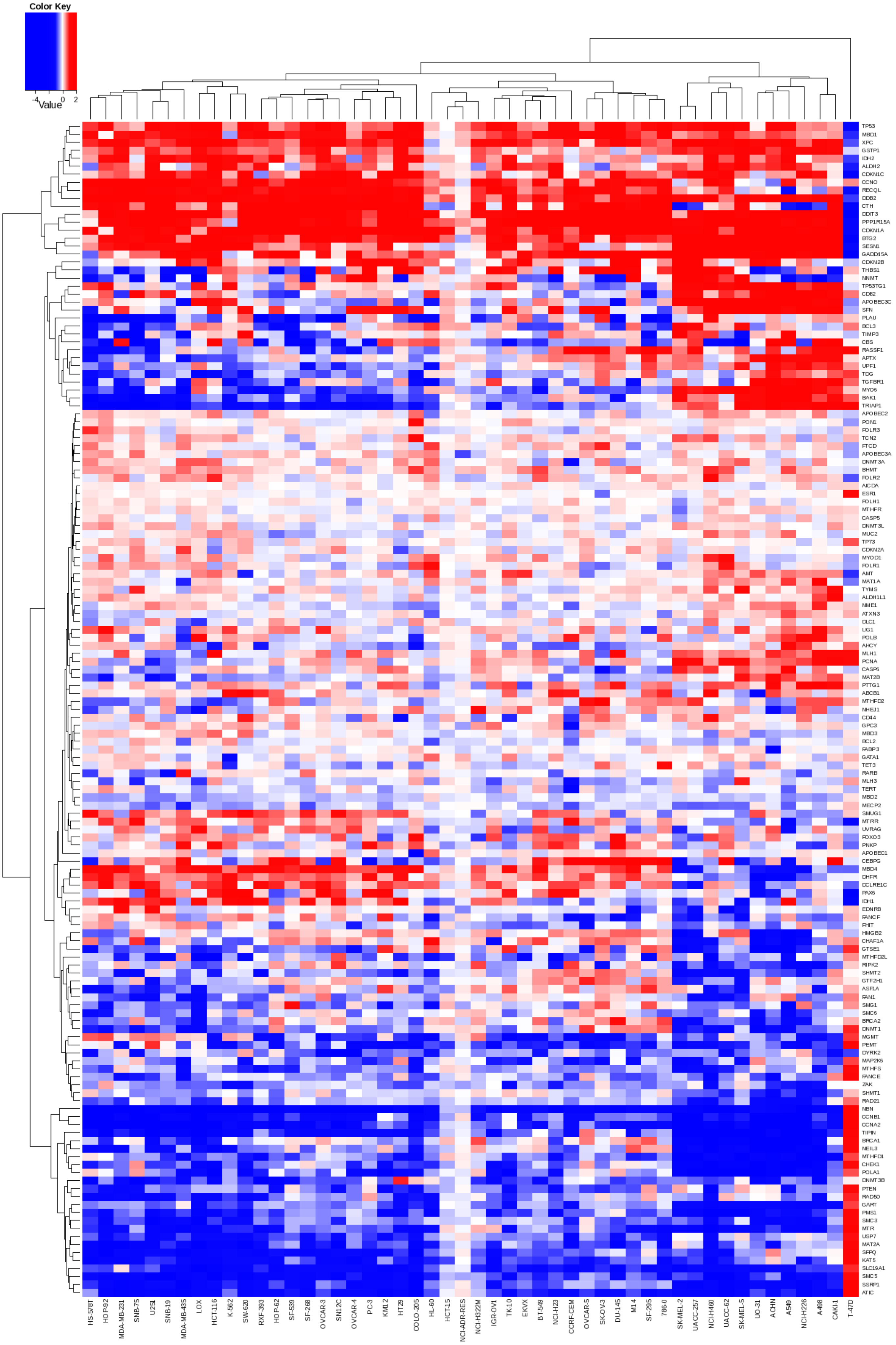
Color Key
-4
0
2
Value
TP53
MBD1
XPC
GSTP1
IDH2
ALDH2
CDKN1C
CCNO
RECQL
DDB2
CTH
DDIT3
PPP1R15A
CDKN1A
BTG2
SESN1
GADD45A
CDKN2B
THBS1
NNMT
TP53TG1
CD82
APOBEC3C
SFN
PLAU
BCL3
TIMP3
CBS
RASSF1
APTX
UPF1
TDG
TGFBR1
MYO5
BAK1
TRIAP1
APOBEC2
PON1
FOLR3
TCN2
FTCD
APOBEC3A
DNMT3A
BHMT
FOLR2
AICDA
ESR1
FOLH1
MTHFR
CASP5
DNMT3L
MUC2
TP73
CDKN2A
MYOD1
FOLR1
AMT
MAT1A
TYMS
ALDH1L1
NME1
ATXN3
DLC1
LIG1
POLB
AHCY
MLH1
PCNA
CASP6
MAT2B
PTTG1
ABCB1
MTHFD2
NHEJ1
CD14
GPC3
MBD3
BCL2
FABP3
GATA1
TET3
RARB
MLH3
TERT
MBD2
MECP2
SMUG1
MTRR
UVRAG
FOXO3
PNKP
APOBEC1
CEBPG
MBD4
DHFR
DCLRE1C
PAX6
IDH1
EDNRB
FANCF
FHIT
HMGB2
CHAF1A
GTSE1
MTHFD2L
RIPK2
SHMT2
GTF2H1
ASF1A
FAN1
SMG1
SMC6
BRCA2
DNMT1
MGMT
PEMT
DYRK2
MAP2K5
MTHFS
FANCE
ZAK
SHMT1
RAD21
NBN
CCNB1
CCNA2
TIPIN
BRCA1
NEIL3
MTHFD1
CHEK1
POLA1
DNMT3B
PTEN
RAD50
GART
PMS1
SMC3
MTR
USP7
MAT2A
SFPQ
KAT5
SLC19A1
SMC5
SSRP1
ATIC
HS-578T
HOP-92
MDA-MB-231
SNB-75
U251
SNB-19
MDA-MB-435
LOX
HCT-116
K-562
SW-620
RXF-393
HOP-62
SF-539
SF-268
OVCAR-3
SN12C
OVCAR-4
PC-3
KM12
HT29
COLO-205
HL-60
HCT-15
NCI-ADR-RES
NCI-H322M
IGR-OV1
TK-10
EKVX
BT-549
NCI-H23
CCRF-CEM
OVCAR-5
SK-OV-3
DU-145
M14
SF-295
786-0
SK-MEL-2
UACC-257
NCI-H460
UACC-62
SK-MEL-5
UO-31
ACHN
A549
NCI-H226
A498
CAKI-1
T-47D

Fig. S5

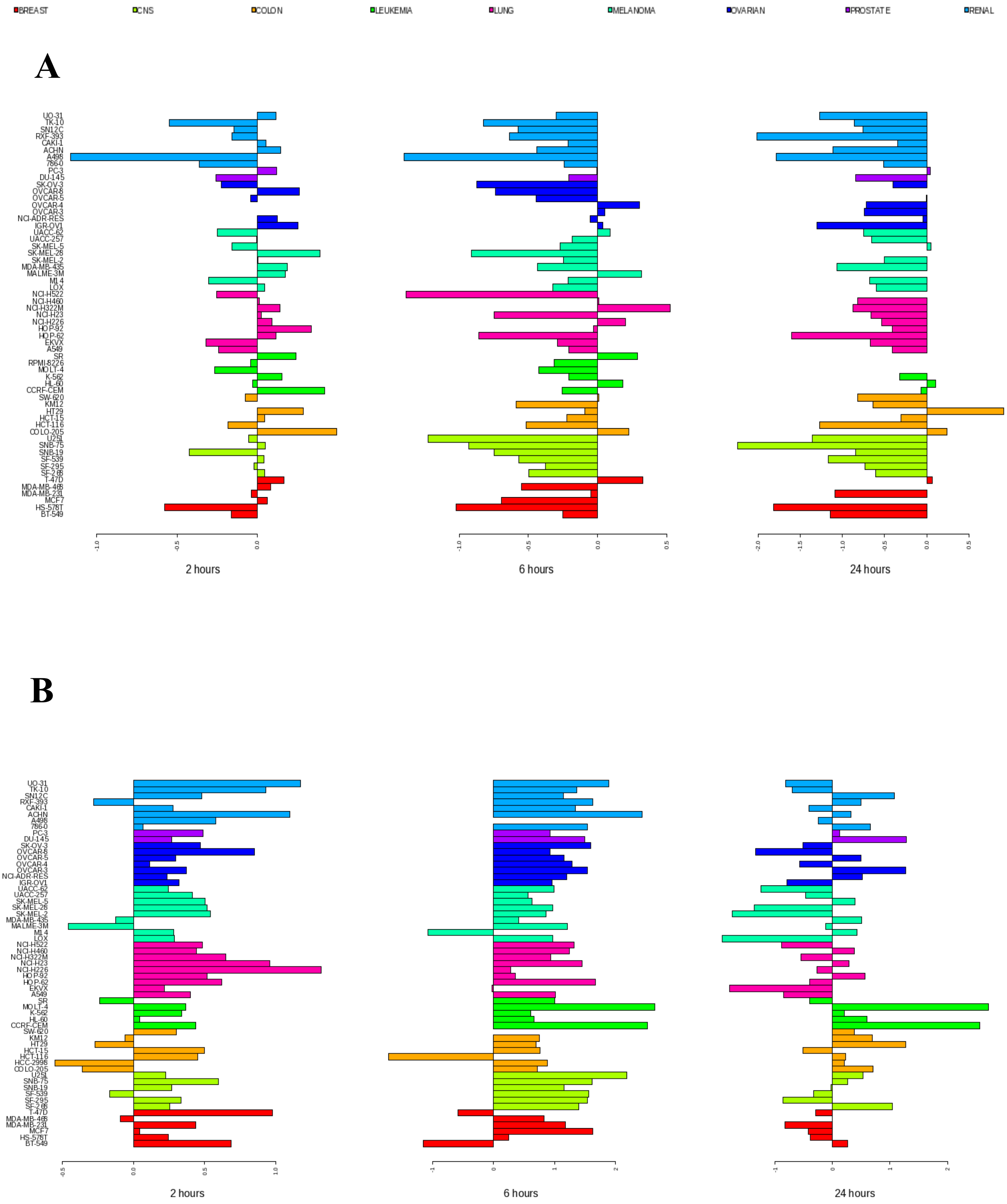
BREAST
CNS
COLON
LEUKEMIA
LUNG
MELANOMA
OVARIAN
PROSTATE
RENAL
A
UO-31
TK-10
SN12C
RXF-393
CAKI-1
ACHN
A498
786-0
PC-3
DU-145
SK-OV-3
OVCAR-8
OVCAR-5
OVCAR-4
OVCAR-3
NCI-ADR-RES
IGR-OV1
UACC-62
UACC-257
SK-MEL-5
SK-MEL-28
SK-MEL-2
MDA-MB-435
MALME-3M
M14
LOX
NCI-H522
NCI-H460
NCI-H322M
NCI-H23
NCI-H226
HOP-92
HOP-62
EKVX
A549
SR
RPMI-8226
MOLT-4
K-562
HL-60
CCRF-CEM
SW-620
KM12
HT29
HCT-15
HCT-116
COLO 205
U251
SNB-75
SNB-19
SF-539
SF-295
SF-268
T-47D
MDA-MB-468
MDA-MB-231
MCF7
HS-578T
BT-549
2 hours
6 hours
24 hours
B
UO-31
TK-10
SN12C
RXF-393
CAKI-1
ACHN
A498
786-0
PC-3
DU-145
SK-OV-3
OVCAR-8
OVCAR-5
OVCAR-4
OVCAR-3
NCI-ADR-RES
IGR-OV1
UACC-62
UACC-257
SK-MEL-5
SK-MEL-28
SK-MEL-2
MDA-MB-435
MALME-3M
M14
LOX
NCI-H522
NCI-H460
NCI-H322M
NCI-H23
NCI-H226
HOP-92
HOP-62
EKVX
A549
SR
MOLT-4
K-562
HL-60
CCRF-CEM
SW-620
KM12
HT29
HCT-15
HCT-116
HCC-2998
COLO 205
U251
SNB-75
SNB-19
SF-539
SF-295
SF-268
T-47D
MDA-MB-468
MDA-MB-231
MCF7
HS-578T
BT-549
2 hours
6 hours
24 hours

Fig. S6

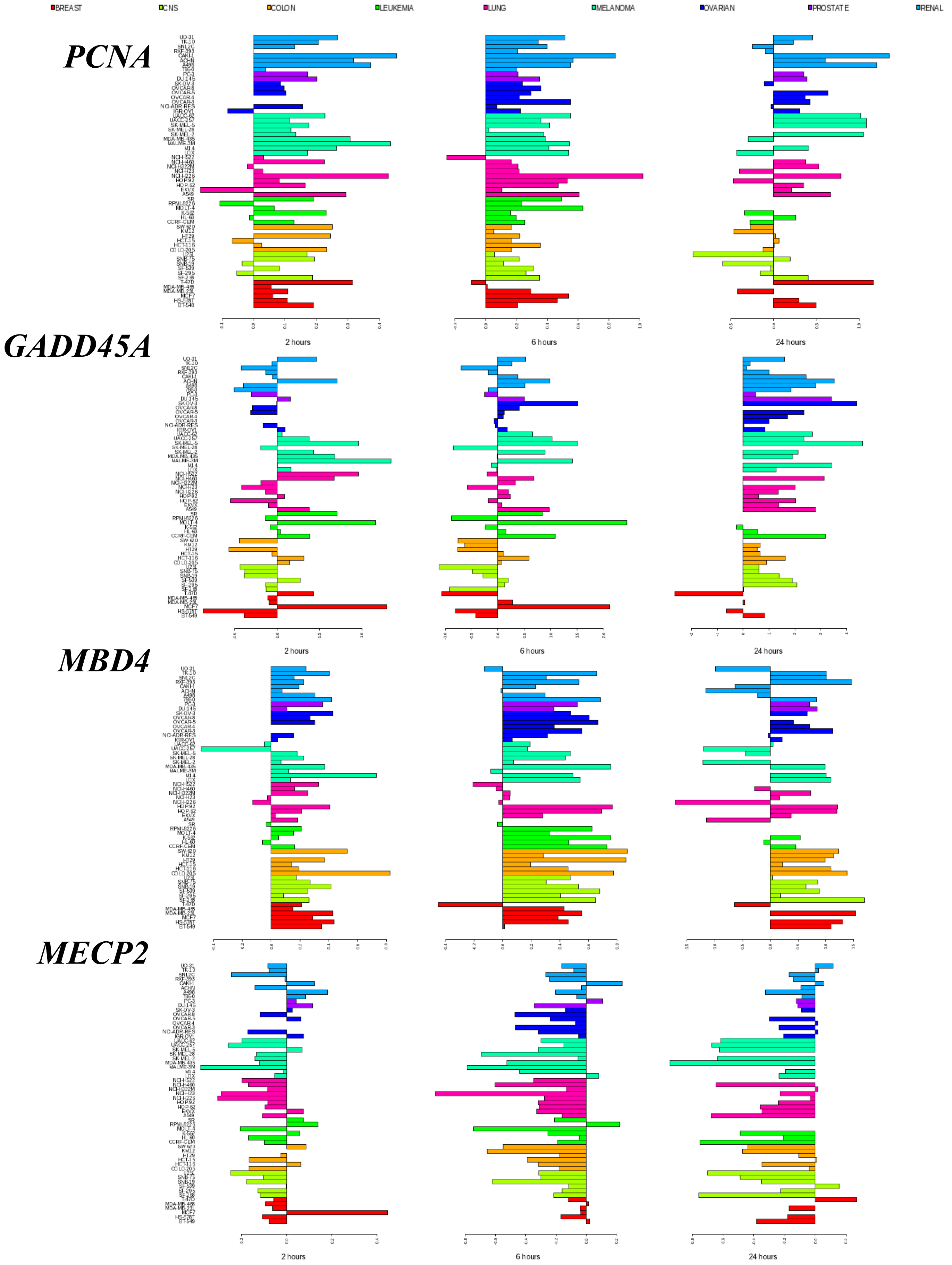
BREAST
CNS
COLON
LEUKEMIA
LUNG
MELANOMA
OVARIAN
PROSTATE
RENAL
PCNA
GADD45A
MBD4
MECP2
2 hours
6 hours
24 hours

Fig. S7

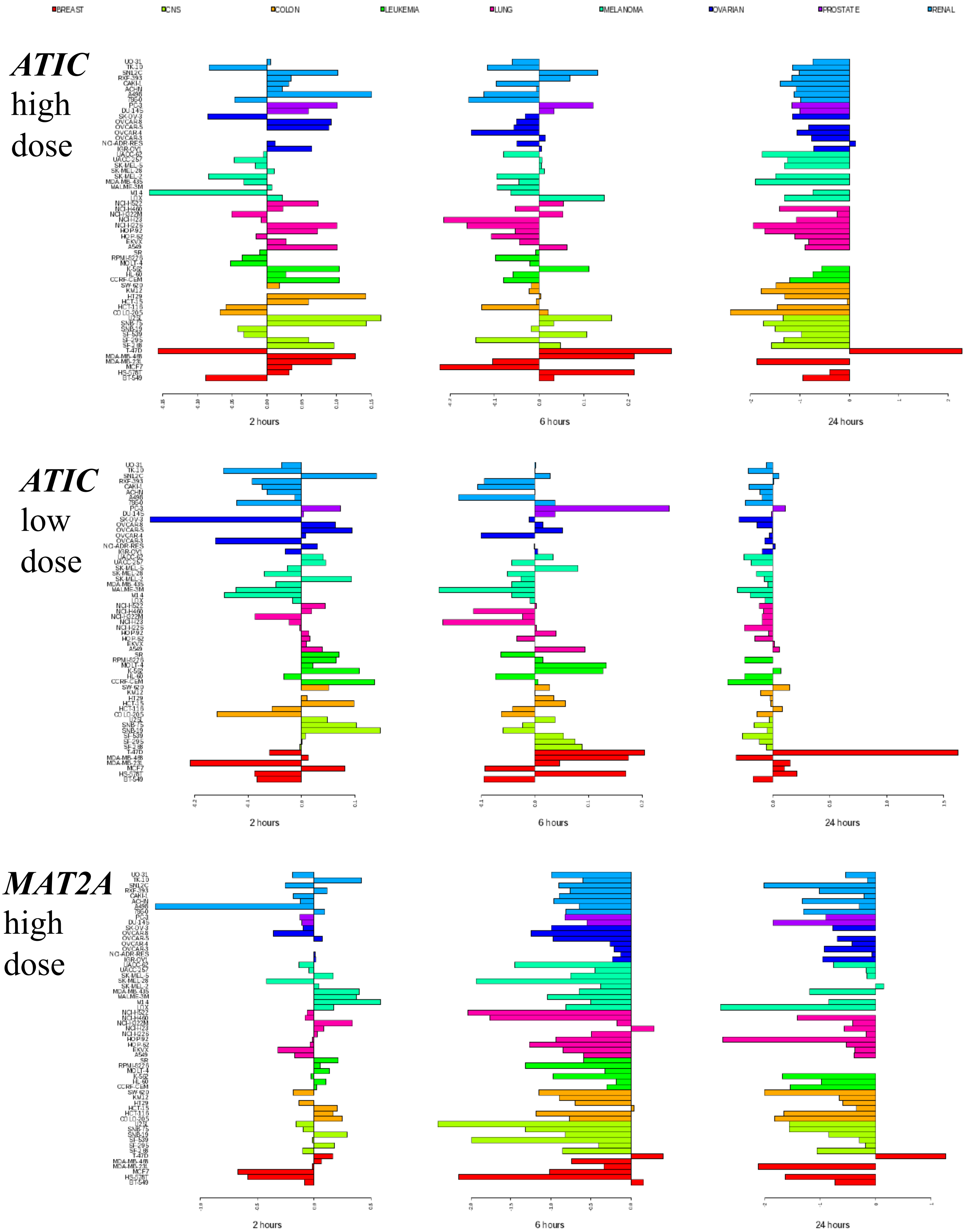
BREAST
CNS
COLON
LEUKEMIA
LUNG
MELANOMA
OVARIAN
PROSTATE
RENAL
ATIC high dose
ATIC low dose
MAT2A high dose
2 hours
6 hours
24 hours

Fig. S8

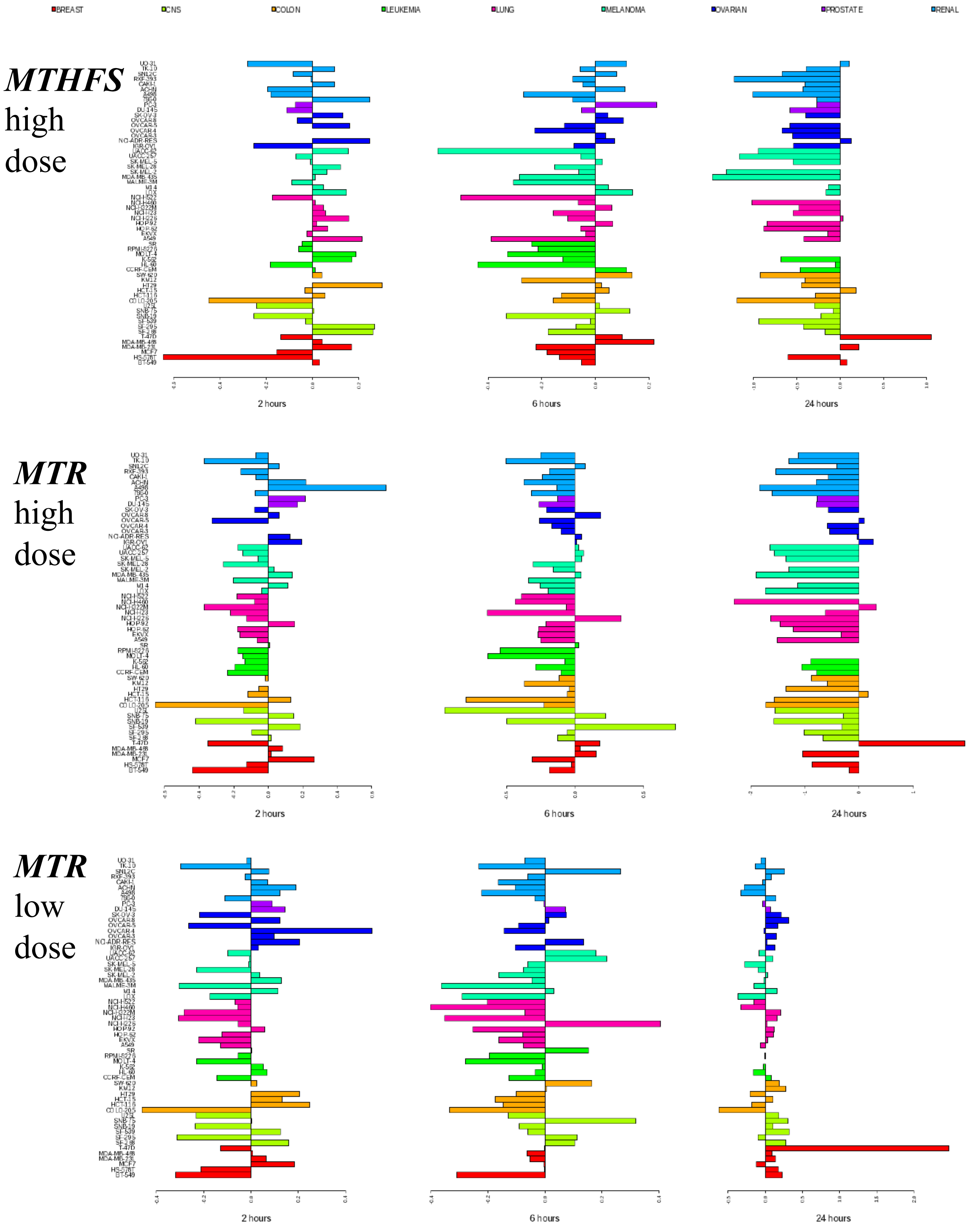
BREAST
CNS
COLON
LEUKEMIA
LUNG
MELANOMA
OVARIAN
PROSTATE
RENAL
MTHFS
high
dose
MTR
high
dose
MTR
low
dose
2 hours
6 hours
24 hours

Fig. S8 (cont.)

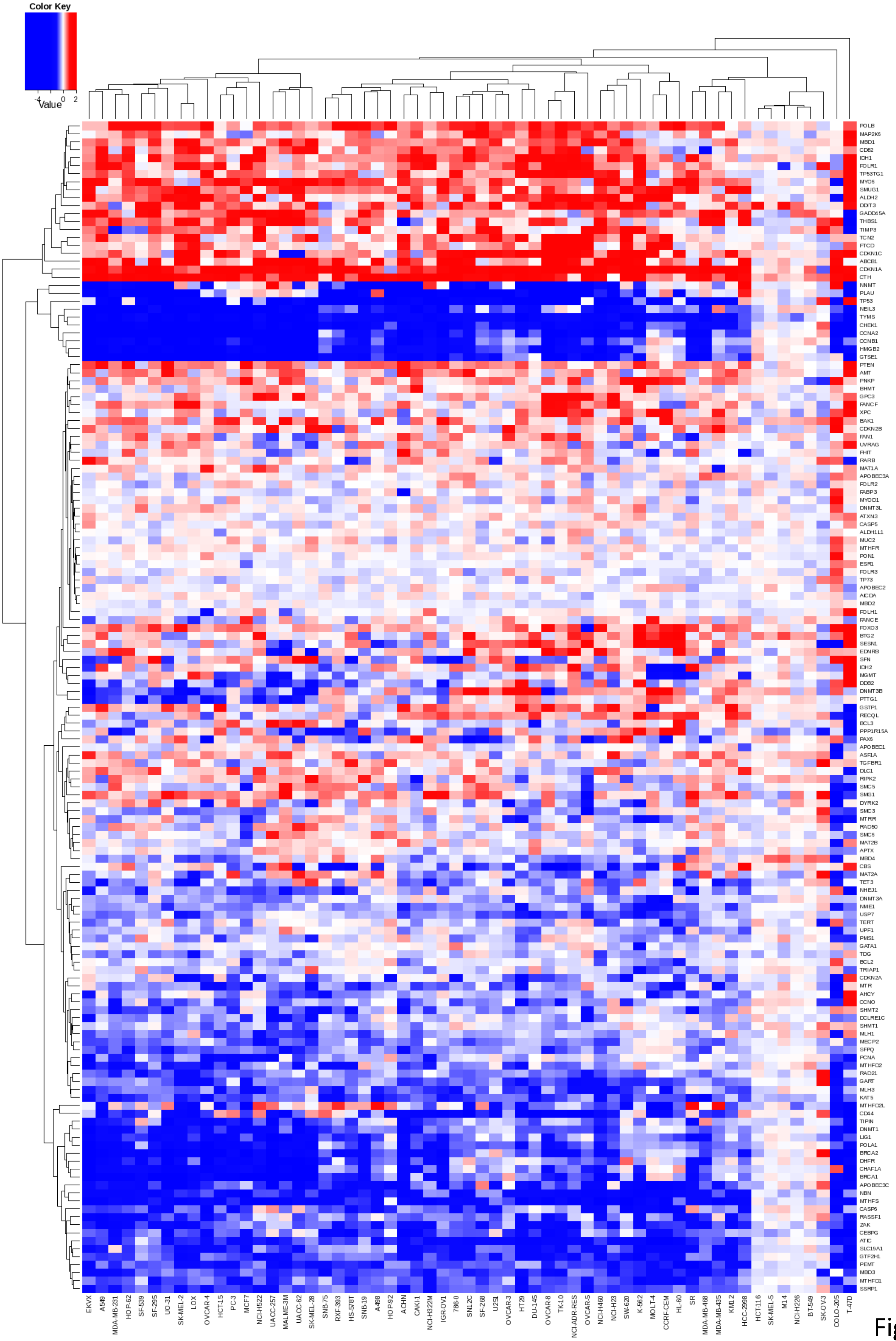
Color Key
Value
-4
0
2
EKVX
A549
MDA-MB-231
HOP-62
SF-539
SF-295
UO-31
SK-MEL-2
LOX
OVCAR-4
HCT-15
PC-3
MCF7
NCI-H522
UACC-257
MALME-3M
UACC-62
SK-MEL-28
SNB-75
RXF-393
HS-578T
SNB-19
A498
HOP-92
ACHN
CAKI-1
NCI-H322M
IGR-OV1
786-0
SN12C
SF-268
U251
OVCAR-3
HT29
DU-145
OVCAR-8
TK-10
NCI/ADR-RES
OVCAR-5
NCI-H460
NCI-H23
SW-620
K-562
MOLT-4
CCRF-CEM
HL-60
SR
MDA-MB-468
MDA-MB-435
KM12
HCC-2998
HCT116
SK-MEL-5
M14
NCI-H226
BT-549
SK-OV-3
COLO-205
T-47D
POLB
MAP2K5
MBD1
CD82
IDH1
FOLR1
TP53TG1
MYO5
SMUG1
ALDH2
DDIT3
GADD45A
THBS1
TIMP3
TCN2
FTCD
CDKN1C
ABCB1
CDKN1A
CTH
NNMT
PLAU
TP53
NEIL3
TYMS
CHEK1
CCNA2
CCNB1
HMGB2
GTSE1
PTEN
AMT
PNKP
BHMT
GPC3
FANCF
XPC
BAK1
CDKN2B
FAN1
UVRAG
FHIT
RARB
MAT1A
APOBEC3A
FOLR2
FABP3
MYOD1
DNMT3L
ATXN3
CASP5
ALDH1L1
MUC2
MTHFR
PON1
ESR1
FOLR3
TP73
APOBEC2
AICDA
MBD2
FOLH1
FANCE
FOXO3
BTG2
SESN1
EDNRB
SFN
IDH2
MGMT
DDB2
DNMT3B
PTTG1
GSTP1
RECQL
BCL3
PPP1R15A
PAX6
APOBEC1
ASF1A
TGFBR1
DLC1
RIPK2
SMC5
SMG1
DYRK2
SMC3
MTRR
RAD50
SMC6
MAT2B
APTX
MBD4
CBS
MAT2A
TET3
NHEJ1
DNMT3A
NME1
USP7
TERT
UPF1
PMS1
GATA1
TDG
BCL2
TRIAP1
CDKN2A
MTR
AHCY
CCNO
SHMT2
DCLRE1C
SHMT1
MLH1
MECP2
SFPQ
PCNA
MTHFD2
RAD21
GART
MLH3
KAT5
MTHFD2L
CD44
TIPIN
DNMT1
LIG1
POLA1
BRCA2
DHFR
CHAF1A
BRCA1
APOBEC3C
NBN
MTHFS
CASP6
RASSF1
ZAK
CEBPG
ATIC
SLC19A1
GTF2H1
PEMT
MBD3
MTHFD1
SSRP1

Fig. S9

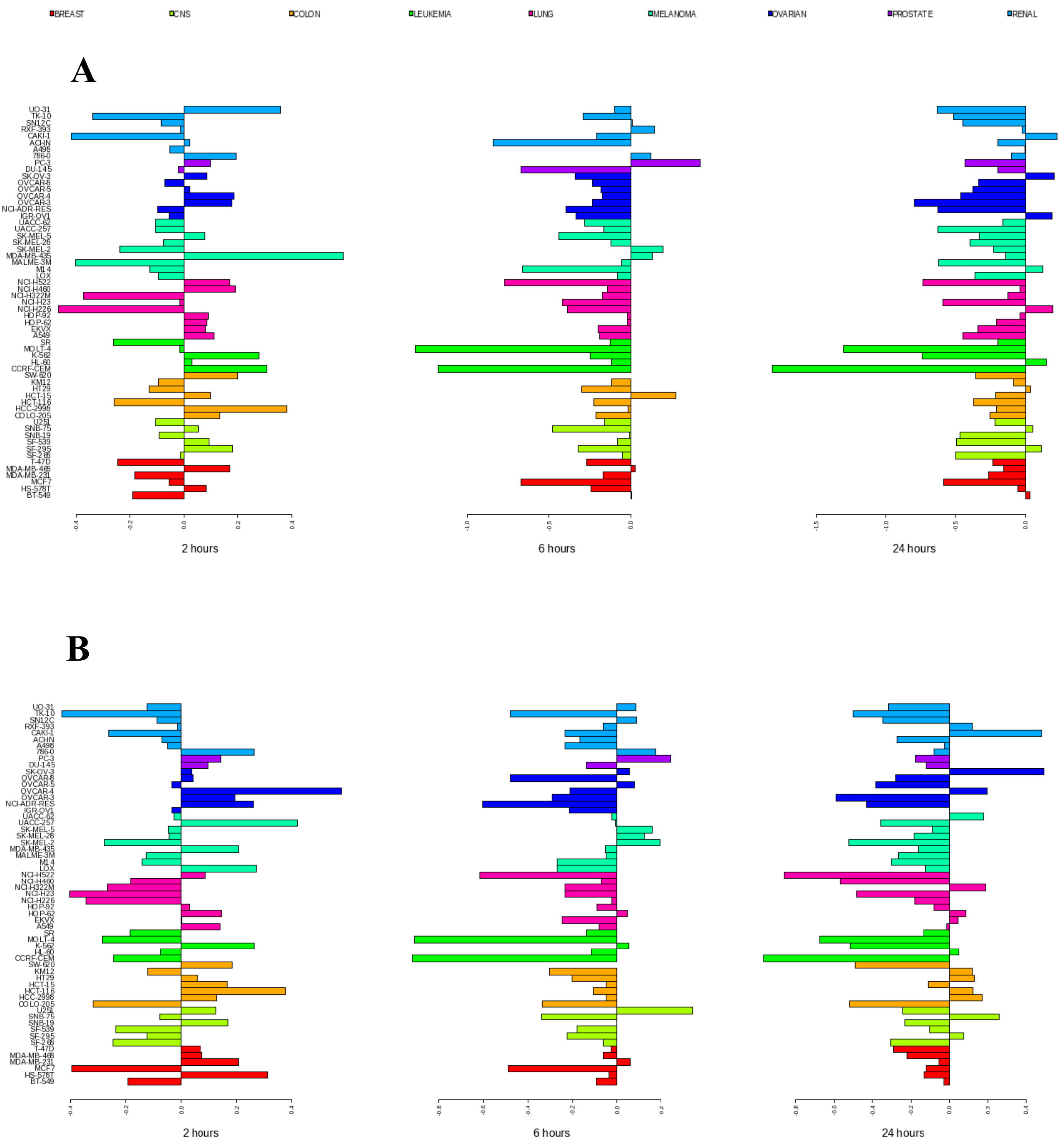

BREAST
CNS
COLON
LEUKEMIA
LUNG
MELANOMA
OVARIAN
PROSTATE
RENAL
A
2 hours
6 hours
24 hours
B
2 hours
6 hours
24 hours

Fig. S10

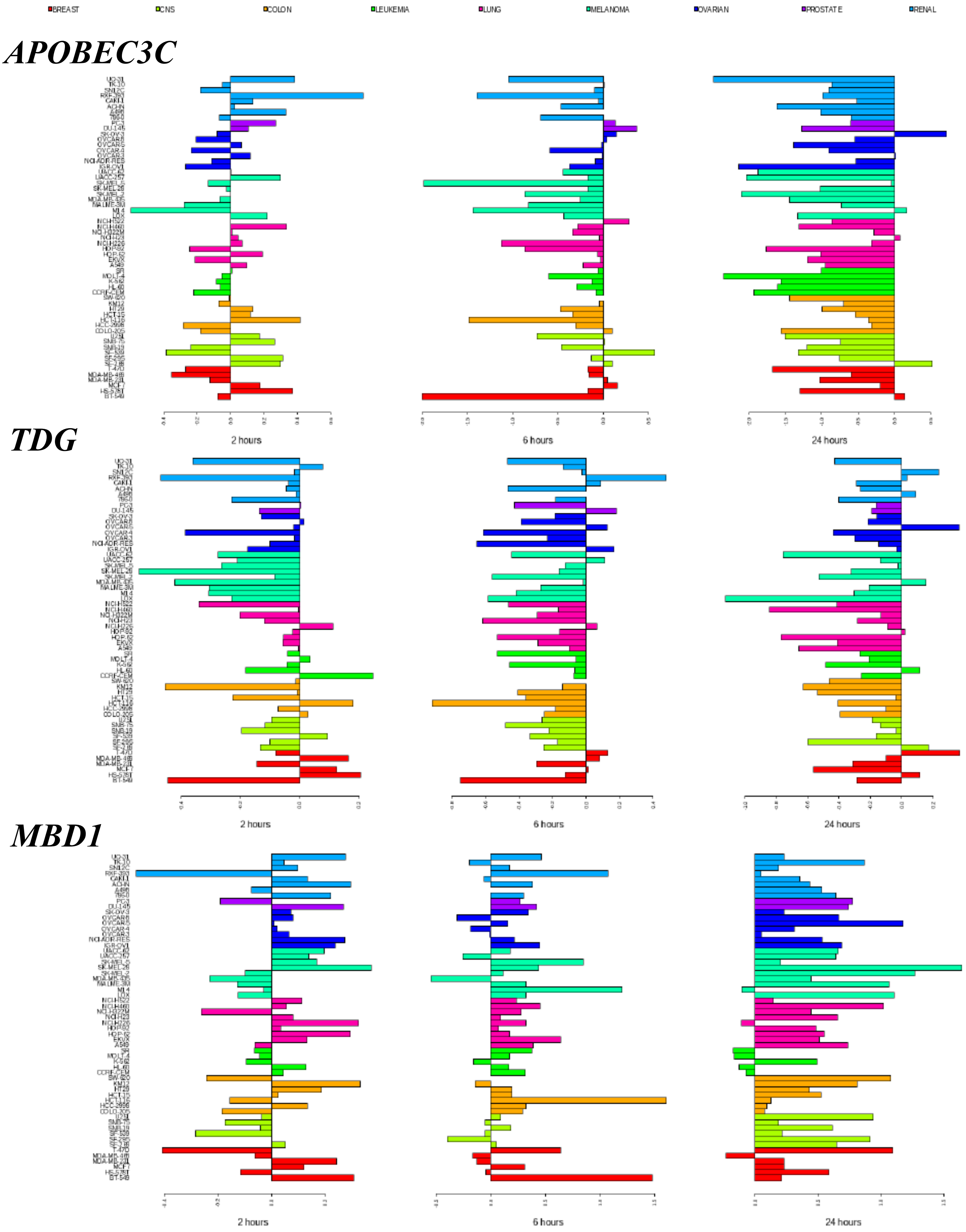

BREAST
CNS
COLON
LEUKEMIA
LUNG
MELANOMA
OVARIAN
PROSTATE
RENAL
APOBEC3C
2 hours
6 hours
24 hours
TDG
2 hours
6 hours
24 hours
MBD1
2 hours
6 hours
24 hours

Fig. S11

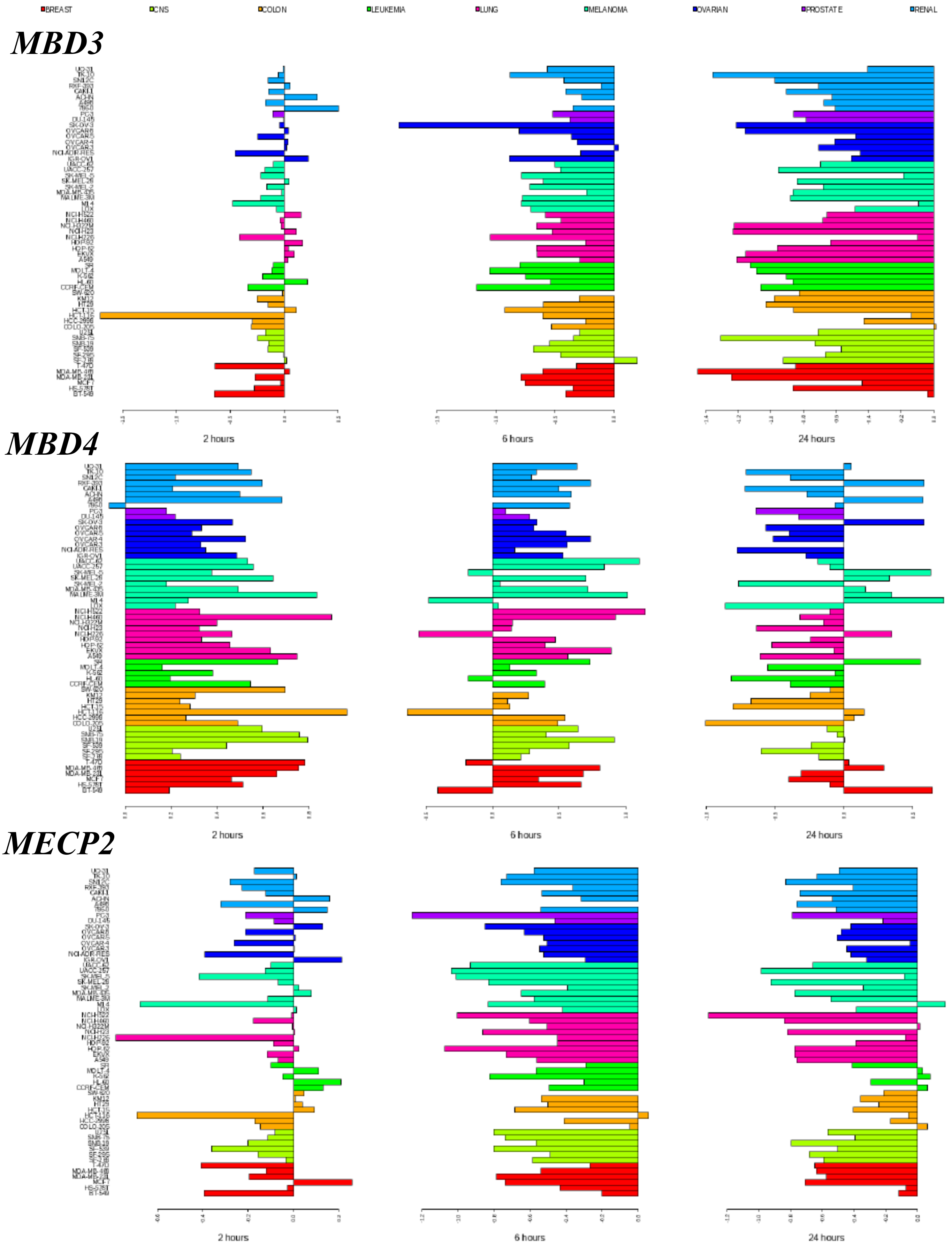
BREAST
CNS
COLON
LEUKEMIA
LUNG
MELANOMA
OVARIAN
PROSTATE
RENAL
MBD3
2 hours
6 hours
24 hours
MBD4
2 hours
6 hours
24 hours
MECP2
2 hours
6 hours
24 hours

Fig. S11 (cont.)

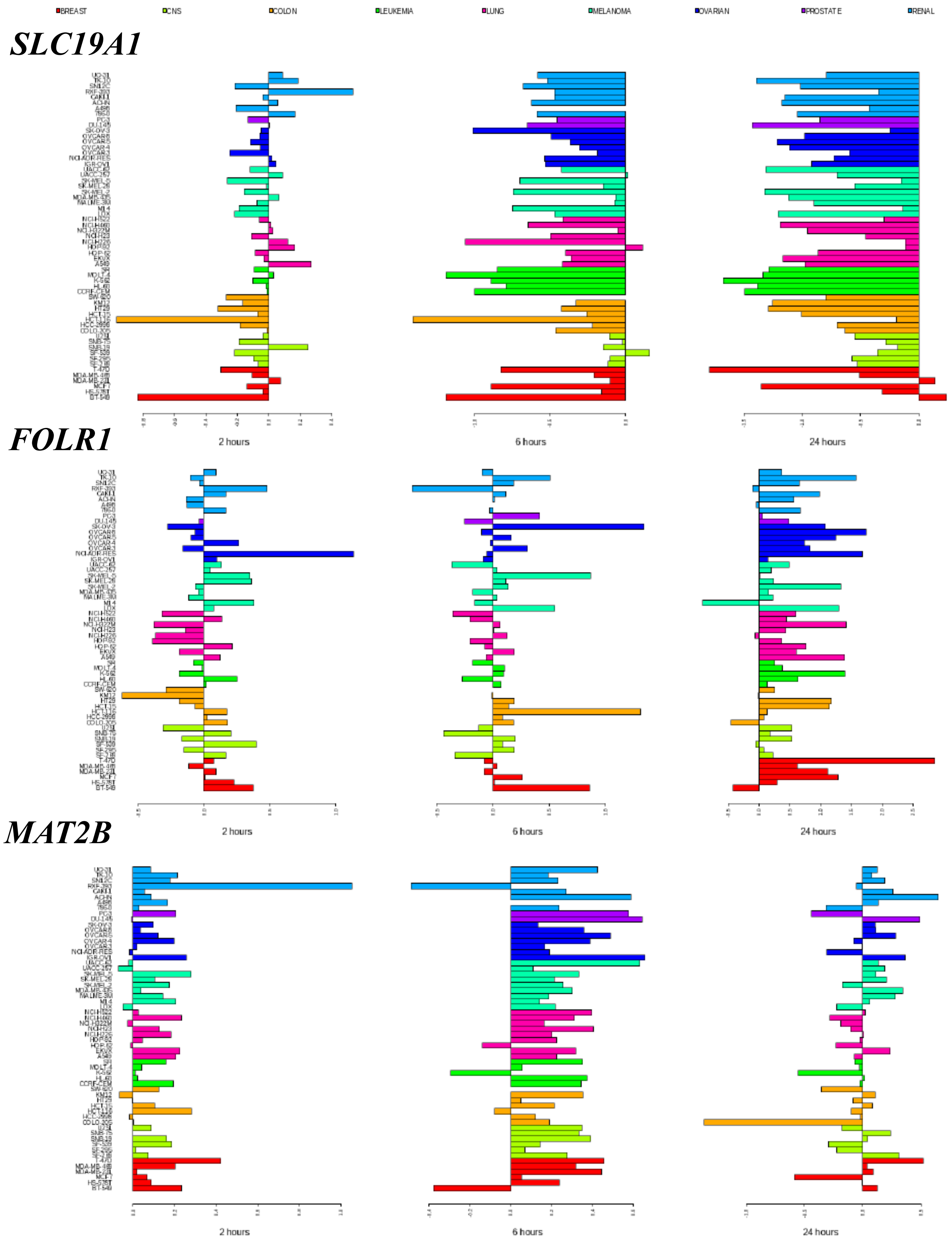
BREAST
CNS
COLON
LEUKEMIA
LUNG
MELANOMA
OVARIAN
PROSTATE
RENAL
SLC19A1
2 hours
6 hours
24 hours
FOLR1
2 hours
6 hours
24 hours
MAT2B
2 hours
6 hours
24 hours

Fig. S12

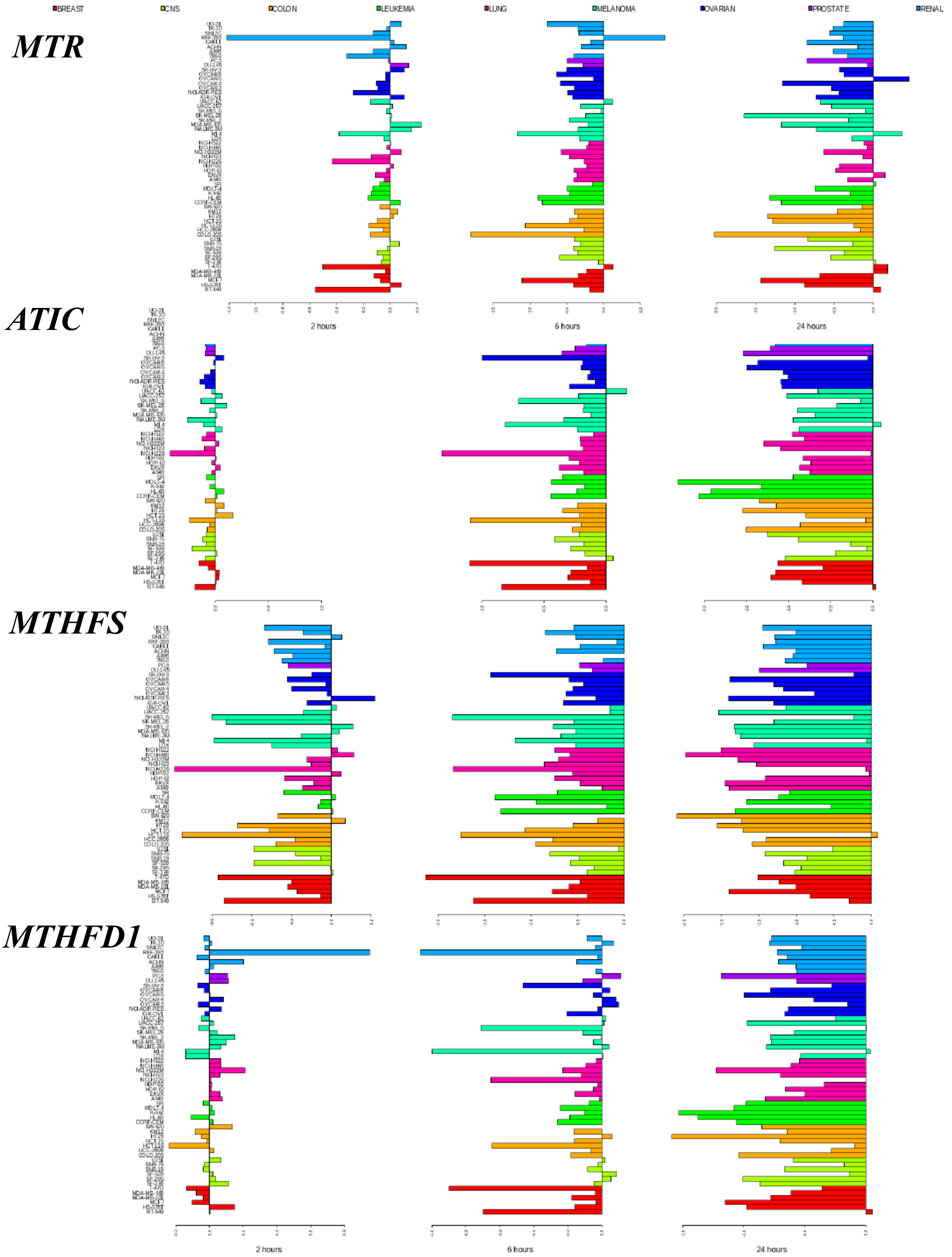

BREAST
CNS
COLON
LEUKEMIA
LUNG
MELANOMA
OVARIAN
PROSTATE
RENAL
MTR
ATIC
MTHFS
MTHFD1
2 hours
6 hours
24 hours

Fig. S12 (cont.)

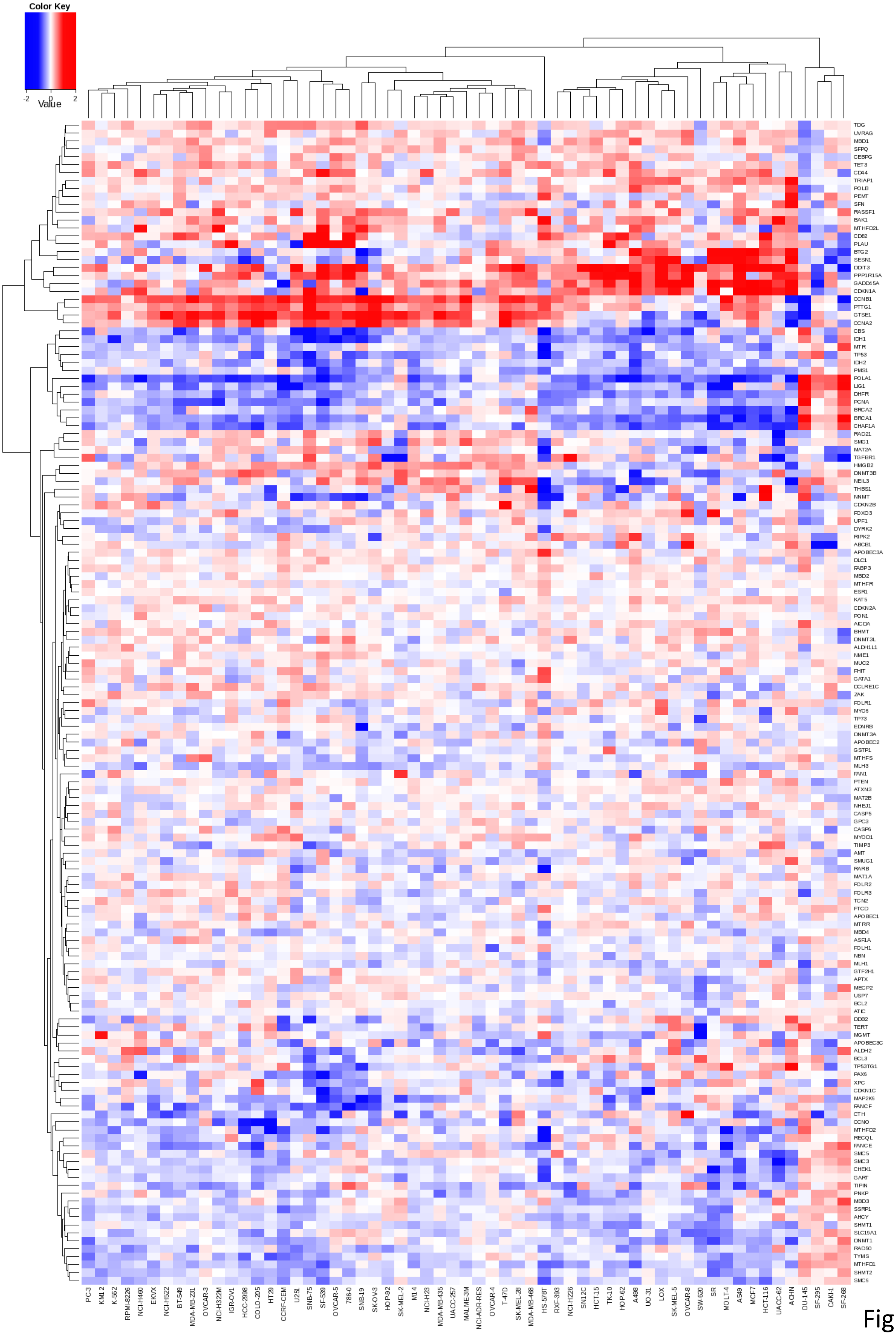
Color Key
-2
0
2
Value
TDG
UVRAG
MBD1
SFPQ
CEBPG
TET3
CD44
TRIAP1
POLB
PEMT
SFN
RASSF1
BAK1
MTHFD2L
CD82
PLAU
BTG2
SESN1
DDIT3
PPP1R15A
GADD45A
CDKN1A
CCNB1
PTTG1
GTSE1
CCNA2
CBS
IDH1
MTR
TP53
IDH2
PMS1
POLA1
LIG1
DHFR
PCNA
BRCA2
BRCA1
CHAF1A
RAD21
SMG1
MAT2A
TGFBR1
HMGB2
DNMT3B
NEIL3
THBS1
NNMT
CDKN2B
FOXO3
UPF1
DYRK2
RIPK2
ABCB1
APOBEC3A
DLC1
FABP3
MBD2
MTHFR
ESR1
KAT5
CDKN2A
PON1
AICDA
BHMT
DNMT3L
ALDH1L1
NME1
MUC2
FHIT
GATA1
DCLRE1C
ZAK
FOLR1
MYO5
TP73
EDNRB
DNMT3A
APOBEC2
GSTP1
MTHFS
MLH3
FAN1
PTEN
ATXN3
MAT2B
NHEJ1
CASP5
GPC3
CASP6
MYOD1
TIMP3
AMT
SMUG1
RARB
MAT1A
FOLR2
FOLR3
TCN2
FTCD
APOBEC1
MTRR
MBD4
ASF1A
FOLH1
NBN
MLH1
GTF2H1
APTX
MECP2
USP7
BCL2
ATIC
DDB2
TERT
MGMT
APOBEC3C
ALDH2
BCL3
TP53TG1
PAX5
XPC
CDKN1C
MAP2K6
FANCF
CTH
CCNO
MTHFD2
RECQL
FANCE
SMC5
SMC3
CHEK1
GART
TIPIN
PNKP
MBD3
SSRP1
AHCY
SHMT1
SLC19A1
DNMT1
RAD50
TYMS
MTHFD1
SHMT2
SMC6
PC-3
KM12
K-562
RPMI-8226
NCI-H460
EKVX
NCI-H522
BT-549
MDA-MB-231
OVCAR-3
NCI-H322M
IGR-OV1
HCC-2998
COLO-205
HT29
CCRF-CEM
U251
SNB-75
SF-539
OVCAR-5
786-0
SNB-19
SK-OV-3
HOP-92
SK-MEL-2
M14
NCI-H23
MDA-MB-435
UACC-257
MALME-3M
NCI-ADR-RES
OVCAR-4
T-47D
SK-MEL-28
MDA-MB-468
HS-578T
RXF-393
NCI-H226
SN12C
HCT-15
TK-10
HOP-62
A498
UO-31
LOX
SK-MEL-5
OVCAR-8
SW-620
SR
MOLT-4
A549
MCF7
HCT-116
UACC-62
ACHN
DU-145
SF-295
CAKI-1
SF-268

Fig. S13

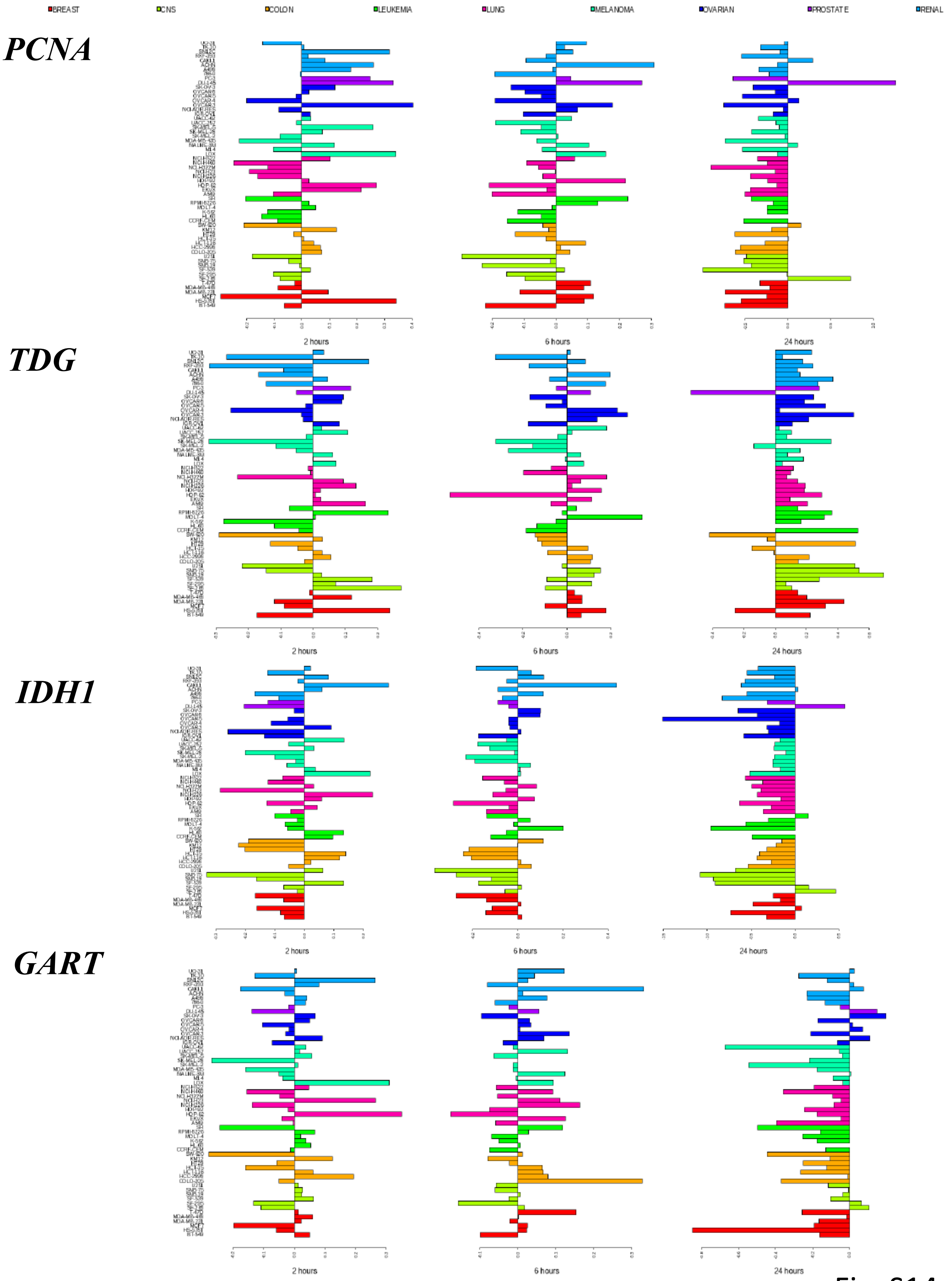
BREAST
CNS
COLON
LEUKEMIA
LUNG
MELANOMA
OVARIAN
PROSTATE
RENAL
PCNA
2 hours
6 hours
24 hours
TDG
2 hours
6 hours
24 hours
IDH1
2 hours
6 hours
24 hours
GART
2 hours
6 hours
24 hours

Fig. S14

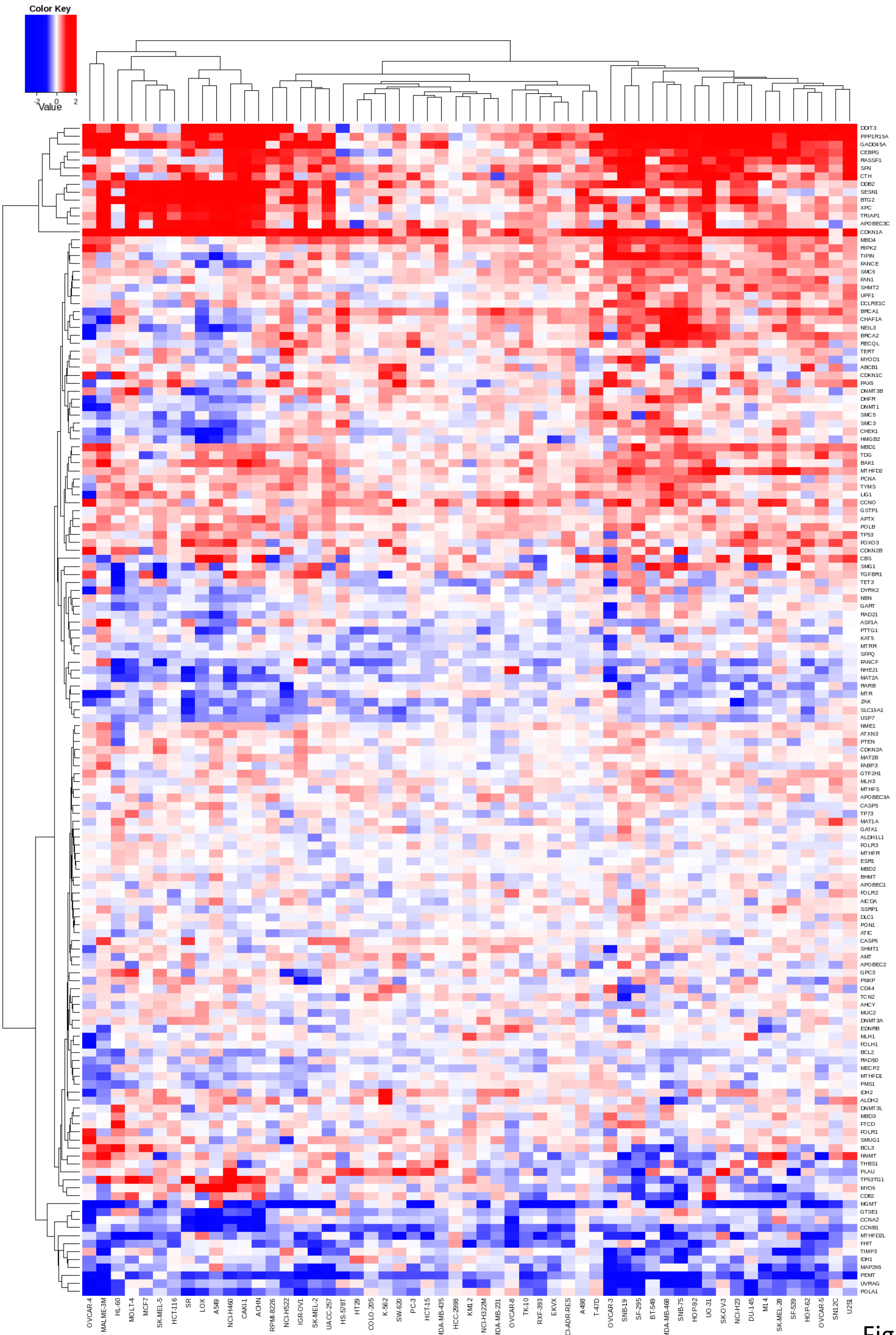
Color Key
-2
0
2
Value
DDIT3
PPP1R15A
GADD45A
CEBPG
RASSF1
SFN
CTH
DDB2
SESN1
BTG2
XPC
TRIAP1
APOBEC3C
CDKN1A
MBD4
RIPK2
TIPIN
FANCE
SMC6
FAN1
SHMT2
UPF1
DCLRE1C
BRCA1
CHAF1A
NEIL3
BRCA2
RECQL
TERT
MYOD1
ABCB1
CDKN1C
PAX5
DNMT3B
DHFR
DNMT1
SMC5
SMC3
CHEK1
HMGB2
MBD1
TDG
BAK1
MTHFD2
PCNA
TYMS
LIG1
CCNO
GSTP1
APTX
POLB
TP53
FOXO3
CDKN2B
CBS
SMG1
TGFBR1
TET3
DYRK2
NBN
GART
RAD21
ASF1A
PTTG1
KAT5
MTRR
SFPQ
FANCF
NHEJ1
MAT2A
RARB
MTR
ZAK
SLC19A1
USP7
NME1
ATXN3
PTEN
CDKN2A
MAT2B
FABP3
GTF2H1
MLH3
MTHFS
APOBEC3A
CASP5
TP73
MAT1A
GATA1
ALDH1L1
FOLR3
MTHFR
ESR1
MBD2
BHMT
APOBEC1
FOLR2
AICDA
SSRP1
DLC1
PON1
ATIC
CASP6
SHMT1
AMT
APOBEC2
GPC3
PNKP
CD14
TCN2
AHCY
MUC2
DNMT3A
EDNRB
MLH1
FOLH1
BCL2
RAD50
MECP2
MTHFD1
PMS1
IDH2
ALDH2
DNMT3L
MBD3
FTCD
FOLR1
SMUG1
BCL3
NNMT
THBS1
PLAU
TP53TG1
MYO5
CD82
MGMT
GTSE1
CCNA2
CCNB1
MTHFD2L
FHIT
TIMP3
IDH1
MAP2K5
PEMT
UVRAG
POLA1
OVCAR-4
MALME-3M
HL-60
MOLT-4
MCF7
SK-MEL-5
HCT-116
SR
LOX
A549
NCI-H460
CAKI-1
ACHN
RPMI-8226
NCI-H522
IGR-OV1
SK-MEL-2
UACC-257
HS-578T
HT29
COLO-205
K-562
SW-620
PC-3
HCT15
MDA-MB-435
HCC-2998
KM12
NCI-H322M
MDA-MB-231
OVCAR-8
TK-10
RXF-393
EKVX
NCI/ADR-RES
A498
T47D
OVCAR-3
SNB-19
SF-295
BT-549
MDA-MB-468
SNB-75
HOP-92
UO-31
SK-OV-3
NCI-H23
DU-145
M14
SK-MEL-28
SF-539
HOP-62
OVCAR-5
SN12C
U251

Fig. S15

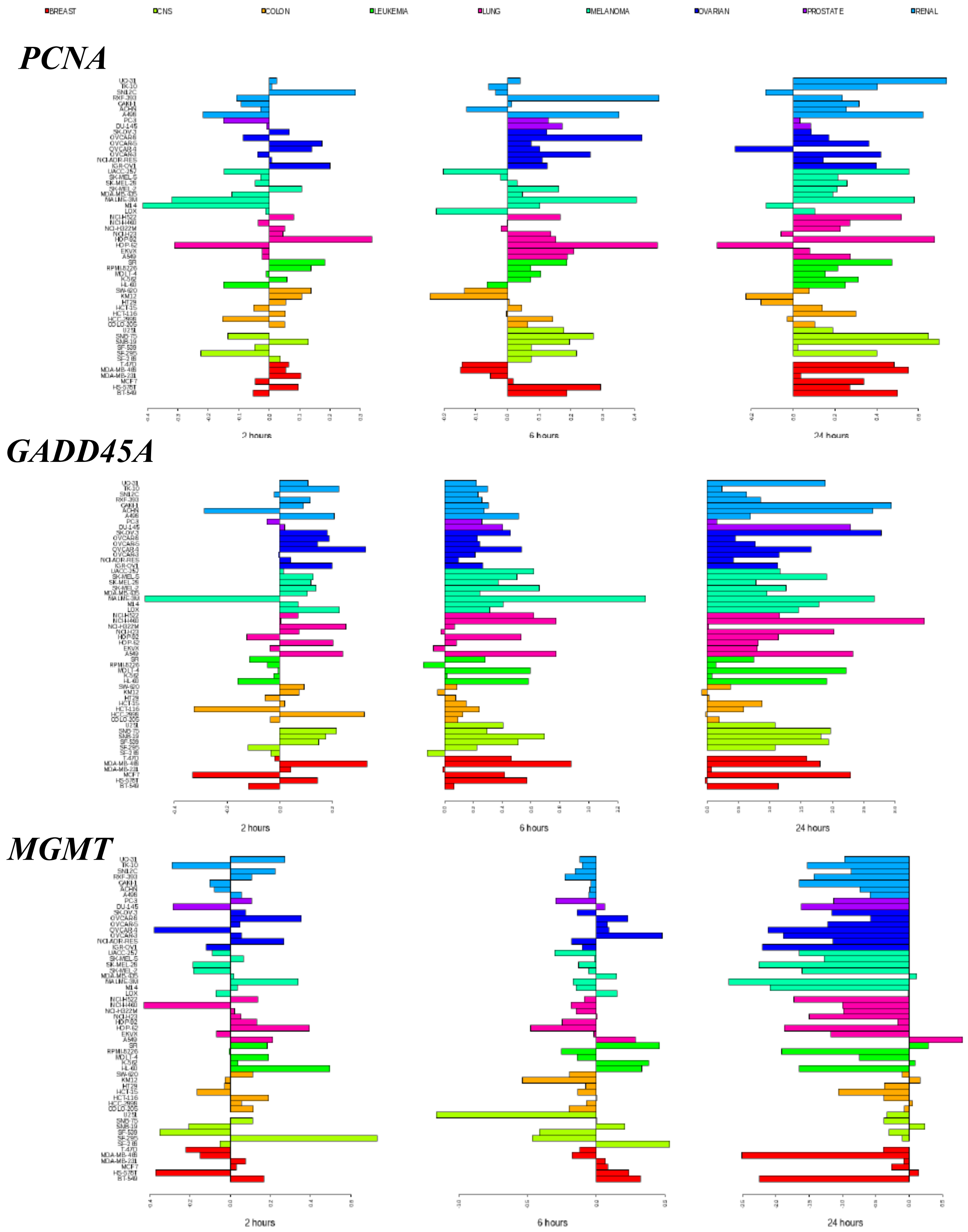
BREAST
CNS
COLON
LEUKEMIA
LUNG
MELANOMA
OVARIAN
PROSTATE
RENAL
PCNA
2 hours
6 hours
24 hours
GADD45A
2 hours
6 hours
24 hours
MGMT
2 hours
6 hours
24 hours

Fig. S16

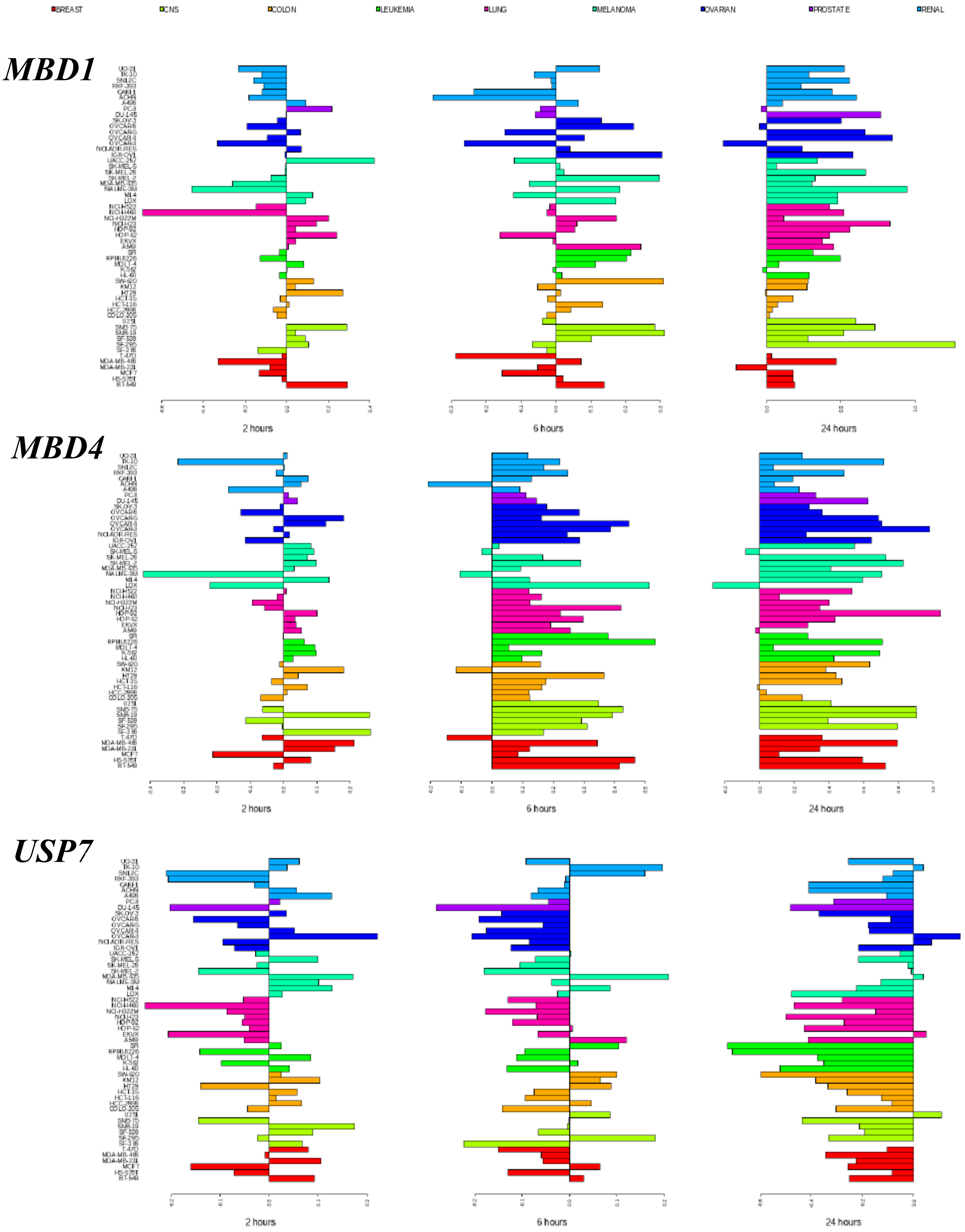
BREAST
CNS
COLON
LEUKEMIA
LUNG
MELANOMA
OVARIAN
PROSTATE
RENAL
MBD1
2 hours
6 hours
24 hours
MBD4
2 hours
6 hours
24 hours
USP7
2 hours
6 hours
24 hours

Fig. S16 (cont.)

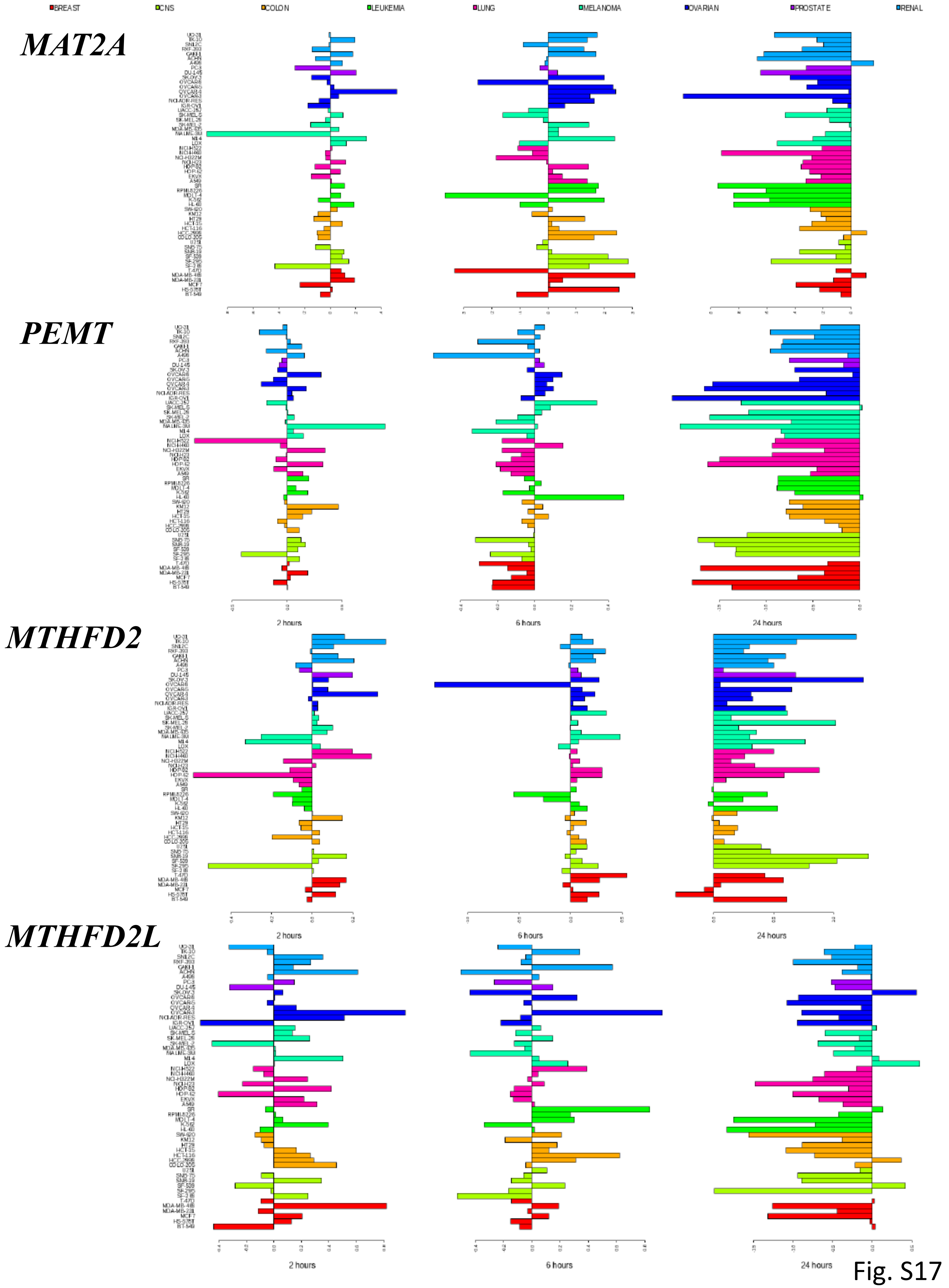

BREAST
CNS
COLON
LEUKEMIA
LUNG
MELANOMA
OVARIAN
PROSTATE
RENAL
MAT2A
PEMT
MTHFD2
MTHFD2L
2 hours
6 hours
24 hours

Fig. S17
